# Decomposition of HCN during Experimental Impacts in Dry and Wet Planetary Atmospheres

**DOI:** 10.1021/acsearthspacechem.4c00064

**Published:** 2024-05-24

**Authors:** Antonín Knížek, Lukáš Petera, Vojtěch Laitl, Martin Ferus

**Affiliations:** † J.Heyrovský Institute of Physical Chemistry, Czech Academy of Sciences, Dolejškova 2155/3, CZ18223 Prague, Czech Republic; ‡ Department of Inorganic Chemistry, Faculty of Science, Charles University, Hlavova 8, CZ12800 Prague, Czech Republic; § Faculty of Science, University of Antwerp, Groenenborgerlaan 171, BE2020 Antwerpen, Belgium

**Keywords:** planets and satellites: atmospheres, planets and satellites: general, astrochemistry, plasma, shock waves

## Abstract

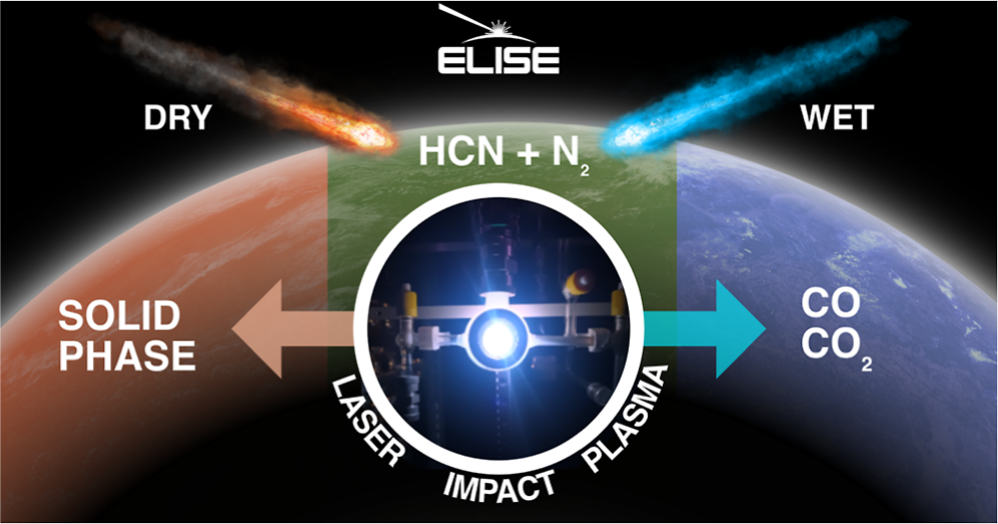

Hydrogen cyanide (HCN), a key molecule of significant importance in contemporary perspectives on prebiotic chemistry, originates in planetary atmospheres from various processes, such as photochemistry, thermochemistry, and impact chemistry, as well as from delivery by impacts. The resilience of HCN during periods of heavy bombardment, a phenomenon caused by an influx of material on unstable trajectories after accretion, remains relatively understudied. This study extensively investigates the stability of HCN under impact conditions simulated using a laboratory Nd:YAG laser in the ELISE experimental setup. High-resolution infrared spectroscopy was employed to monitor the gas phase composition during these simulations. Impact chemistry was simulated in bulk nitrogen atmospheres with varying mixing ratios of HCN and water vapor. The probed range of compositions spans from ∼0 to 1.8% of HCN and 0 to 2.7% of H_2_O in a ∼1 bar nitrogen atmosphere. The primary decomposition products of HCN are CO and CO_2_ in the presence of water and unidentified solid phase products in dry conditions. Our experiments revealed a range of initial HCN decomposition rates between 2.43 × 10^15^ and 5.17 × 10^17^ molec J^–1^ of input energy depending on the initial composition. Notably, it is shown that the decomposition process induced by the laser spark simulating the impact plasma is nonlinear, with the duration of the irradiation markedly affecting the decomposition rate. These findings underscore the necessity for careful consideration and allowance for margins when applying these rates to chemical models of molecular synthesis and decomposition in planetary atmospheres.

## 1 Introduction

In the light of research over the last six decades, hydrogen cyanide (HCN) has risen up as a molecule of special prominence in the fields of astronomy, astrochemistry, and origin of life studies. Besides its occurrence in the contemporary Earth,^1−3^ HCN was discovered in the atmospheres of Jupiter^4,5^ and plays a significant role in the atmospheric photochemistry of Saturn’s moon Titan.^6^ Furthermore, it was observed in a number of cometary comas,^7^ interstellar molecular clouds,^8^ planetary nebulae,^9^ and protoplanetary discs.^10^ Its detection has also been recently confirmed in the atmospheres of WASP-76b^11,12^ or HD 189733 b^13^ and suggested in the atmosphere of a hot (2000 K) super-Earth 55 Cancri e.^14^

The widespread occurrence of HCN can be attributed to its electronic structure, which is characterized by a robust, high-energy triple bond (891 kJ mol^–1^). This structural feature renders HCN a stable product in a diverse array of chemical reactions involving hydrogen-, carbon-, and nitrogen-bearing compounds across a wide spectrum of environmental conditions. Due to the favorable symmetry of the 3σ HOMO orbital of CN^–^/^•^CN stemming from similar C and N orbital energies, HCN is also generally considered to easily undergo polymerization leading to a variety of HCN-derived polymers of prebiotic importance (see Ruiz-Bermejo et al.^15^ and references therein).

Therefore, HCN, CN^–^, and ^•^CN are considered to play a key role in a variety of prebiotic scenarios.^16−19^ Formation of adenine—a HCN pentamer—under prebiotically relevant conditions was demonstrated by Oro and Kamat^20^ as early as 1960. Since then, HCN-based prebiotic synthesis has been shown to lead to the formation of all the canonical nucleobases,^21,22^ amino acids,^20^ and sugars^17^ starting from mixtures containing HCN exposed to simulated hydrothermal heating, UV photochemistry,^19,23,24^ lightning, or impacts.^25^

On planets, various sources and sinks of HCN have been identified: atmospheric HCN can be produced by UV photochemistry,^26−30^ by interaction of the atmosphere with energetic particles,^31^ by atmospheric electricity,^32,33^ or by other nonequilibrium chemistry.^34^ Asteroid and cometary impacts are also capable of producing HCN—both by reprocessing of the initial atmosphere^16,35,36^ and by decomposition of the carbonaceous matter in the impactor itself.^37^ It has been shown that HCN is produced most efficiently in atmospheres containing reduced C-bearing compounds, such as CH_4_ or C_2_H_2_, with a yield of HCN proportional to the atmospheric C/O ratio (see Rimmer and Rugheimer^38^ and references therein). The sinks of HCN are similar: UV photochemistry, interaction with energetic particles, lightning, and impacts. In addition, HCN can be removed from an atmosphere by adsorption onto the planetary surface or dissolution in the hydrosphere.^39^

The HCN atmospheric lifetime can be satisfactorily constrained for scenarios involving sinks like photochemistry, ion chemistry, or chemistry at phase boundaries (deposition, rainout, etc.). However, in the case of impact shocks, the constraints must still be established.

Due to the complex physical nature of an impact, a thorough experimental simulation of this phenomenon is difficult to perform. Instead, impact events have so far been experimentally simulated using a range of techniques that generate some of the typical impact characteristics, such as high pressures (tens of GPa), high temperatures (thousands of K), plasma formation, and high-velocity (tens of km s^–1^) bodies interaction. The experimental simulations include techniques such as hyper-velocity gun experiments,^40^ experiments with high-pressure press,^41^ shock-tubes experiments,^42,43^ air-plasma flows^44−48^ and, as in this paper, laser-induced dielectric breakdown (LIDB).

The concept of simulating atmospheric impact shock plasma using laser-induced dielectric breakdown (LIDB) was initially proposed by Rae and Hertzberg^49^ in 1964. This methodology was later employed for the first time in simulating impact evaporation by Hapke et al.^50^ in 1975. The key effects of LIDB include a shock rise in temperature, formation of a plasma fireball, generation of a shock wave, collisional energy transfer, and emission of radiation from atomic and molecular species during their relaxation from highly excited states.^51,52^ In the present study, we use an Nd:YAG laboratory laser to initiate LIDB in mixtures containing HCN, H_2_O vapor, and N_2_ to estimate the stability of atmospheric HCN during impact events.

A theoretical study on this matter by Todd and Oberg^39^ suggests that HCN survival mainly depends on whether the impact is hot enough to decompose H_2_O into reactive radicals. The authors discuss their model results in relation to the impactor mass, velocity, and impact angle. Following their findings, we focus this study on the experimental investigation of the stability of atmospheric HCN during simulated impacts in a ∼1 bar N_2_ atmosphere batched with HCN (up to 1.8% of inlet pressure) and H_2_O (up to 2.7%). To study HCN’s behavior separately and to prevent its formation from C-precursors (mainly CH_4_), we investigate a system containing HCN as the only C-bearing species. Water is then an oxidation agent. Generally, it is unlikely to find a stable planetary atmosphere with significant amounts of both HCN and H_2_O at the same time because photochemistry and thermochemistry would lead to a fast oxidation of the HCN. However, water can be brought to the HCN-rich atmosphere by an impactor^39^ or released by volcanism.^53^ N_2_ was selected as the buffer gas because it is the most commonly assumed background gas on rocky planets that supports habitability^54^ and a likely major constituent of many terrestrial exoplanet atmospheres.^55^ We discuss the rates of HCN decomposition in relation to the initial amount of HCN and H_2_O contents. Using planetary chemistry assumptions from previous studies, we also estimate HCN decomposition fluxes for the environments of early Earth, Venus, and Mars.

## 2 Experimental Apparatus

**Figure 1:**
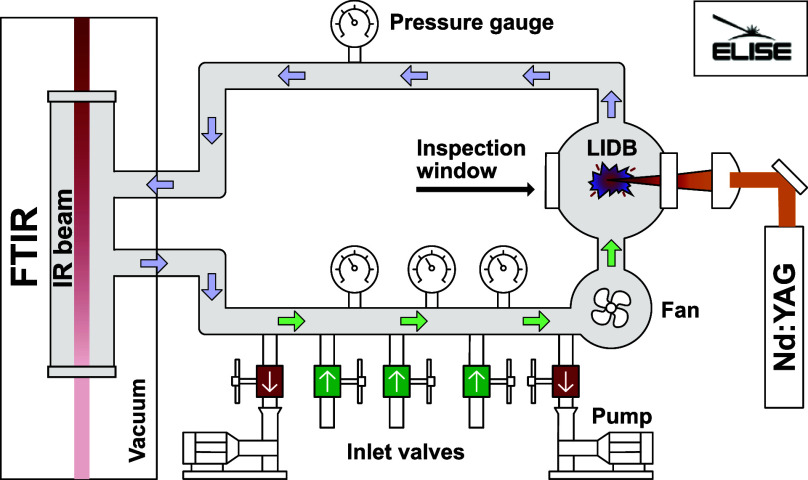
Experimental laboratory impact simulator for exoplanets (ELISE). This apparatus is designed for laser simulations of impact chemistry. The apparatus contains an Nd:YAG laser, a FTIR spectrometer, a window for UV–vis emission spectra acquisition, an irradiation cell, a Teflon fan, several capacitance manometers for pressure measurement, and inlets and outlets for gas and vacuum handling.

Impact plasma was simulated by LIDB using a laboratory Nd:YAG laser. Our experimental procedure combines closed, isochoric batch reactor with active gas flow, high-resolution Fourier transform infrared spectroscopy (FTIR) online detection, and analytical calibration-free data evaluation process. The experiments were performed on the ELISE experimental apparatus shown in Figure 1.^56^ This modular apparatus is designed for laser irradiation of samples with direct access to FTIR absorption and UV–vis emission spectroscopy for online analysis. For the purpose of this study, we used an irradiation cell designed for gas phase LIDB (see Supporting Information). The total volume of the studied atmospheric mixture in the apparatus was ∼850 mL. Prior to each experiment, the apparatus was evacuated to a dynamic background pressure of about 0.03 Torr. Blank FTIR measurements were taken before each experiment. After that, the apparatus was filled with the appropriate atmosphere and thoroughly mixed with an in-built fan for ∼5 min. The exact initial composition was then determined by FTIR. LIDB was performed with a pulsed Nd:YAG laser system set to 450 mJ pulse energy, 10 Hz at 1064 nm wavelength, with 6 ns pulse width. Laser pulses were focused into the irradiation cell using a 10.0 cm N-BK7 laser lens (Edmund Optics) through a N-BK7 laser window (Edmund Optics). During the whole LIDB process, the gaseous mixture was constantly mixed at ∼3 mL s^–1^ flow (see Supporting Information of Petera et al.^56^ for details). After a set interval, the irradiation was stopped, the sample mixture was thoroughly mixed for ∼2 min, and the immediate gas phase composition was measured by FTIR. Each FTIR measurement takes about 2 min, during which the samples were tested to be stable. The measurements were taken on a Bruker IFS 125HR spectrometer (Bruker GmbH, Germany) equipped with a liquid nitrogen cooled MCT detector. The spectra were recorded in a 1000–4800 cm^–1^ range with the resolution 0.1 cm^–1^. Twenty-five scans were averaged in each measurement to get an optimal signal-to-noise ratio.

The data were analyzed using the spectr package.^57^ This package fits HITRAN^58^ molecular parameters to the data and calculates the column density for the selected molecular species. The fit includes optimization of column density for a species, the temperature, and the total pressure (to account for pressure and temperature broadening). The fit uses a Boltzmann distribution for the population of states, and the retrieved temperature is used to verify the stability of the system. Air broadening coefficients were used for the pressure-induced broadening, and the Voigt profile was used to fit the spectral lines. Partial pressures were then determined from the retrieved column densities by using the ideal gas law and the known optical path of the measurement cell (26.7 cm). This approach enables rapid data analysis without the need for analytical calibration during the data evaluation process.

## 3 Performed Experiments

In this study, we performed two sets of experiments containing various amounts of HCN and H_2_O vapor in N_2_ bulk gas. The first set (experiments 1–9 in Table 1) consisted of 9 experiments dedicated to elucidating the HCN decomposition rate coefficients. These experiments contained an approximately constant (0.9–1.50 Torr) amount of HCN and a variable amount of H_2_O vapor (0.09–13.25 Torr). Each sample was filled with N_2_ up to a pressure of 720 Torr.

**Table 1:** Initial Composition and Irradiation Times of the Performed Experiments^a^

experiment	*p*_HCN_ (Torr)	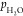 (Torr)	*p*_N_2__ (Torr)	irradiation time (s)	C/O
1	0.923 ± 0.004	10.98 ± 0.01	up to 720	7000	0.1
2	1.252 ± 0.004	13.25 ± 0.01	up to 720	7000	0.1
3	1.422 ± 0.004	6.677 ± 0.005	up to 720	7000	0.2
4	1.569 ± 0.003	3.772 ± 0.003	up to 720	7000	0.4
5	1.519 ± 0.003	1.472 ± 0.002	up to 720	7000	1
6	1.353 ± 0.003	0.701 ± 0.001	up to 720	7000	1.9
7	1.079 ± 0.002	0.268 ± 0.001	up to 720	7000	4
8	1.522 ± 0.003	0.194 ± 0.001	up to 720	7000	7.8
9	1.552 ± 0.003	0.0978 ± 0.0008	up to 720	7000	15.9
10	0.473 ± 0.007	21.83 ± 0.03	up to 720	420	0.02
11	1.332 ± 0.007	22.57 ± 0.03	up to 720	420	0.1
12	1.346 ± 0.004	9.404 ± 0.007	up to 720	420	0.1
13	2.734 ± 0.009	16.26 ± 0.02	up to 720	420	0.2
14	1.452 ± 0.004	4.523 ± 0.003	up to 720	420	0.3
15	5.97 ± 0.03	17.96 ± 0.02	up to 720	420	0.3
16	5.84 ± 0.02	21.30 ± 0.03	up to 720	420	0.3
17	0.684 ± 0.003	1.150 ± 0.002	up to 720	420	0.6
18	3.079 ± 0.007	5.566 ± 0.004	up to 720	420	0.6
19	13.0 ± 0.2	18.17 ± 0.02	up to 720	420	0.7
20	0.500 ± 0.003	0.655 ± 0.002	up to 720	420	0.8
21	6.29 ± 0.02	5.709 ± 0.004	up to 720	420	1.1
22	3.364 ± 0.008	2.686 ± 0.003	up to 720	420	1.3
23	1.527 ± 0.004	1.074 ± 0.002	up to 720	420	1.4
24	1.521 ± 0.004	0.419 ± 0.002	up to 720	420	3.6
25	3.331 ± 0.007	0.911 ± 0.002	up to 720	420	3.7
26	11.89 ± 0.05	2.472 ± 0.003	up to 720	420	4.8
27	0.785 ± 0.003	0.090 ± 0.001	up to 720	420	8.7
28	1.525 ± 0.004	0.099 ± 0.001	up to 720	420	15.4
29	3.229 ± 0.006	0.110 ± 0.001	up to 720	420	29.4
30	6.68 ± 0.02	0.123 ± 0.001	up to 720	420	54.3
31	12.5 ± 0.2	0.104 ± 0.001	up to 720	420	120.2

a C/O ratio is indicated for each experiment.

Each mixture was then exposed to the experimental LIDB. The irradiation was stopped to acquire the FTIR spectra at set time intervals of 0, 60, 120, 240, 500, 900, 1300, 2400, 4000, and 7000 s. At 7000 s (total input energy 31,500 J), HCN was fully decomposed in all experiments. Data from these experiments were used to extract the system’s rate coefficients of HCN and H_2_O decomposition and production formation. The second set (experiments 10–31 in Table 1) covered a broader range of both HCN (0.47 and 13.05 Torr) and H_2_O vapor (0.09 and 21.83 Torr) initial amounts. The irradiation time of these experiments was 420 s (total of 1890 J), which is the approximate half-life of HCN from experiments 1–9. These experiments were used to test the validity limits of the rate coefficients as described below.

## 4 HCN Decomposition Products

The decomposition rate of HCN under the N_2_-dominated atmosphere was monitored with high-resolution Fourier-transform infrared spectroscopy. The time-dependent FTIR monitoring proved that HCN is unstable under the simulated impact conditions and can fully decompose over a sufficiently extended time period. In a dry (minimum H_2_O presence) atmosphere, HCN is decomposed into IR invisible products accompanied by formation of an unidentified black-brownish solid settling on the apparatus walls. Increasing the initial amount of H_2_O vapor leads to significant CO and CO_2_ formation, a smaller amount of the solid residue, and an overall faster decomposition of HCN. Water itself also decomposes to IR invisible products, likely H_2_ and O_2_.^59^

**Figure 2:**
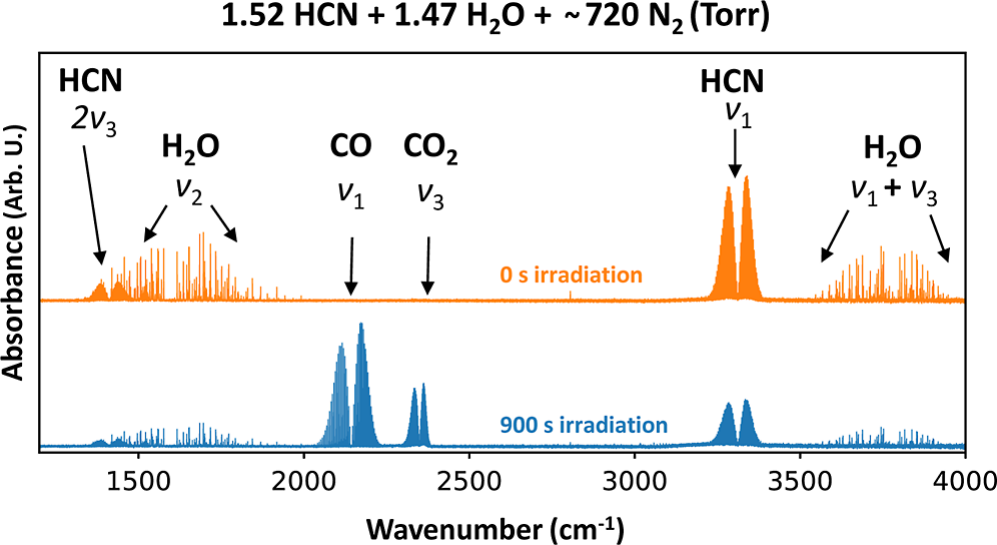
Example FTIR spectrum recorded in a mixture of HCN, H_2_O, and N_2_ (composition 0.16:0.46:100) after 120 s of laser irradiation simulating the impact plasma. Cumulative energy delivered to the system at this stage was 540 J. Absorption bands belong to HCN and H_2_O (reactants), and CO and CO_2_ (products).

A typical FTIR spectrum is shown in Figure 2. The upper (orange) spectrum represents the mixture at the beginning of the experiment (0 s of irradiation), while the bottom one (blue) represents the mixture after 900 s of irradiation (9000 laser pulses) corresponding to the energy of 4000 J delivered to the system. Here, the decrease in HCN and H_2_O and increase in CO and CO_2_ signals are clearly seen after the irradiation.

**Figure 3:**
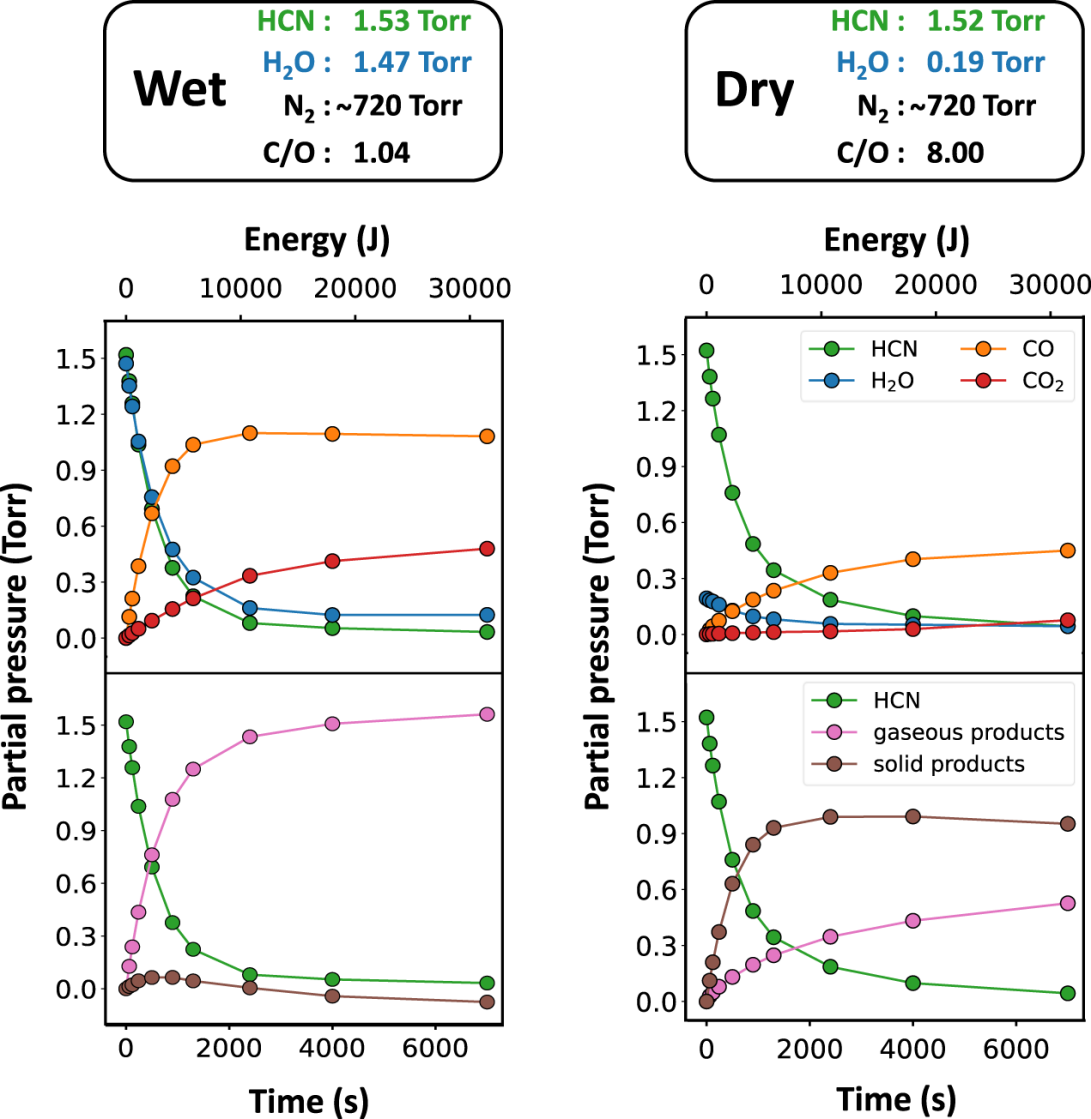
Evolution of the gas mixture throughout an experiment. This typical plot shows the decomposition of HCN and H_2_O and the formation of CO, CO_2_, and the solid phase. Experimental points were connected with lines for clarity.

Partial pressures obtained from the IR spectra were plotted against the irradiation time. Typical plots obtained from the experiment are shown in Figure 3. The figure shows two extreme-case scenarios: impact into a dry atmosphere and impact into an atmosphere with HCN/H_2_O ∼1. The top plots in each column show the time evolution of HCN, H_2_O, and CO and CO_2_. Since both H_2_O and HCN decompose as well, the total amount of CO and CO_2_ does not equal the amount of decomposed HCN. The bottom plots of Figure 3 show calculated amounts of decomposed HCN and the distribution of carbon between the formed gas and solid C-bearing products. Clearly, impacts into a “wet” atmosphere, which possesses the initial C/O ratio of 1.04, result in the majority of the carbon ending up as CO and CO_2_, while in contrast, dry impacts (initial C/O ratio 8.00) exhibit significant formation of the solid phase. In this calculation, CO and CO_2_ are the only considered gas phase products. Although CH_4_ and C_2_H_2_ were detected in three experiments (with the highest C/O ratios), their amount was negligible. Hydrocarbons, such as C_2_H_4_ or C_2_H_6_, were not detected. Their detection limits reach ∼10^–2^ Torr, and therefore, if present, they would also constitute a negligible part of the product mixture. No IR invisible carbonaceous volatiles are expected either. With the naked eye, the forming solid phase settles down on the experimental apparatus wall and its amount increased with the decreasing H_2_O initial content. This suggests that the carbon missing from the gas phase is indeed stored in the solid phase. The likely constituents of the solid phase are soot, tholins, and various CN polymers.^60^ The amount of the solid phase was calculated as the initial amount of carbon minus the immediate amount of carbon observed in the gas phase. The calculation is shown in the Supporting Information. A systematic study of the solid phase composition is beyond the scope of this study and is intended for future work. Discussion and a photo of the solid phase are included in the Supporting Information. For details, see Supporting Information.

## 5 System Rate Coefficients

The initial series of experiments (1 and 9) aimed at determining the global rate coefficients for reactions transforming the studied mixtures (HCN, H_2_O) into the observed products (CO, CO_2_, and carbonaceous refractory material deposited in the cell). These coefficients characterize the overall phenomenological behavior of our experimental system and should be understood as system rate coefficients, distinct from rate constants of elementary reactions. As such, they can be used for quantification of the yields of the HCN decomposition and yields of products formation within the range of our experimental parameters.

The system’s behavior is represented by a set of four equations, empirically proposed to elucidate the global reaction pathways in the examined system
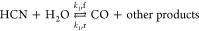
1
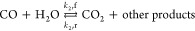
2
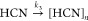
3
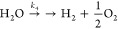
4

To simplify the description, it should be noted that “other products” involve species and compounds resulting from complex chemistry involving H, OH, NH_*x*_, or CN. These processes have been detailed in several of our previous studies,^16,22,35,61^ as well as in comprehensive analyses of reaction steps by other researchers.^62−64^ [HCN]_*n*_ represents solid phase refractory carbon material resulting from HCN decomposition.

**Figure 4:**
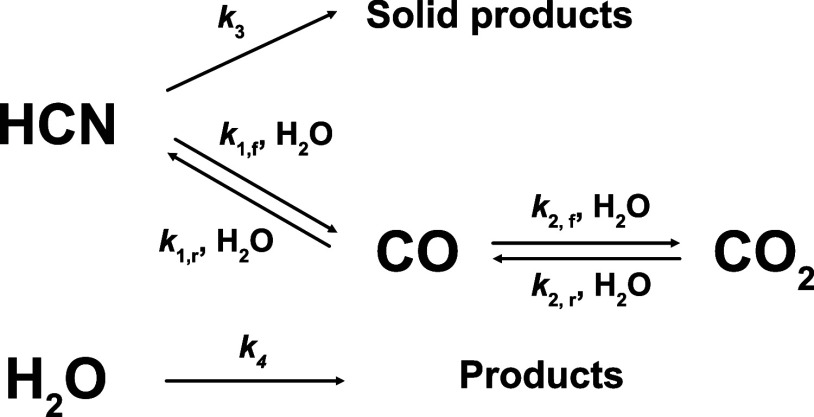
Schematic depiction of the reactions used in this model.

This chemistry is schematically outlined in Figure 4. Comprehensive details, including the rate equations and their implementation, are provided in the Supporting Information.

The predominant pathway for HCN decomposition leads to the formation of CO, which is then oxidized to CO_2_. Both of these reactions are reversible and depend on the H_2_O content. These reactions are described by rate coefficients *k*_1,f_, *k*_1,r_, *k*_2,f_, and *k*_2,r_. Additionally, HCN decomposes to solid products through an irreversible reaction with the rate coefficient *k*_3_. Finally, H_2_O undergoes irreversible decomposition into H_2_ and O_2_, represented by the constant *k*_4_.

The rate constants were calculated by fitting a set of differential equations to the data. A significant advantage of our approach is that the calculation involved four global differential equations considering the adsorption of all molecules, which significantly influences their experimental values. In short, for example, observation suggests that at low partial pressure of water vapor, as much as 2/3 of the present water is adsorbed on the apparatus walls. Fitting reaction rates from partial pressures therefore requires the treatment of this effect. Since this adsorption is nearly impossible to quantify analytically (not only do all present molecules adsorb to the surface with different affinity but there is also competition for adsorption sites, multilayer adsorption, etc.), we instead treat the effect statistically. For details, see the Supporting Information.

Table 2 presents the final system rate coefficients along with their standard deviations. It is important to note that these standard deviations were propagated through the entire calculation, indicating that the data manipulation is both reasonably accurate and justified. Additionally, potential sources of error are discussed in detail in the Supporting Information.

**Table 2:** Final Rate Constants Obtained from the Fit of All Our Data

rate constant	value
*k*_1,f_	(3 ± 2) × 10^–^^4^ Torr^–^^1^ s^–^^1^
*k*_1,r_	(6 ± 4) × 10^–^^4^ Torr^–^^1^ s^–^^1^
*k*_2,f_	(1.2 ± 0.4) × 10^–^^3^ Torr^–^^1^ s^–^^1^
*k*_2,r_	(5 ± 1) × 10^–^^3^ Torr^–^^1^ s^–^^1^
*k*_3_	(8 ± 6) × 10^–^^4^ s^–^^1^
*k*_4_	(2.4 ± 0.6) × 10^–^^4^ s^–^^1^

Furthermore, as outlined above and detailed in Table 1, we conducted 21 additional single-point experiments (continuous 420 s of irradiation and FTIR analysis before and after) with a broader range of initial HCN and H_2_O concentrations. The initial composition at the start of each experiment was used to predict the gas phase composition after 420 s of irradiation with the kinetic model. The computed predictions were subsequently compared with the corresponding experimental results. The experiments covered a parameter space of initial partial pressures 0–13 Torr (0–1.8%) for HCN and 0–20 Torr (0–2.7%) for H_2_O. Detailed results are shown in the Supporting Information. This expansion of the experimental parameter space broadens the applicability of global rate coefficients for investigating plasma chemistry in planetary atmospheres. Since the range covers the water vapor content up to the saturated vapor pressure and HCN content up to 1.8%, this range should be sufficient to describe the HCN and H_2_O contents in the vast majority of known planetary systems. As will be discussed later, however, our experiments describe an isolated process. Real planetary atmospheres are more complex, and proper application of our results should be done through chemical models including other species as well.

## 6 HCN Decomposition Mechanism

Similar to the results of Todd and Oberg^39^ we observed that HCN is decomposed in the presence of H_2_O, but we also observed its decomposition without any water present. In their study, Todd and Oberg^39^ demonstrated that HCN decomposition is driven by OH radicals originating from the plasmolysis of water. It is important to note that their simulation was conducted at lower temperatures compared to those under our experimental conditions. In actual planetary atmospheres, other species may also play a part, including other oxygen-bearing species. To gain further insights into the decomposition mechanism in the laboratory plasma, we conducted indicative UV–vis emission measurements of our LIDB.

Emission spectra of the LIDB were recorded with a high-resolution Butterfly Echelle spectrograph equipped with an Andor ICCD camera. The spectrograph operates in the UV (192–433 nm) and vis (425–750 nm) regions, at respective spectral resolutions of 13–31 pm (resolving power 14,000) and 21–37 pm (resolving power 20,000). Prior to data collection, the Butterfly spectrometer was calibrated with a Hg lamp.

**Figure 5:**
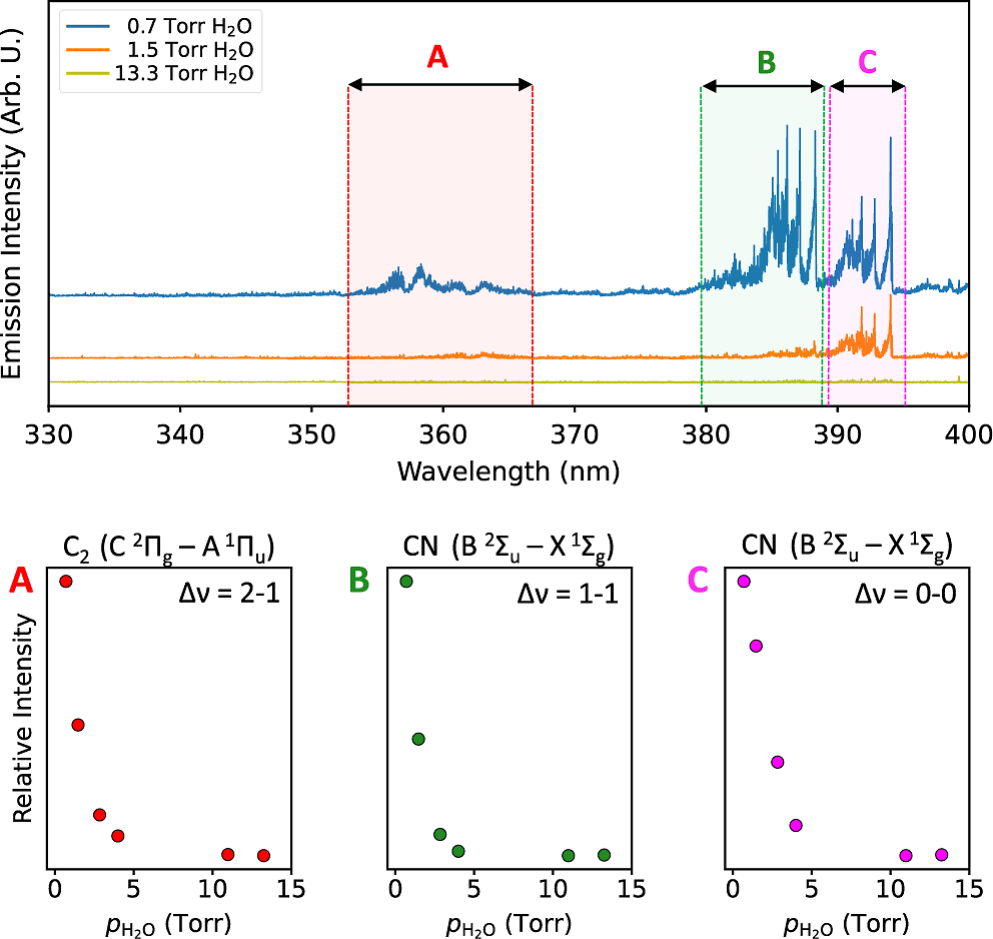
Top panel shows UV–vis emission spectra of HCN + H_2_O containing mixtures with the observed signals of C_2_ and CN indicated (A–C). The three bottom panels then show integrated band intensities for the three observed bands. The figure in general reveals a systematic depletion of C_2_ and CN emission signals with the increasing amount of water in the sample.

The acquired spectra showed emission bands of typical products, such as the CN radical and C_2_ radical, but emission from the OH radical typically observed for example in our previous studies on the decomposition of formamide^65^ or other oxygen species (O^+^, O_2_^+^ etc.) remained undetected, possibly due to low dynamical concentration. Another likely reason for the nondetection of OH is the low emissivity of the OH radical band situated around 309 nm. Also, applying our isochoricity constraints to the thermodynamic reasoning of Shabanov and Gornushkin^66^ implies that the energy-dense OH radicals would likely be localized around the LIDB’s focal plane itself. Such spatial confinement may then lead to the obliteration of OH signals by those of the ubiquitous CN and C_2_ emitters. The observed behavior of both CN and C_2_ radicals correlates with the other findings in this paper. Figure 5 reveals a systematic depletion of CN (and C_2_) signals when increasing the initial H_2_O content. The top panel shows a decrease in the CN and C_2_ signals in the recorded spectra, while the bottom panel shows the decrease in signal intensity of integrated intensities of three spectral bands. The presence of OH radicals in the plasma is therefore likely, but their detection is not possible with our current setup. An in-depth, systematic UV–vis emission study of this LIDB, too, is over the scope of this publication and will require dedicated experimental campaigns alongside with apparatus modifications.

From a different point of view, HCN decomposes in the plasma. CN radicals then react to form the solid phase, which is likely composed of tholins. Reactions of H and CN radicals in the collisional environment of the plasma must also inevitably lead to the reformation of some of the decomposed HCN. Present OH radicals in the system then “compete” with H radicals in reactions with the CN radicals to form such products as CO and CO_2_.

**Figure 6:**
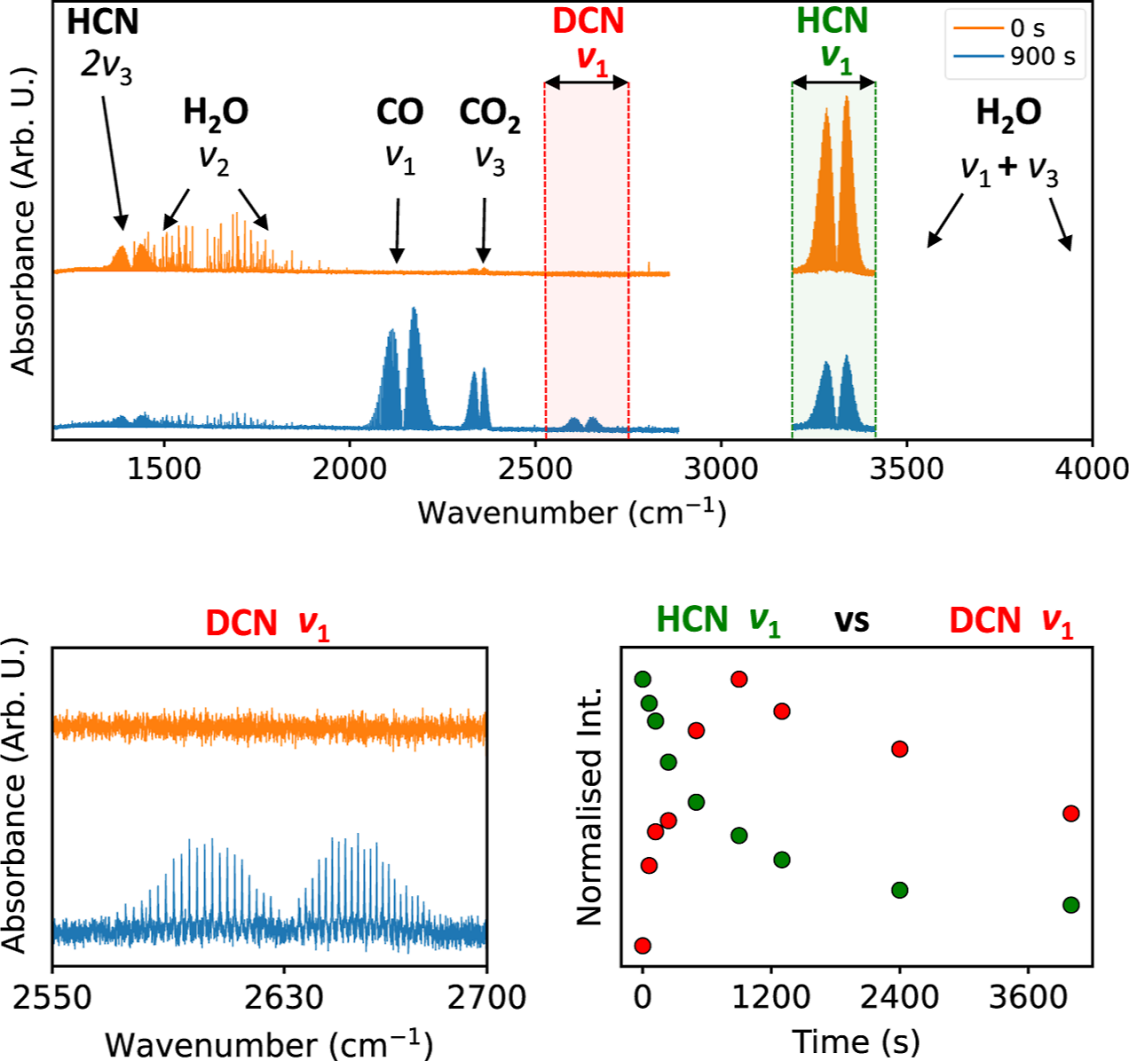
Top panel shows absorption spectra of the gas phase of HCN, D_2_, and H_2_O mixtures after 900 s of irradiation. Absorption bands of HCN and DCN are indicated. The bottom left panel shows a zoom-in of the DCN ν_1_ absorption band. The bottom right panel then shows integrated HCN and DCN band intensities at different irradiation times. Note that the intensities were normalized for graphical clarity.

To illustrate this process, additional experiments with Nd:YAG laser irradiation of a mixture of ∼1.5 Torr HCN, ∼2.0 Torr D_2_, and ∼720 Torr N_2_ were performed. The present D_2_ is expected to split into D radicals in the plasma. If HCN is reformed in the plasma by collisions of CN with H, then the heavier isotopologue DCN should also form besides HCN as a result of a competitive recombination of both H and D radicals with the CN radical. As shown in Figure 6, the absorption band of DCN ν_1_ (2630 cm^–1^) indeed emerged during the experiment. The top panel shows spectra of the mixture before and after 900 s of irradiation with the position of both HCN and DCN absorption bands indicated. The maximum DCN band intensity was observed after 900 s of irradiation. We remark that both the HCN and DCN bands intensities were normalized in order to clearly show their time profiles. The DCN band intensity is in reality much lower than that of HCN, and their mutual ratio at 900 s (DCN max) is HCN/DCN = 6.2. In any case, this experiment proves that the reaction of CN with other particles in the plasma is a competitive process and suggests that in atmospheres with higher H/D content, HCN should be more efficiently reformed and therefore more stable during impacts.

## 7 HCN Decomposition Rate in Planetary Atmospheres

The system rate coefficients calculated above provide insights into the mechanism of the HCN decomposition process. However, another simpler and more direct approach to assessing the stability of HCN during impacts is by calculating the HCN decomposition rate, *S*_HCN_. We note that this approach is a simplification associated with significant risk of misinterpretation (as will be discussed below), but it is a common practice in the existing literature, and the calculation is made here for comparison sake. As the most commonly used unit of a source/sink of a given molecule is the molecule per joule of input energy (molec J^–1^), the HCN decomposition rate was calculated using the following formula
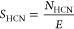
5where *N*_HCN_ is the number of decomposed molecules of HCN, *E* (J) is the energy input to the system, and *S*_HCN_ is the HCN decomposition rate (sink) in molec J^–1^. Next, *N*_HCN_ can be obtained from the experiment as
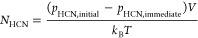
6where *p*_HCN,initial_ and *p*_HCN,immediate_ are the initial and immediate (at the time of evaluation) partial pressures of HCN in Pa, respectively, *V* = 8.5 × 10^–4^ m^3^ is the volume of the apparatus, *k*_B_ is the Boltzmann constant, and *T* = 300 K is the temperature. The energy input can be calculated as
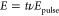
7where *t* (s) is the irradiation time, ν = 10 Hz is the laser repetition frequency, and *E*_pulse_ = 0.45 J is the energy of one laser pulse.

**Figure 7:**
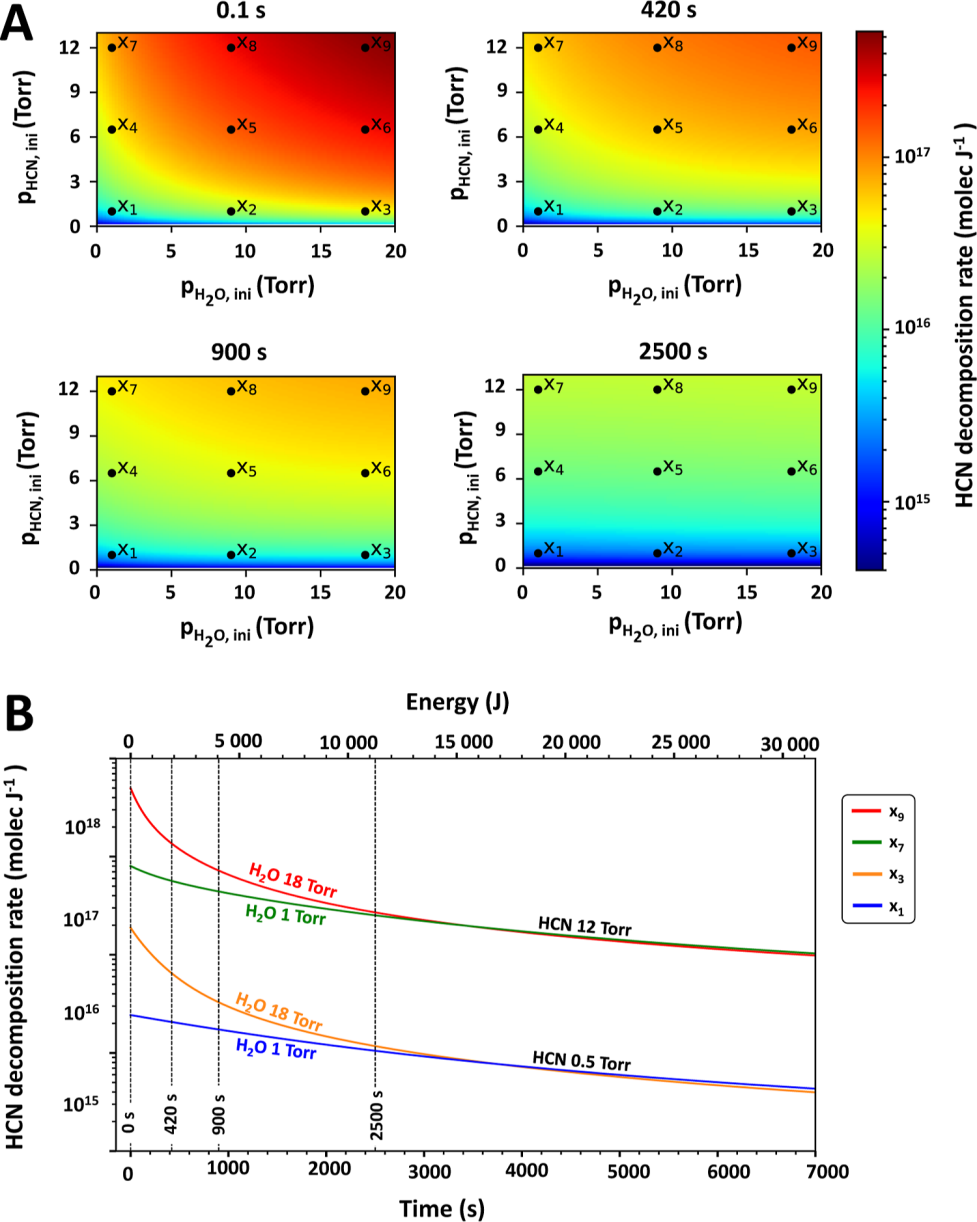
Panel A of this figure shows HCN decomposition rates calculated with our model. The four 2D colormaps show the HCN decomposition rates as a function of the initial composition. The four panels each represent snapshots of the decomposition rates at indicated irradiation times. The 2D colormaps also have points x_1_–x_9_ indicated. Panel B shows calculated HCN decomposition rates at fixed compositions as a function of time. The fixed compositions are at points x_1_, x_3_, x_7_, and x_9_. The figure shows that varying H_2_O content changes the HCN decomposition rate at the beginning and slowly converges toward a value determined by the initial amount of HCN.

The calculated decomposition rate *S*_HCN_ clearly depends on the selected irradiation time and initial composition. Fundamentally, the rate also depends on the geometry of the experiment and experimental arrangement. The rates are depicted in Figure 7. Panel A shows four subplots of calculated HCN decomposition rates (molec J^–1^ of input energy) in the whole applicable range of HCN (∼0–13 Torr) and H_2_O (0–20 Torr) initial partial pressures. Each frame corresponds to a distinct irradiation time: 0.1 s (at the beginning −1 laser pulse), 420 s (at the time as experiments 10–31 from Table 1), 900, and 2500 s, where the majority of HCN is destroyed in all experiments. Each frame in Figure 7 also shows 9 marked points (x_1_–x_9_). Panel B of Figure 7 then shows the HCN decomposition rate calculated for initial conditions from points x_1_–x_9_ at all experimental times. These two graphs illustrate that the decomposition rate is the highest at the beginning (0.1 s interval corresponding to one laser pulse delivered to the system) when both HCN and H_2_O are present in their respective highest amounts. It is also clearly seen that these initial decomposition rates depend on the initial HCN/H_2_O ratio, as increasing the amount of water enhances the reaction channel leading to CO and CO_2_. The maximum decomposition rate in our experiment reaches 5.37 × 10^17^ molec J^–1^ of input energy for the initial HCN and H_2_O partial pressures of 13.0 and 20.0 Torr. The minimum observed decomposition rate was 2.43 × 10^15^ molec J^–1^ of input energy for the initial HCN and H_2_O partial pressures of 0.5 and 0 Torr (as shown in Table 3). Similar plots with snapshots from the model for different reactants, products, and product combinations are shown in the Supporting Information.

**Table 3:** HCN Decomposition Rates

	*p*_HCN,i_ (Torr)	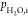 (Torr)	HCN decomposition rate (molec J^–^^1^) of input energy			
			0.1 s	106 s (ref (67))	420 s	4000 s
max	12.5	20	5.17 × 10^17^	3.24 × 10^17^	1.45 × 10^17^	1.84 × 10^16^
min	0.5	0	2.43 × 10^15^	2.33 × 10^15^	2.07 × 10^15^	7.29 × 10^14^

For comparison, McKay and Borucki^67^ performed a similar experiment and reported a HCN production rate 5_-2.5_^+5^ × 10^17^ molec J^–1^ from CH_4_. Comparison between our maximum destruction rate and this production rate might suggest that the HCN destruction and production rates are comparable. Experiments conducted by McKay and Borucki^67^ and Scattergood et al.^68^ follow a similar philosophy to ours—a gaseous mixture representative of a planetary atmosphere irradiated with a laboratory laser. They also utilize an Nd:YAG laser in a manner similar to ours, although their pulse duration was 15 ns (compared to our 6 ns) and their pulse energy was 200 mJ (compared to our 450 mJ). In contrast to us, the authors also use a 1 bar total pressure with 3.0% of CH_4_ as their mixture, as opposed to our experiments with ∼0.2% of HCN (i.e., about 1 order of magnitude smaller carbon feedstock). Our data depicted in Figure 7 show that the relationship between the HCN decomposition rate and irradiation time is not linear. Contrary to our results, Scattergood et al.^68^ claim to observe linear HCN yields (cf. Figure 2 of their original paper). However, our experiments suggest show that this (near-)linearity is only observable at high inlet concentrations and low irradiation times (their less than 240 s, as opposed to our 7000 s in this paper). As our data show, only at lower concentrations of reactants are the actual kinetics revealed. At the same time, sub-1% mixing ratios of molecules such as HCN or CH_4_ are much more likely than 3% content in actual planetary atmospheres. Careful description of the kinetics is therefore very important, and the usage of simple linear fits, as applied in the past due to lack of relevant laboratory data, is insufficient. These discrepancies suggest that plasma chemistry in planetary atmospheres, with its broad complexity, can be more accurately constrained through a combination of careful experimental studies that map a sufficient parameter space and detailed models of their chemistry.

## 8 Astrochemical Context

The parameter space of atmospheric compositions explored in this study provides a comprehensive framework for applying this chemistry to complex models of planetary atmospheres affected by impact shocks or other plasma events. In our previous research, HCN was identified as a common and significant product of the impact plasma reprocessing of planetary atmospheres.^16,56^ This study centered on examining the stability of HCN in a nitrogen (N_2_) bulk atmosphere, with variations in the initial partial pressures of HCN and H_2_O. The significant presence of N_2_ aligns with dilution kinetics approaches, enhancing the experiment’s representativeness of planetary atmospheric conditions. Drawing upon a substantial number of experimental results, we observe that HCN decomposes in both dry and wet atmospheric compositions. The overall decomposition rate of HCN depends on both the initial amount of HCN and the initial HCN/H_2_O (C/O) ratio. The dependence on the initial amount of HCN could be explained by the different amounts of HCN available for interaction with the LIDB spark. Given that the plasma volume is expected to be constant, a higher initial concentration of HCN results in a greater number of HCN molecules being affected by the plasma spark. This observation has implications for impact plasma processing as the energy delivered by an impactor, along with the size, volume, and geometry of the plasma fireball, influences the overall atmospheric effects.

The sink of HCN through impacts on planets can be evaluated as flux based on the reports by Rimmer et al.^69^ Briefly, the surface-averaged sink for an Earth-like exoplanet can be expressed through the experimental decomposition rate, *S*_HCN_ (see Table 3), the energy deposited by an impact *E*_*i*_, and the frequency of impacts ν_*i*_, evenly distributed over the planet’s surface. Combination of these values gives the surface-averaged sink of HCN, Φ_HCN_ (cm^–2^ s^–1^), as shown in eq 8
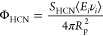
8where *R*_p_ is the radius of the planet. The impact energy and frequency, ⟨*E*_*i*_ν_*i*_⟩, for early Venus, Earth, and Mars during the Late Heavy Bombardment are listed in Table 4.

**Table 4:** Maximum and Minimum HCN Decomposition Fluxes (cm^–2^ s^–1^) for Early Venus, Earth, and Mars Calculated from Our Model^a^

	venus	earth	mars
*R* (km)	6058	6378	3389.5
⟨*E*_*i*_ν_*i*_⟩ (J yr^–^^1^)	9.17 × 10^12^	1.56 × 10^14^	3.98 × 10^13^

	*p*_HCN,i_ (Torr)	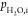 (Torr)	C/O	max. HCN decomposition flux (cm^–^^2^ s^–^^1^)		
max	13	20	0.65	4.52 × 10^12^	1.63 × 10^13^	3.40 × 10^12^
min	0.5	0		2.05 × 10^10^	7.41 × 10^10^	1.54 × 10^10^

a The calculations were performed at 0.1 s of irradiation (1 pulse) for different compositions (indicated).

The values for Early Earth are derived from Rimmer et al.,^69^ whereas those for Venus and Mars have been adjusted by factors of 4 for Earth/Venus and 17 for Earth/Mars, as described in Ferus et al.^70^ Applying eq 8 and using the maximum *S*_HCN_ decomposition rates from our experiments, the appropriate ⟨*E*_*i*_ν_*i*_⟩, and given planetary radii, the maximum surface-average sinks of HCN for early Venus, Earth, and Mars during the LHB are given in Table 4. The calculated maximum HCN sink fluxes for Earth, Venus, and Mars are 1.63 × 10^13^, 4.52 × 10^12^, and 3.40 × 10^12^ cm^–2^ s^–1^, respectively. These were obtained for a model atmosphere containing 13.0 Torr HCN (∼1.7 vol %) and 20.0 Torr H_2_O (2.8 vol %), corresponding to the energy of a single laser pulse (0.1 s, at 10 Hz laser repetition) delivered to the model gas mixture. The minimum HCN decomposition fluxes were determined from the experiment containing 0.5 Torr HCN (∼0.07 vol %) and almost no water (∼0.01 Torr) with values of 7.41 × 10^10^, 2.05 × 10^10^, and 1.54 × 10^10^ cm^–2^ s^–1^ for Earth, Venus, and Mars, respectively. Such results may, in principle, apply to terrestrial (exo)planets of the same sizes and with the same impact histories. These fluxes should be treated as upper limits because, in a real atmosphere, the HCN sinks will be partially countered by production reactions from other atmospheric constituents.

**Figure 8:**
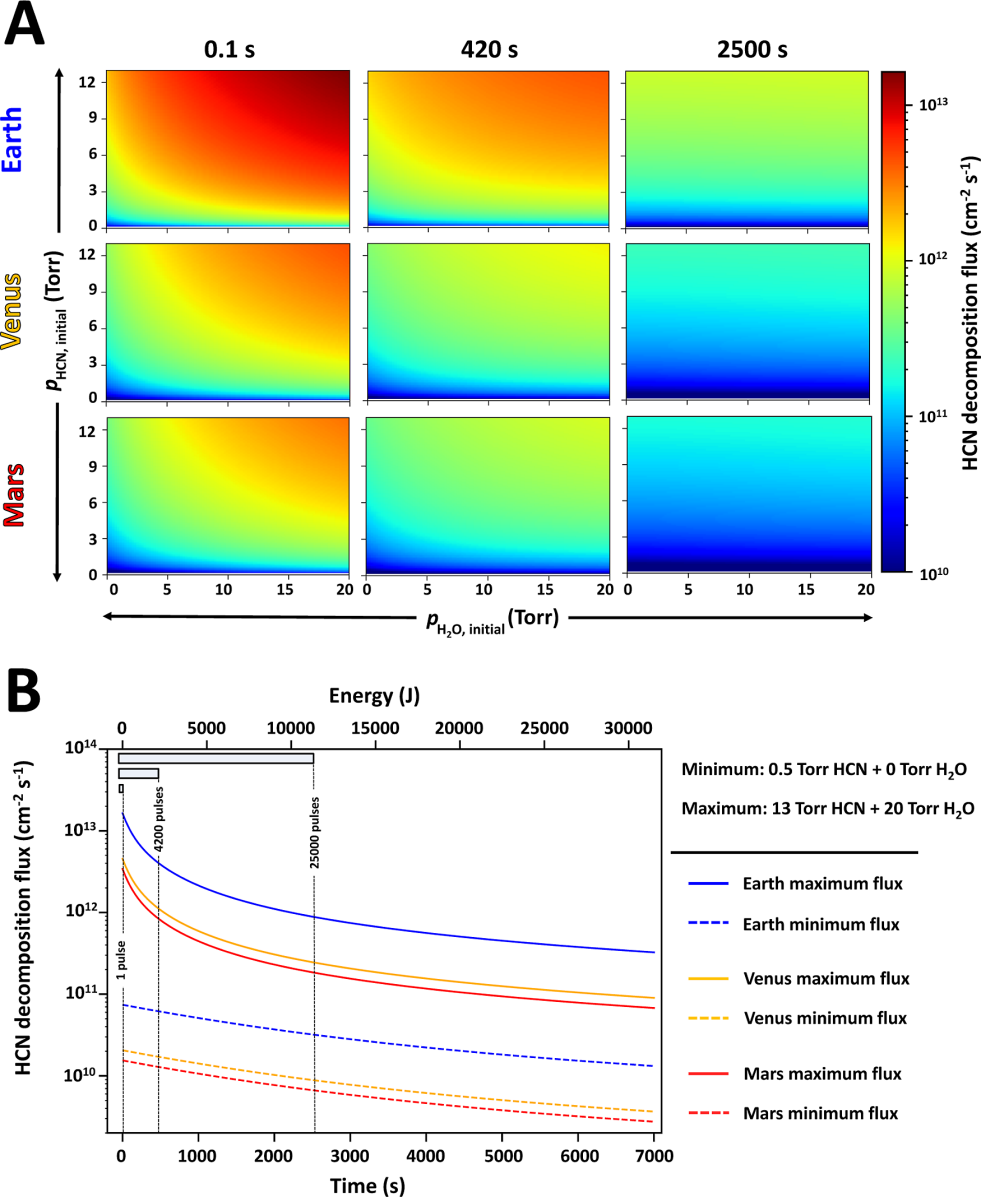
Panel A shows HCN decomposition fluxes (cm^–2^ s^–1^) calculated for the early Earth, Venus, and Mars (rows) at different irradiation times (columns) for a range of initial conditions. Panel B shows maximum and minimum HCN decomposition fluxes for the three planets as a function of irradiation time. Note that the laser frequency is 10 Hz, and therefore, the number of pulses = 10× irradiation time.

At the end, we discuss the importance of specifying the time interval of laser irradiation of the gas mixture for observing the synthesis or/and decomposition of chemical species. Figure 8 shows the calculated HCN sink fluxes (cm^–2^ s^–1^) for the early Earth, Venus, and Mars with different initial compositions at different times.

In the case of the early Earth, our calculated fluxes of HCN decomposition are comparable to those of HCN production from methane published by the reports of Rimmer et al.^69^ Their results were obtained by LIDB-mimicked impact in an ∼1 bar model atmosphere containing an equimolar gaseous mixture of CH_4_, CO, and N_2_. The impact shock was simulated by a high-power laser system with a pulse energy of about 150 J. The 15 pulses they used delivered a total of about 2250 J to their model atmosphere. This would equal to ∼500 s irradiation in our experiment. The experiments are different in terms of the used lasers and delivered energy, but the decomposition and production rates are similar. However, the laser spark volume, geometry, and energy are strikingly different for each experiment.

From our experience, the laser spark has its own “saturation” limit, where at a certain concentration, all the available energy is used to decompose the reactant.^56^ Increasing the amount of available reactant in that stage does not lead to faster decomposition. The reaction then resembles a zeroth-order reaction with a constant reaction rate limited by the amount of available energy. As the reaction progresses and the amount of reactant decreases, the amount of reactant will become the limiting factor, and the reaction will start to follow its appropriate kinetics. The high-concentration constant decomposition rate is visible in our experimental data as the linear part of the decomposition. This is also the region of linear decomposition shown by Scattergood et al.^68^ Further exploration of this phenomenon will be the subject of a follow-up study. With this in mind, the HCN decomposition rates presented in this paper should be interpreted as an upper limit; in a real atmosphere, the sinks will be partially countered by HCN production reactions from other atmospheric constituents.

Last, to compare our results to the atmospheric photochemical production of HCN, Zahnle^30^ and later Tian et al.^29^ show that the production of HCN during Archean (3.8–2.5 Gya) could have been as high as 10^10^ cm^–2^ s^–1^. Alongside with Rimmer and Rugheimer,^38^ these papers argue that HCN may have accumulated on the early Earth depending on the C/O ratio in the planetary atmosphere. The HCN production rate from methane prior to that era, however, was likely much lower (10^6^–10^7^ cm^–2^ s^–1^). Our upper limits of HCN decomposition rates show that during the late heavy bombardment, the decomposition of HCN could have been efficient enough to negate any photochemical production. On the other hand, this could only be concluded assuming that no simultaneous HCN formation channels should exist during impact plasma reprocessing. When other species, such as methane or other carbohydrates, are present in the atmosphere, HCN is likely to form as well.^56,67,69^ Key to further research is thus assessing the rates of impact-initiated HCN formation from methane (as the most significant source of HCN) as well as of simultaneous HCN decomposition in these conditions. We intend to address these questions in a follow-up study.

## 9 Conclusions

An extensive experimental survey was conducted to explore the stability and decomposition of HCN in plasma shocks created by atmospheric entry or the impact of an asteroid or comet. These conditions were simulated using dielectric breakdown induced by a 450 mJ Nd:YAG laser pulses in atmospheres comprising of HCN, H_2_O, and bulk N_2_, resembling those of rocky planets. The experiments demonstrated that HCN decomposes under both wet and dry conditions during these simulated impacts. The main decomposition products in dry, water-poor impacts are solid phase products. These are likely tholins, soot, and refractory carbonaceous material. The presence of water vapor opens up a new reaction channel, and in wet, water-rich atmosphere, the impacts produce CO and CO_2_.

This HCN decomposition process was mapped using a set of the system’s rate coefficients, revealing that the presence of water not only changes the product mixture but also enhances the HCN decomposition rate. A kinetic model constructed using these rate coefficients allows the prediction of the system composition during the irradiation for initial partial pressures ranging from 0 to 13 Torr of HCN and 0 to 20 Torr of H_2_O in a bulk atmosphere of up to 720 Torr of N_2_.

Depending on the initial conditions, our experiments give an initial HCN decomposition rate between 2.43 × 10^15^ and 5.17 × 10^17^ molec J^–1^ of input energy, which is comparable to the HCN synthesis rates reported in the literature. Applying our HCN decomposition rates to the early Earth during LHB, we obtain an upper limit on the HCN decomposition flux between 7.41 × 10^10^ and 1.63 × 10^13^ cm^–2^ s^–1^ depending on the initial composition. This is comparable to the HCN impact synthesis rates published in the literature. Impacts are therefore both an important source as well as sink of atmospheric HCN. We discuss extensively throughout the paper that these are the upper limits of fluxes and rates. However, the decomposition process is not linear, and changing the experimental irradiation time leads to significantly different results. In contrast to the existing studies, we therefore show that the obtained fluxes strongly depend on the nature of the experiment and the chemical process and suggest that the usage rate coefficients and their proper inclusion in kinetic models are necessary instead.

## Data Availability

All data generated during data analysis in this paper deemed overextensive for the on-site Supporting Information are available on an external repository Zenodo^71^ and are referenced throughout Supporting Information to this paper. All original unprocessed data are available upon reasonable request from the corresponding author.

## Supporting Information Available

Irradiation cell for gas phase experiments; irradiation cell for solid phase experiments; normalized relative abundance of HCN and DCN; Y_CO,_ Y_CO2_, and *Y*C2H2 with respect to the initial partial pressures of HCN and H2O; UV-Vis spectrum of the laser induced dielectric breakdown; relative proportion of gas phase products; photograph of the measurement cell; FTIR spectrum of experiment 9; apparatus and synthetic process of HCN; maximum and minimum HCN decomposition fluxes; R^2^-like indicator; model results of the HCN decomposition; HCN decomposition rate; HCN, H_2_O, CO, CO_2_, and CO + CO2 partial pressure; and amount of solid phase carbon partial pressure and gas phase carbon partial pressure (PDF)
