# Supplementary material for: Decomposition of HCN during Experimental Impacts in Dry and Wet Planetary Atmospheres
Source: ACS Earth Space Chem. 2024 May 24;8(6):1246–58. doi: 10.1021/acsearthspacechem.4c00064 (PMC11195306; doi:10.1021/acsearthspacechem.4c00064)
Supplement: Supplementary file 1 — sp4c00064_si_001.pdf [file sp4c00064_si_001.pdf]

Supplementary Material: Decomposition of HCN during Experimental Impacts in Dry and Wet Planetary Atmospheres

Antonín Knížek[1,2], Lukáš Petera[1,3], Vojtěch Laitl[1,4] and Martin Ferus[1]

[1]J. Heyrovský Institute of Physical Chemistry, Czech Academy of Sciences, Dolejškova 2155/3, 18223, Prague, Czech Republic

Email: knizeka@gmail.com, antonin.knizek@jh-inst.cas.cz

[2]Department of Physical and Macromolecular Chemistry, Faculty of Science, Charles University, Hlavova 8, 12800, Prague, Czech Republic

[3]Department of Inorganic Chemistry, Faculty of Science, Charles University, Hlavova 8, 12800, Prague, Czech Republic

[4]University of Antwerp, Faculty of Science, Groenenborgerlaan 171, BE2020 Antwerpen, Belgium

1 Description

This material contains the description of the data analysis process described in the main body of the text.

Various files with outputs from the rate equation fits (machine readable .txt files) as well as visualized outputs of the R^2 values of each fit or covariances of the fitted parameters are all parts of this repository.

The first part of this file contains the description of the data analysis process, as it appears in the main body of the paper, along with several new comments and more broadly discussed points.

The second part of this document constitutes of additional data referenced in the main paper, such as data on the Applicability Range section, etc.

Lists of referenced files are shown in the relevant places in this document. Referenced files along with a copy of this file can be found at **10.5281/zenodo.8086772**.

2 Mechanism and rates

This section is a broadened description of the section *Mechanism and rates* in the main paper.

The main work of this paper was in total nine experiments, each with a different starting composition, but analyzed in the exact same manner. The data obtained from the experiments constitute FTIR spectra of the gas phase products measured at set intervals during the laser irradiation. The spectra were analyzed with the *spectr* library (as described in the main paper) and partial pressures were obtained as the output from the analysis. The time-dependent partial pressures of HCN, H_2O, CO and CO_2 were used to measure the rate of HCN decomposition.

The nine corresponding figures with the partial pressures are shown in the following pdf files:

1. HCN_1.18Torr_N2_H2O_3.4Torr.pdf
2. HCN_1.18Torr_N2_H2O_14.1Torr.pdf
3. HCN_1.25Torr_N2_H2O_17.4Torr.pdf
4. HCN_1.35Torr_N2_H2O_7.1Torr.pdf
5. HCN_1.2Torr_N2_H2O_1.6Torr.pdf
6. HCN_1.3Torr_N2_H2O_0.3Torr.pdf
7. HCN_1.12Torr_N2_H2O_12.4Torr.pdf
8. HCN_1.15Torr_N2_H2O(nika).pdf
9. HCN_1.18Torr_N2_H2O_0.8Torr.pdf

On a side note, the partial pressures used in the file names are partial pressures read from the pressure gauges during filling. It is interesting to see how much error these values have when compared with the values obtained from the spectra.

The system of the main reactions is reasonably well described by the following equations:

$$HCN + H_2O \xleftrightarrow{k_{1,f},k_{1,r}} CO + XX \tag{1}$$

$$CO + H_2O \xleftrightarrow{k_{2,f},k_{2,r}} CO_2 + YY \tag{2}$$

$$HCN \xrightarrow{k_3} [HCN]_n \tag{3}$$

$$H_2O \xrightarrow{k_4} H_2 + O_2 \tag{4}$$

In this set of equations, XX and YY represent unobserved products. Those may be solid phase products as well as gas phase products without dipole moments (impossible to observe with FTIR, such as H_2 or O_2).

Eq. (1) describes the formation of CO. Then, as shown in Eq. (2), CO is further oxidized to CO_2. Aside from these reactions, both HCN and H_2O are themselves decomposed, as observed from the experimental data. We did not identify any products of this process, but the most likely products are H_2 and O_2 from the decomposition of water and solid phase material (tholins, carbon, etc.) from the decomposition of HCN. The formation of solid products was indeed observed during the experiment.

In order to describe the kinetics of the process, the following rate equations were fit to the data:

$$\frac{dp_{HCN}}{dt} = -k_{1,f}p_{HCN}p_{H_2O} + k_{1,r}p_{CO} - k_3p_{HCN} \quad (5)$$

$$\frac{dp_{H_2O}}{dt} = -k_{1,f}p_{HCN}p_{H_2O} + k_{1,r}p_{CO} - k_{2,f}p_{CO}p_{H_2O} + k_{2,r}p_{CO_2} - k_4p_{H_2O} \quad (6)$$

$$\frac{dp_{CO}}{dt} = k_{1,f}p_{HCN}p_{H_2O} - k_{1,r}p_{CO} - k_{2,f}p_{CO}p_{H_2O} + k_{2,r}p_{CO_2} \quad (7)$$

$$\frac{dp_{CO_2}}{dt} = k_{2,f}p_{CO}p_{H_2O} - k_{2,r}p_{CO_2} \quad (8)$$

For the purposes of the fit, we take:

$$p_{HCN} = A_{HCN}p_{HCN} \quad (9)$$

$$p_{H_2O} = A_{H_2O}p_{H_2O} \quad (10)$$

where A_x is a factor which accounts for adsorption.

Adsorption plays a very important role in the experiment. The experiment was constructed to minimize the amount of material that the gases come into contact with and currently, the only surfaces available to the gas are the glass walls of the apparatus, the Teflon fan and rubber o-rings, which are part of Ultra-Torr vacuum fittings (Swagelok®, USA). All present gases adsorb on the surface, which ideally could be experimentally estimated by injecting a known molar amount of gas and comparing the expected partial pressure (from the ideal gas equation) and the observed partial pressure. The issue with this approach is that the adsorption depends on the nature of the specific gas, the available surface, but also temperature, the composition of the rest of the mixture and the formation of solid phase products, which may also selectively enhance adsorption.

During the experiment, when HCN and H_2O decompose in the gas phase, they are supplemented from the adsorbed layers, so when fitting the rate equations, one must consider the total amount of available molecules and not just the amount available in the gas phase. For this reason, two factors, A_x, were employed as multipliers to the observed partial pressures of HCN and H_2O gas (Eqs. (9) and (10)). The value of these multipliers is unknown with the lower bound of 1 (no adsorption) and experimental estimate around 2-3. For the cases of CO and CO_2, the adsorption is in this case negligible, because both HCN and H_2O adsorb much more efficiently. The value of these multipliers is unknown with the lower bound of 1 (no adsorption) and experimental estimate around 2-3.

To get the rate coefficients of the reactions (5), (6), (7) and (8), we fit the constants of the above model (as a set of ordinary differential equations) using the python symfit package with set values of the multipliers A_{HCN} and A_{H2O}. Eight fixed values of each multiplier were chosen (1, 2, 3, 4, 5, 6, 7, 8) for each compound. This means, that each of the nine experiments was fit 64×, with a different pair of multipliers each time. Each of these fits returned the values for six rate coefficients ($k_{1,f}$, $k_{1,r}$, $k_{2,f}$, $k_{2,r}$, k_3 and k_4) along with standard deviations for each value. As a goodness of fit qualifier, we used the R^2 value, which was stored as well. All this data are shown in files:

1. R_sq_HCN_1.2Torr+N2+H2O_1.6Torr.txt
2. R_sq_HCN_1.3Torr+N2+H2O_0.3Torr.txt
3. R_sq_HCN_1.12Torr+N2+H2O_12.4Torr.txt
4. R_sq_HCN_1.15Torr+N2+H2O(nika).txt
5. R_sq_HCN_1.18Torr+N2+H2O_3.4Torr.txt
6. R_sq_HCN_1.18Torr+N2+H2O_0.8Torr.txt

7. R_sq_HCN_1.18Torr+N2+H2O_14.1Torr.txt
8. R_sq_HCN_1.25Torr+N2+H2O_17.4Torr.txt
9. R_sq_HCN_1.35Torr+N2+H2O_7.1Torr.txt

After obtaining these results, we plotted the R^2 vs. the A multipliers for each experiment to see the optimal value of each multiplier where the fit would best represent each experiment's data. The 3D bar charts are shown in the following files:

1. R_sq_HCN_1.2Torr+N2+H2O_1.6Torr.pdf
2. R_sq_HCN_1.3Torr+N2+H2O_0.3Torr.pdf
3. R_sq_HCN_1.12Torr+N2+H2O_12.4Torr.pdf
4. R_sq_HCN_1.15Torr+N2+H2O(nika).pdf
5. R_sq_HCN_1.18Torr+N2+H2O_3.4Torr.pdf
6. R_sq_HCN_1.18Torr+N2+H2O_0.8Torr.pdf
7. R_sq_HCN_1.18Torr+N2+H2O_14.1Torr.pdf
8. R_sq_HCN_1.25Torr+N2+H2O_17.4Torr.pdf
9. R_sq_HCN_1.35Torr+N2+H2O_7.1Torr.pdf

However, comparison of the R^2 of each fit did not show any optimal values of A_{HCN} and A_{H2O}. Rather, the fit behaves so that when the multipliers increase from 1, the R^2 increases, and the fit improves up to a certain R^2 value. This is because at low (close to 1) values of the multipliers, there is not enough carbon containing compounds in the data to even explain the formation of CO and CO_2. In fact, due to adsorption, the sum of the partial pressures of C-containing compounds is for most experiments less than the sum of partial pressures of products formed in the experiment. After escaping this problem by increasing the multipliers, any further increase is compensated by the increase in the rate coefficients (often $k_{1,f}$ and $k_{1,r}$ for experiments with lower initial amounts of water, and k_3 and k_4 for experiments with higher amounts of water). Therefore, the R^2 is a good indicator for incorrectly low multipliers but becomes ambiguous for their higher values.

Then, upon assumption that the values of rate coefficients $k_{1,f}$, $k_{1,r}$, $k_{2,f}$ and $k_{2,r}$ converge to the correct value, the mean value of R^2 and its standard deviation were thus calculated for each experiment. Combinations of multiplier pairs whose R^2 value differed from the mean by more than double the standard deviation of the mean of R^2 were discarded. This effectively separated out the incorrectly low values of multipliers. The fit results after discarding those values are shown in files:

1. R_sq_HCN_1.2Torr+N2+H2O_1.6Torr_reduced.txt
2. R_sq_HCN_1.3Torr+N2+H2O_0.3Torr_reduced.txt
3. R_sq_HCN_1.12Torr+N2+H2O_12.4Torr_reduced.txt
4. R_sq_HCN_1.15Torr+N2+H2O(nika)_reduced.txt
5. R_sq_HCN_1.18Torr+N2+H2O_3.4Torr_reduced.txt
6. R_sq_HCN_1.18Torr+N2+H2O_0.8Torr_reduced.txt
7. R_sq_HCN_1.18Torr+N2+H2O_14.1Torr_reduced.txt
8. R_sq_HCN_1.25Torr+N2+H2O_17.4Torr_reduced.txt
9. R_sq_HCN_1.35Torr+N2+H2O_7.1Torr_reduced.txt

Visualization was also performed, and the 3D bar plots are shown in files:

1. R_sq_HCN_1.2Torr+N2+H2O_1.6Torr_reduced.pdf

2. R_sq_HCN_1.3Torr+N2+H2O_0.3Torr_reduced. pdf
3. R_sq_HCN_1.12Torr+N2+H2O_12.4Torr_reduced. pdf
4. R_sq_HCN_1.15Torr+N2+H2O(nika)_reduced. pdf
5. R_sq_HCN_1.18Torr+N2+H2O_3.4Torr_reduced. pdf
6. R_sq_HCN_1.18Torr+N2+H2O_0.8Torr_reduced. pdf
7. R_sq_HCN_1.18Torr+N2+H2O_14.1Torr_reduced. pdf
8. R_sq_HCN_1.25Torr+N2+H2O_17.4Torr_reduced. pdf
9. R_sq_HCN_1.35Torr+N2+H2O_7.1Torr_reduced. pdf

Subsequently, for each experiment, means of each coefficient were calculated from the remaining calculations, along with standard deviations and covariances of the coefficients and the values of each multiplier (i.e. for each coefficient k_x, we calculated $cov_{k_x,A_{HCN}}$ and $cov_{k_x,A_{H_2O}}$). The covariances were always <1×10^{-2} and mostly <1×10^{-3}, showing little to no correlation to the multipliers. The means for each coefficient, the standard deviations of the means and the covariances are shown in files:

1. res_fin_HCN_1.2Torr+N2+H2O_1.6Torr.txt
2. res_fin _HCN_1.3Torr+N2+H2O_0.3Torr.txt
3. res_fin _HCN_1.12Torr+N2+H2O_12.4Torr.txt
4. res_fin _HCN_1.15Torr+N2+H2O(nika).txt
5. res_fin _HCN_1.18Torr+N2+H2O_3.4Torr.txt
6. res_fin _HCN_1.18Torr+N2+H2O_0.8Torr.txt
7. res_fin _HCN_1.18Torr+N2+H2O_14.1Torr.txt
8. res_fin _HCN_1.25Torr+N2+H2O_17.4Torr.txt
9. res_fin _HCN_1.35Torr+N2+H2O_7.1Torr.txt

To see if the covariances are indeed small and the obtained rate coefficients are independent of the multiplier value, we plotted the data, too. The plots for some experiments show non-negligible covariances for some parameters, but never across all experiments. It therefore does not invalidate the results in any way. The plots of each coefficient against both multipliers in each experiment are shown in files:

1. R_sq_HCN_1.2Torr+N2+H2O_1.6Torr_k1_f_vs_h2o_multiplier.pdf
2. R_sq_HCN_1.2Torr+N2+H2O_1.6Torr_k1_f_vs_hcn_multiplier.pdf
3. R_sq_HCN_1.2Torr+N2+H2O_1.6Torr_k1_r_vs_h2o_multiplier.pdf
4. R_sq_HCN_1.2Torr+N2+H2O_1.6Torr_k1_r_vs_hcn_multiplier.pdf
5. R_sq_HCN_1.2Torr+N2+H2O_1.6Torr_k2_f_vs_h2o_multiplier.pdf
6. R_sq_HCN_1.2Torr+N2+H2O_1.6Torr_k2_f_vs_hcn_multiplier.pdf
7. R_sq_HCN_1.2Torr+N2+H2O_1.6Torr_k2_r_vs_h2o_multiplier.pdf
8. R_sq_HCN_1.2Torr+N2+H2O_1.6Torr_k2_r_vs_hcn_multiplier.pdf
9. R_sq_HCN_1.2Torr+N2+H2O_1.6Torr_k3_vs_h2o_multiplier.pdf
10. R_sq_HCN_1.2Torr+N2+H2O_1.6Torr_k3_vs_hcn_multiplier.pdf
11. R_sq_HCN_1.2Torr+N2+H2O_1.6Torr_k4_vs_h2o_multiplier.pdf
12. R_sq_HCN_1.2Torr+N2+H2O_1.6Torr_k4_vs_hcn_multiplier.pdf
13. R_sq_HCN_1.3Torr+N2+H2O_0.3Torr_k1_f_vs_h2o_multiplier.pdf
14. R_sq_HCN_1.3Torr+N2+H2O_0.3Torr_k1_f_vs_hcn_multiplier.pdf
15. R_sq_HCN_1.3Torr+N2+H2O_0.3Torr_k1_r_vs_h2o_multiplier.pdf
16. R_sq_HCN_1.3Torr+N2+H2O_0.3Torr_k1_r_vs_hcn_multiplier.pdf
17. R_sq_HCN_1.3Torr+N2+H2O_0.3Torr_k2_f_vs_h2o_multiplier.pdf
18. R_sq_HCN_1.3Torr+N2+H2O_0.3Torr_k2_f_vs_hcn_multiplier.pdf
19. R_sq_HCN_1.3Torr+N2+H2O_0.3Torr_k2_r_vs_h2o_multiplier.pdf

20. R_sq_HCN_1.3Torr+N2+H2O_0.3Torr_k2_r_vs_hcn_multiplier.pdf
21. R_sq_HCN_1.3Torr+N2+H2O_0.3Torr_k3_vs_h2o_multiplier.pdf
22. R_sq_HCN_1.3Torr+N2+H2O_0.3Torr_k3_vs_hcn_multiplier.pdf
23. R_sq_HCN_1.3Torr+N2+H2O_0.3Torr_k4_vs_h2o_multiplier.pdf
24. R_sq_HCN_1.3Torr+N2+H2O_0.3Torr_k4_vs_hcn_multiplier.pdf
25. R_sq_HCN_1.12Torr+N2+H2O_12.4Torr_k1_f_vs_h2o_multiplier.pdf
26. R_sq_HCN_1.12Torr+N2+H2O_12.4Torr_k1_f_vs_hcn_multiplier.pdf
27. R_sq_HCN_1.12Torr+N2+H2O_12.4Torr_k1_r_vs_h2o_multiplier.pdf
28. R_sq_HCN_1.12Torr+N2+H2O_12.4Torr_k1_r_vs_hcn_multiplier.pdf
29. R_sq_HCN_1.12Torr+N2+H2O_12.4Torr_k2_f_vs_h2o_multiplier.pdf
30. R_sq_HCN_1.12Torr+N2+H2O_12.4Torr_k2_f_vs_hcn_multiplier.pdf
31. R_sq_HCN_1.12Torr+N2+H2O_12.4Torr_k2_r_vs_h2o_multiplier.pdf
32. R_sq_HCN_1.12Torr+N2+H2O_12.4Torr_k2_r_vs_hcn_multiplier.pdf
33. R_sq_HCN_1.12Torr+N2+H2O_12.4Torr_k3_vs_h2o_multiplier.pdf
34. R_sq_HCN_1.12Torr+N2+H2O_12.4Torr_k3_vs_hcn_multiplier.pdf
35. R_sq_HCN_1.12Torr+N2+H2O_12.4Torr_k4_vs_h2o_multiplier.pdf
36. R_sq_HCN_1.12Torr+N2+H2O_12.4Torr_k4_vs_hcn_multiplier.pdf
37. R_sq_HCN_1.15Torr+N2+H2O(nika)_k1_f_vs_h2o_multiplier.pdf
38. R_sq_HCN_1.15Torr+N2+H2O(nika)_k1_f_vs_hcn_multiplier.pdf
39. R_sq_HCN_1.15Torr+N2+H2O(nika)_k1_r_vs_h2o_multiplier.pdf
40. R_sq_HCN_1.15Torr+N2+H2O(nika)_k1_r_vs_hcn_multiplier.pdf
41. R_sq_HCN_1.15Torr+N2+H2O(nika)_k2_f_vs_h2o_multiplier.pdf
42. R_sq_HCN_1.15Torr+N2+H2O(nika)_k2_f_vs_hcn_multiplier.pdf
43. R_sq_HCN_1.15Torr+N2+H2O(nika)_k2_r_vs_h2o_multiplier.pdf
44. R_sq_HCN_1.15Torr+N2+H2O(nika)_k2_r_vs_hcn_multiplier.pdf
45. R_sq_HCN_1.15Torr+N2+H2O(nika)_k3_vs_h2o_multiplier.pdf
46. R_sq_HCN_1.15Torr+N2+H2O(nika)_k3_vs_hcn_multiplier.pdf
47. R_sq_HCN_1.15Torr+N2+H2O(nika)_k4_vs_h2o_multiplier.pdf
48. R_sq_HCN_1.15Torr+N2+H2O(nika)_k4_vs_hcn_multiplier.pdf
49. R_sq_HCN_1.18Torr+N2+H2O_3.4Torr_k1_f_vs_h2o_multiplier.pdf
50. R_sq_HCN_1.18Torr+N2+H2O_3.4Torr_k1_f_vs_hcn_multiplier.pdf
51. R_sq_HCN_1.18Torr+N2+H2O_3.4Torr_k1_r_vs_h2o_multiplier.pdf
52. R_sq_HCN_1.18Torr+N2+H2O_3.4Torr_k1_r_vs_hcn_multiplier.pdf
53. R_sq_HCN_1.18Torr+N2+H2O_3.4Torr_k2_f_vs_h2o_multiplier.pdf
54. R_sq_HCN_1.18Torr+N2+H2O_3.4Torr_k2_f_vs_hcn_multiplier.pdf
55. R_sq_HCN_1.18Torr+N2+H2O_3.4Torr_k2_r_vs_h2o_multiplier.pdf
56. R_sq_HCN_1.18Torr+N2+H2O_3.4Torr_k2_r_vs_hcn_multiplier.pdf
57. R_sq_HCN_1.18Torr+N2+H2O_3.4Torr_k3_vs_h2o_multiplier.pdf
58. R_sq_HCN_1.18Torr+N2+H2O_3.4Torr_k3_vs_hcn_multiplier.pdf
59. R_sq_HCN_1.18Torr+N2+H2O_3.4Torr_k4_vs_h2o_multiplier.pdf
60. R_sq_HCN_1.18Torr+N2+H2O_3.4Torr_k4_vs_hcn_multiplier.pdf
61. R_sq_HCN_1.18Torr+N2+H2O_0.8Torr_k1_f_vs_h2o_multiplier.pdf
62. R_sq_HCN_1.18Torr+N2+H2O_0.8Torr_k1_f_vs_hcn_multiplier.pdf
63. R_sq_HCN_1.18Torr+N2+H2O_0.8Torr_k1_r_vs_h2o_multiplier.pdf
64. R_sq_HCN_1.18Torr+N2+H2O_0.8Torr_k1_r_vs_hcn_multiplier.pdf
65. R_sq_HCN_1.18Torr+N2+H2O_0.8Torr_k2_f_vs_h2o_multiplier.pdf
66. R_sq_HCN_1.18Torr+N2+H2O_0.8Torr_k2_f_vs_hcn_multiplier.pdf
67. R_sq_HCN_1.18Torr+N2+H2O_0.8Torr_k2_r_vs_h2o_multiplier.pdf

68. R_sq_HCN_1.18Torr+N2+H2O_0.8Torr_k2_r_vs_hcn_multiplier.pdf
69. R_sq_HCN_1.18Torr+N2+H2O_0.8Torr_k3_vs_h2o_multiplier.pdf
70. R_sq_HCN_1.18Torr+N2+H2O_0.8Torr_k3_vs_hcn_multiplier.pdf
71. R_sq_HCN_1.18Torr+N2+H2O_0.8Torr_k4_vs_h2o_multiplier.pdf
72. R_sq_HCN_1.18Torr+N2+H2O_0.8Torr_k4_vs_hcn_multiplier.pdf
73. R_sq_HCN_1.18Torr+N2+H2O_14.1Torr_k1_f_vs_h2o_multiplier.pdf
74. R_sq_HCN_1.18Torr+N2+H2O_14.1Torr_k1_f_vs_hcn_multiplier.pdf
75. R_sq_HCN_1.18Torr+N2+H2O_vTorr_k1_r_vs_h2o_multiplier.pdf
76. R_sq_HCN_1.18Torr+N2+H2O_14.1Torr_k1_r_vs_hcn_multiplier.pdf
77. R_sq_HCN_1.18Torr+N2+H2O_14.1Torr_k2_f_vs_h2o_multiplier.pdf
78. R_sq_HCN_1.18Torr+N2+H2O_14.1Torr_k2_f_vs_hcn_multiplier.pdf
79. R_sq_HCN_1.18Torr+N2+H2O_14.1Torr_k2_r_vs_h2o_multiplier.pdf
80. R_sq_HCN_1.18Torr+N2+H2O_14.1Torr_k2_r_vs_hcn_multiplier.pdf
81. R_sq_HCN_1.18Torr+N2+H2O_14.1Torr_k3_vs_h2o_multiplier.pdf
82. R_sq_HCN_1.18Torr+N2+H2O_14.1Torr_k3_vs_hcn_multiplier.pdf
83. R_sq_HCN_1.18Torr+N2+H2O_14.1Torr_k4_vs_h2o_multiplier.pdf
84. R_sq_HCN_1.18Torr+N2+H2O_14.1Torr_k4_vs_hcn_multiplier.pdf
85. R_sq_HCN_1.25Torr+N2+H2O_17.4Torr_k1_f_vs_h2o_multiplier.pdf
86. R_sq_HCN_1.25Torr+N2+H2O_17.4Torr_kf_r_vs_hcn_multiplier.pdf
87. R_sq_HCN_1.25Torr+N2+H2O_17.4Torr_k1_r_vs_h2o_multiplier.pdf
88. R_sq_HCN_1.25Torr+N2+H2O_17.4Torr_k1_r_vs_hcn_multiplier.pdf
89. R_sq_HCN_1.25Torr+N2+H2O_17.4Torr_k2_f_vs_h2o_multiplier.pdf
90. R_sq_HCN_1.25Torr+N2+H2O_17.4Torr_k2_f_vs_hcn_multiplier.pdf
91. R_sq_HCN_1.25Torr+N2+H2O_17.4Torr_k2_r_vs_h2o_multiplier.pdf
92. R_sq_HCN_1.25Torr+N2+H2O_17.4Torr_k2_r_vs_hcn_multiplier.pdf
93. R_sq_HCN_1.25Torr+N2+H2O_17.4Torr_k3_vs_h2o_multiplier.pdf
94. R_sq_HCN_1.25Torr+N2+H2O_17.4Torr_k3_vs_hcn_multiplier.pdf
95. R_sq_HCN_1.25Torr+N2+H2O_17.4Torr_k4_vs_h2o_multiplier.pdf
96. R_sq_HCN_1.25Torr+N2+H2O_17.4Torr_k4_vs_hcn_multiplier.pdf
97. R_sq_HCN_1.35Torr+N2+H2O_7.1Torr_k1_f_vs_h2o_multiplier.pdf
98. R_sq_HCN_1.35Torr+N2+H2O_7.1Torr_kf_r_vs_hcn_multiplier.pdf
99. R_sq_HCN_1.35Torr+N2+H2O_7.1Torr_k1_r_vs_h2o_multiplier.pdf
100. R_sq_HCN_1.35Torr+N2+H2O_7.1Torr_k1_r_vs_hcn_multiplier.pdf
101. R_sq_HCN_1.35Torr+N2+H2O_7.1Torr_k2_f_vs_h2o_multiplier.pdf
102. R_sq_HCN_1.35Torr+N2+H2O_7.1Torr_k2_f_vs_hcn_multiplier.pdf
103. R_sq_HCN_1.35Torr+N2+H2O_7.1Torr_k2_r_vs_h2o_multiplier.pdf
104. R_sq_HCN_1.35Torr+N2+H2O_7.1Torr_k2_r_vs_hcn_multiplier.pdf
105. R_sq_HCN_1.35Torr+N2+H2O_7.1Torr_k3_vs_h2o_multiplier.pdf
106. R_sq_HCN_1.35Torr+N2+H2O_7.1Torr_k3_vs_hcn_multiplier.pdf
107. R_sq_HCN_1.35Torr+N2+H2O_7.1Torr_k4_vs_h2o_multiplier.pdf
108. R_sq_HCN_1.35Torr+N2+H2O_7.1Torr_k4_vs_hcn_multiplier.pdf

After calculating the covariances and verifying the calculation in this way, we calculated the mean of each coefficient across the nine experiments along with the standard deviations of the mean.

The final rate coefficients are shown in Table S1 below (identical to Table 3 in the main paper).

Table S1: Rate coefficients obtained from the fit of all our data. This is a copy of Table 3 in the main paper.

Rate constant	Value
$k_{1,f}$	$2.6\times10^{-4} \pm 2.4\times10^{-4}$ Torr s^{-1}
$k_{1,r}$	$6.4\times10^{-4} \pm 3.6\times10^{-4}$ Torr s^{-1}
$k_{2,f}$	$1.2\times10^{-3} \pm 3.8\times10^{-4}$ Torr s^{-1}
$k_{2,r}$	$4.9\times10^{-3} \pm 9.9\times10^{-4}$ Torr s^{-1}
k_3	$8.1\times10^{-4} \pm 6.0\times10^{-4}$ Torr s^{-1}
k_4	$2.4\times10^{-4} \pm 5.5\times10^{-5}$ Torr s^{-1}

As discussed above, it is possible that a weak dependence on the input parameters exists, but since we used a wide range of parameters (the minimum value of the multipliers is given, and we set the maximum as ~3× the value estimated from the experiment) and obtained reasonably small standard deviations and covariances, we do not expect such would-be dependencies to alter our final results. One possible source of error is that the exact radiative transfer model and mechanism in the plasma are not known. It is possible that other minor reaction channels or bottleneck reactions could be present, whose incorporating would help lower the standard deviation. Another possible source of error could be the fact that the multipliers are constant values. Both HCN and H_2O adsorb on the walls of the apparatus and likely compete for the adsorption sites. Change of the amount of reactants in the apparatus during the experiment likely causes a change in the adsorption coefficient/multiplier. Better description of the adsorption mechanism and accounting for the adsorption in the process could also help with reducing the standard deviation values.

The rate coefficients obtained from this fit can be directly implemented into planetary atmospheric models. A possible way to treat the standard deviations is to include them in the planetary atmospheric model or to perform sensitivity analysis within the model.

3 Applicability range

This section contains additional information and lists of files for the Applicability range section of the main paper.

The section in the main paper discusses that a range of experiments with additional initial partial pressures of HCN and H_2O was performed. For each of these experiments, we measured the composition of the gas phase by FTIR (in the same way as with the other experiments) and fit the spectra to obtain partial pressures of all the four principal components of the experiment: HCN, H_2O, CO and CO_2. We then applied the final rate coefficients (Table 3 in the main paper) to the data and using the conditions at 0 s (prior to laser irradiation), attempted to predict the result at 420 s. This was compared to the actual measured result after 420 s of irradiation. The plots which show this comparison for each experiment are contained in files:

1. HCN(0.5Torr)+N2+H2O(0.5Torr).pdf
2. HCN(0.5Torr)+N2+H2O(1.4Torr).pdf
3. HCN(0.5Torr)+N2+H2O(6.2Torr).pdf
4. HCN(0.5Torr)+N2+H2O(21.3Torr).pdf
5. HCN(0.5Torr)+N2+H2O(nika).pdf

6. HCN(1.5Torr)+N2+H2O(0.5Torr).pdf
7. HCN(1.5Torr)+N2+H2O(1.4Torr).pdf
8. HCN(1.5Torr)+N2+H2O(6.0Torr).pdf
9. HCN(1.5Torr)+N2+H2O(12.9Torr).pdf
10. HCN(1.5Torr)+N2+H2O(21Torr).pdf
11. HCN(1.5Torr)+N2+H2O(nika).pdf
12. HCN(3.5Torr)+N2+H2O(17.5Torr).pdf
13. HCN(3.6Torr)+N2+H2O(1.5Torr).pdf
14. HCN(3.6Torr)+N2+H2O(3.6Torr).pdf
15. HCN(3.6Torr)+N2+H2O(7.6Torr).pdf
16. HCN(3.6Torr)+N2+H2O(nika).pdf
17. HCN(7.3Torr)+N2+H2O(8.3Torr).pdf
18. HCN(7.3Torr)+N2+H2O(15.0Torr).pdf
19. HCN(7.3Torr)+N2+H2O(15.6Torr).pdf
20. HCN(7.3Torr)+N2+H2O(nika).pdf
21. HCN(14.2Torr)+N2+H2O(3.0Torr).pdf
22. HCN(14.2Torr)+N2+H2O(9.8Torr).pdf
23. HCN(14.2Torr)+N2+H2O(nikaTorr).pdf

After that, as described in the main paper, the R^2-like indicator of the goodness of fit was calculated. The value was then plotted for each molecule separately (across all experiments). This is shown in files:

1. R_sq_hcn.pdf
2. R_sq_h2o.pdf
3. R_sq_co.pdf
4. R_sq_co2.pdf

The values for the indicator were then averaged to show the overall goodness of fit. The averaged result and the relevant discussion are given in the main paper and are not shown here.

4 Measurement cells

Below, we show schematic drawings of the two irradiation cells which are part of the ELISE apparatus. The cells are interchangeable. Bot cells are made of borosilicate glass.

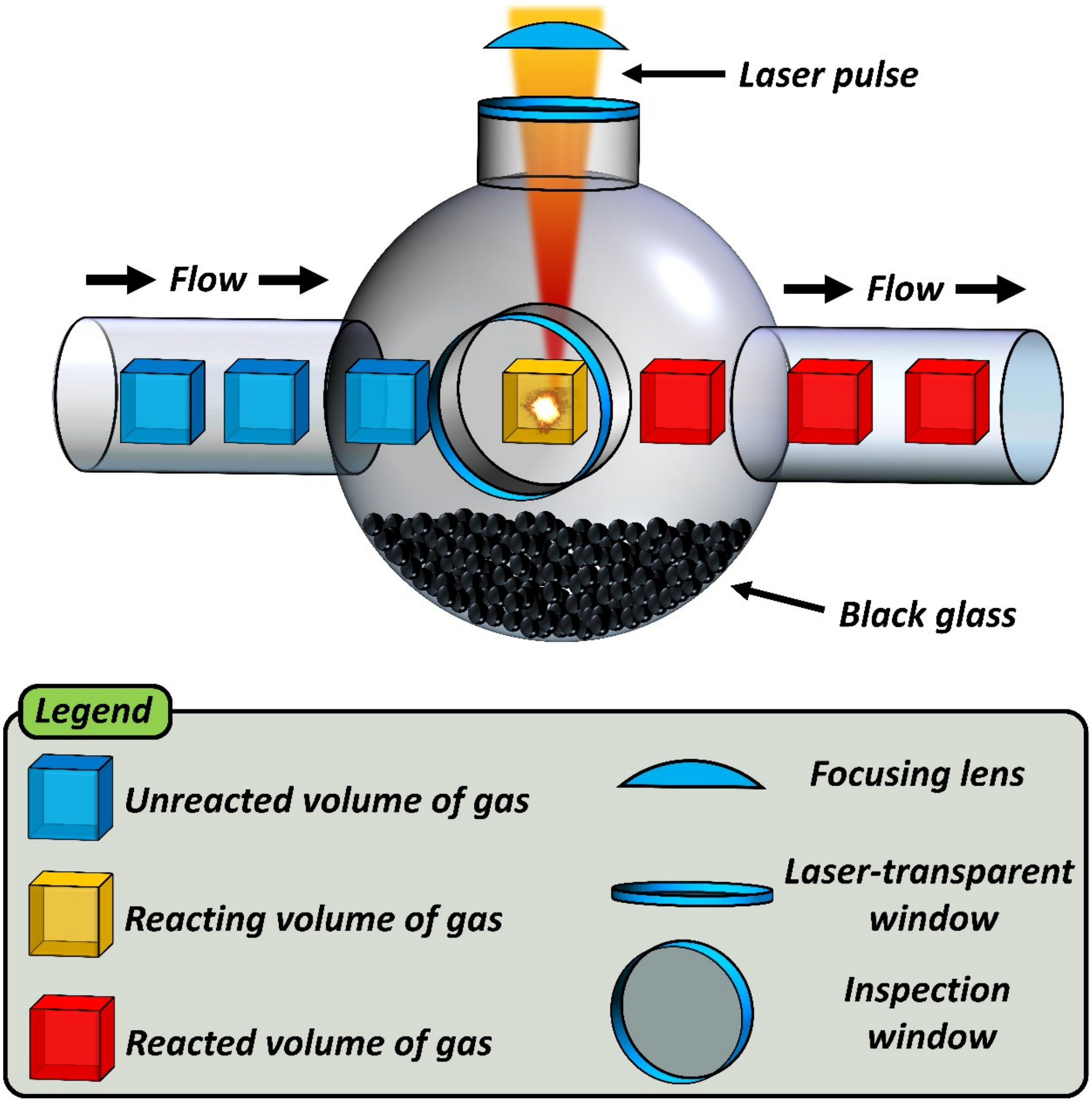

Figure S1: Irradiation cell for gas phase experiments.

The irradiation cell for gas phase experiments is roughly spherical in shape with four 1-inch-in-diameter cylindrical tubes welded to it. The cylinders are used for inlet and outlet of gas, entrance of the laser radiation and UV-Vis inspection window. Glass beads were placed on the bottom of the cell to scatter the passing radiation (otherwise, the bottom of the cell would be damaged by the laser beam).

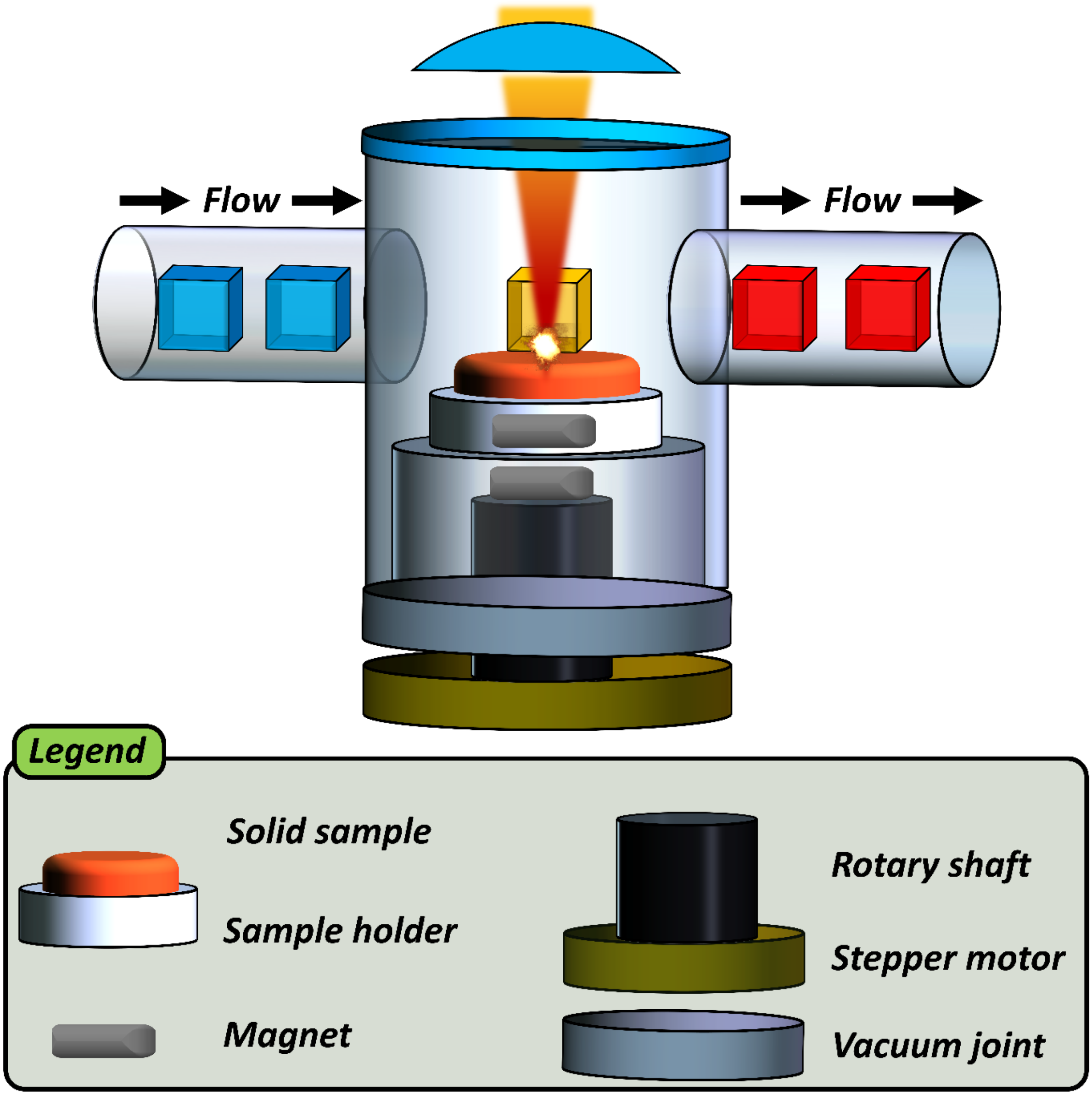

Figure S2: Irradiation cell for solid phase experiments.

The irradiation cell for solid phase experiments is cylindrical. The upper part is similar in design to the cell for the gas phase irradiation. The lower part contains a sample holder on which the solid sample can be placed. Underneath is a rotary shaft with a stepper motor nested in a glass tube. The glass tube is connected to the cell with a vacuum-tight joint. Both parts (the shaft and the sample holder) contain a magnet. The rotation of the stepper motor therefore rotates the sample and ensures that the laser does not ablate a single point in the sample. This is important a) because if the sample is inhomogeneous, ablation of more spots provides a more average result, and b) if the laser fired in one spot only, the sample would quickly be ablated away, and a hole would be created.

5 DCN and the reverse reaction

To prove the fact that the reaction of HCN decomposition is reversible, we performed experiments with D_2 as the source of deuterium and observed the formation of DCN in our experiment. A FTIR spectrum showing the ν_1 (2360 cm^{-1}) band of DCN is shown in the main paper. Here in the Supplementary

Information, we show the relative abundances of HCN and DCN in the experiment. Figure S3 shows the relative abundance of HCN and Figure S4 shows the relative abundance of DCN.

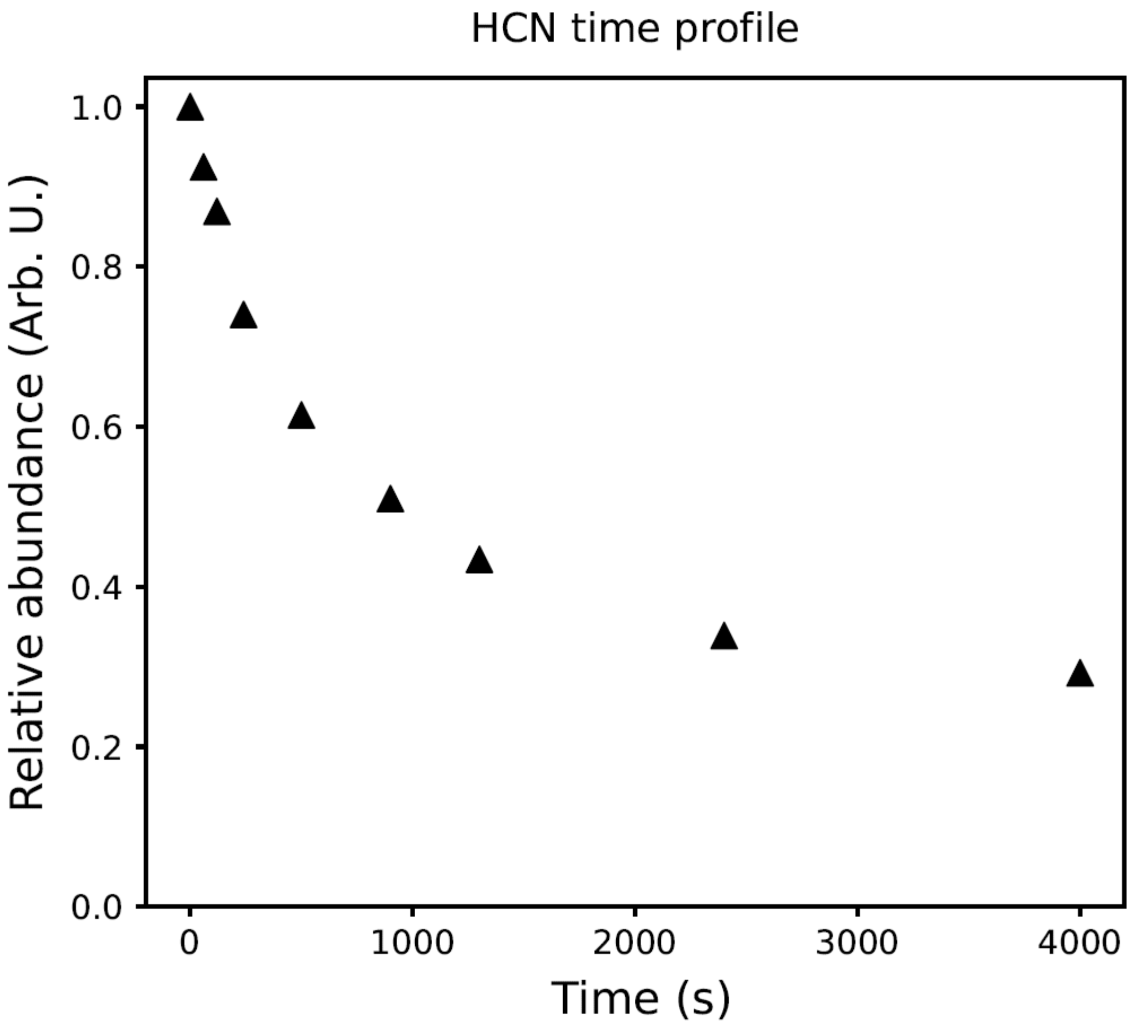

Figure S3: The normalized relative abundance of HCN in the experiment.

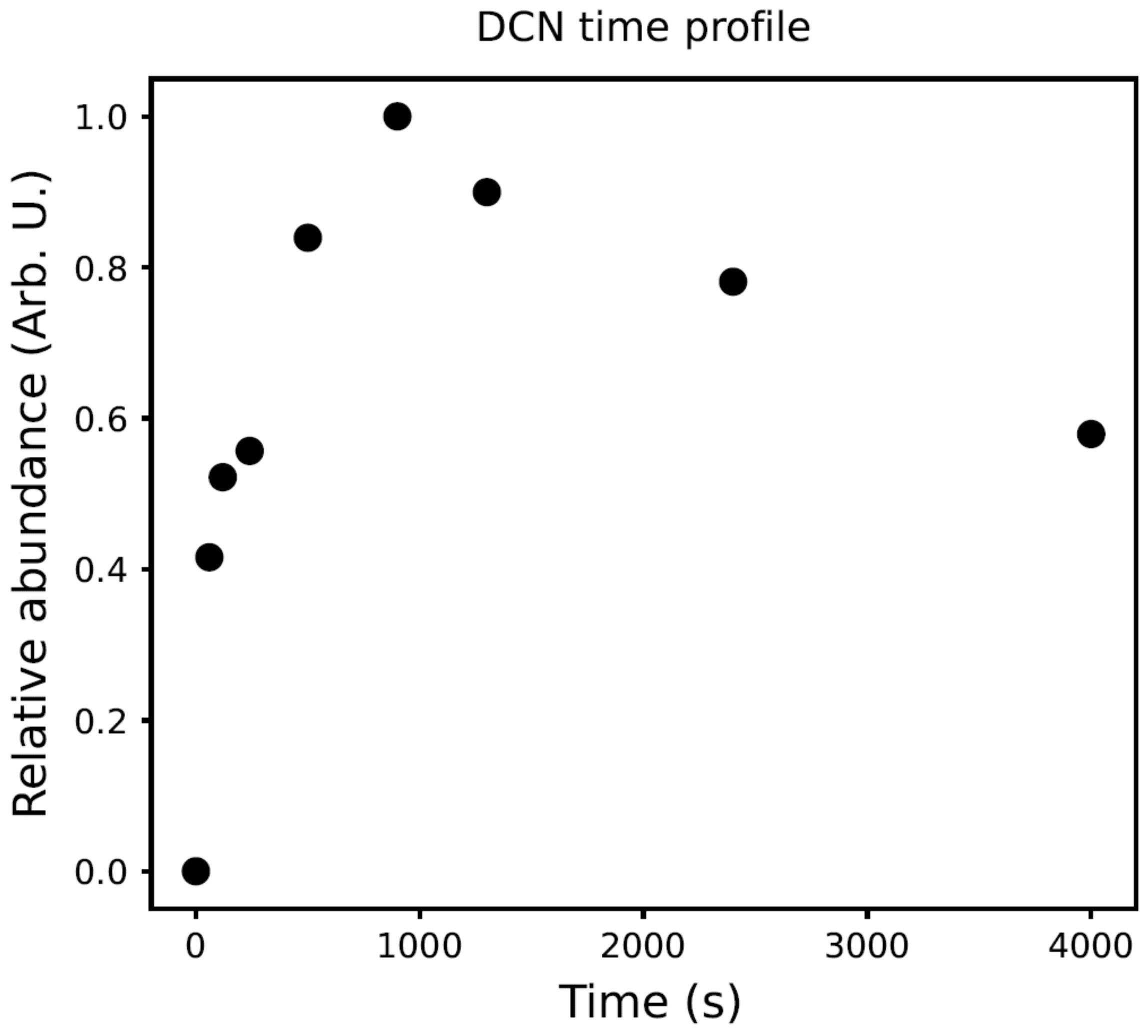

Figure S4: The normalized relative abundance of DCN in the experiment.

The experiment at the beginning contained HCN and D_2. During the irradiation, the HCN is decomposed. In the first step, HCN presumably decomposes to H· and ·CN. At the same time, D_2 decomposes to two D· radicals. The D· and ·CN radicals may then react during the afterglow of the laser-generated plasma to form DCN. Figure S3 shows the relative decrease in the partial pressure of HCN during the experiment. Figure S4 then shows the formation of DCN. Data for both figures were normalized and show relative changes in the abundances. Interestingly, there is a moment when DCN abundance reaches a maximum value and then decreases. At the start, the abundance of DCN increases as a lot of HCN is decomposed and no DCN is present. After some time, however, there is already enough DCN and small enough amount of HCN that the decomposition of DCN begins to dominate over its formation from HCN. The DCN FTIR spectrum in the main paper is the spectrum at the time of the maximum abundance of DCN in the sample.

6 Composition of the gas phase

In this section, we show some visualizations of the gas phase composition after 420 s of irradiation. The following plots were created from the experimental data and not the model. For each of the plots, we define Y_i, which is the relative amount of carbon locked in a species i in the gas phase relative to other species in the gas phase. Y_i is defined as:

$$Y_i = \frac{p_i n_i}{\sum_i p_i n_i} \quad (11)$$

where p_i is the partial pressure of specie *i* and n_i is the number of carbon atoms in the species.

Figure S5 shows Y_{CO} with respect to the initial partial pressures of HCN and H_2O.

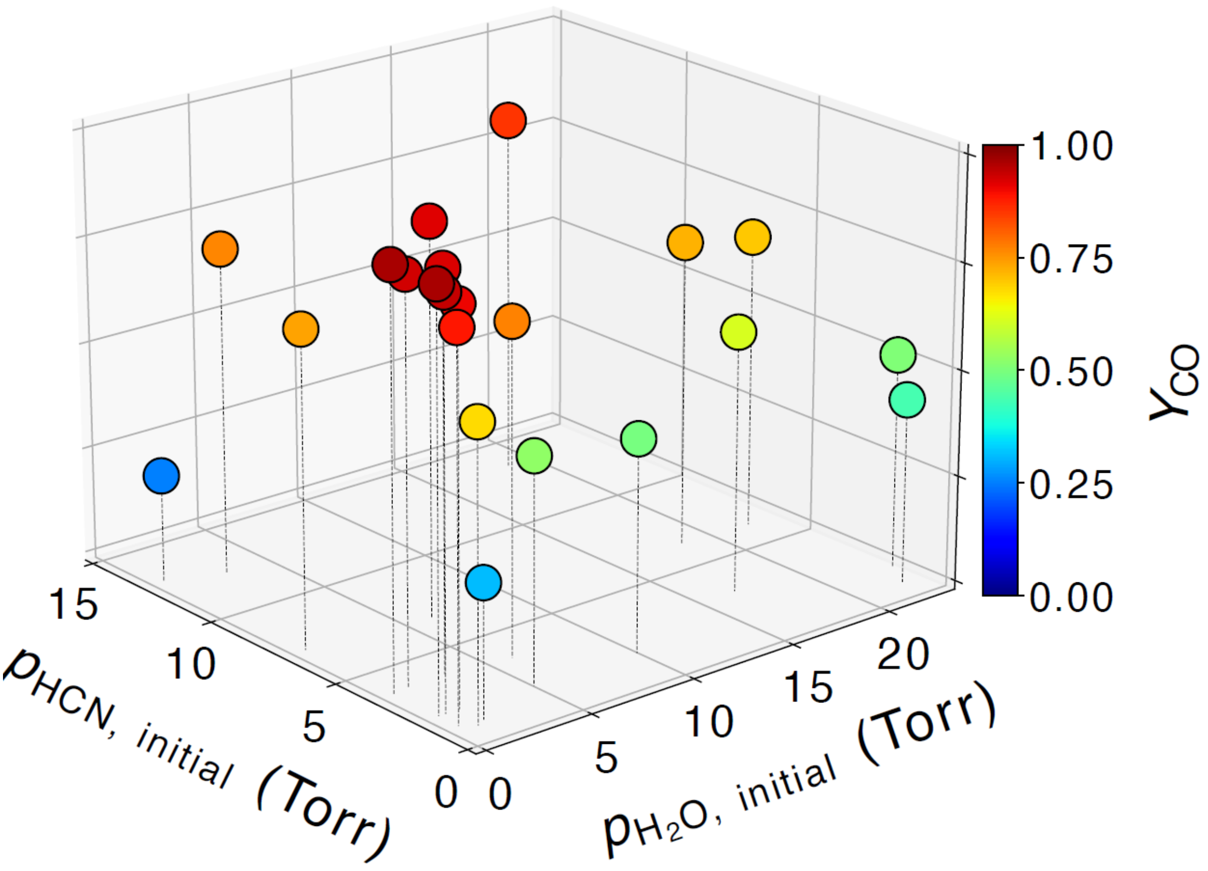

Figure S5: Y_{CO} with respect to the initial partial pressures of HCN and H_2O.

This figure shows that the gas phase in experiments with roughly equal amounts of HCN and H_2O is the highest. Higher initial amounts of H_2O relative to HCN lead to faster consumption of HCN and formation of CO and subsequently CO_2. After 420 s of irradiation, then, the relative amount of CO in the gas phase decreases for experiments with high $p_{H2O, ini}$/ $p_{HCN, ini}$. At the same time, experiments with high initial content of HCN and little to no H_2O do not contain much CO either, mostly because there is little water for its production in the first place.

Figure S6 shows the same property, the Y_{CO2} for CO_2.

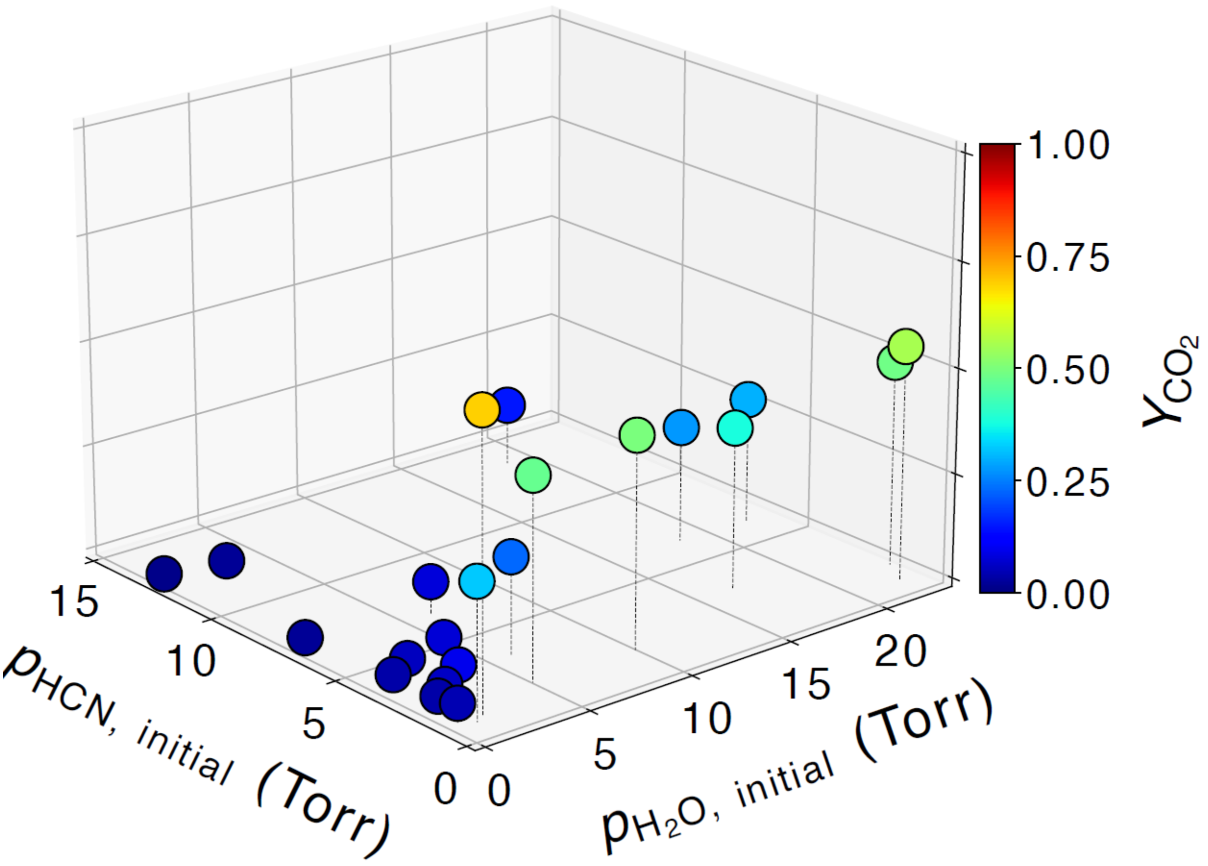

Figure S6: Y_{CO2} with respect to the initial partial pressures of HCN and H_2O.

This figure shows the relative amount of CO_2 in the gas phase after 420 s of irradiation and clearly, the amounts are much lower than in the case of CO, with the exception of experiments with very little HCN at the beginning and relatively high amounts of H_2O. Notably, in experiments with little to no H_2O, little to no CO_2 is formed. Even though CO is present in these experiments (as some residual water is always present in the experiment), it seems to be so little that all water is consumed for the formation of CO and none is left for the formation of CO_2.

Figure S7 shows the same property, the Y_{C2H2} for C_2H_2.

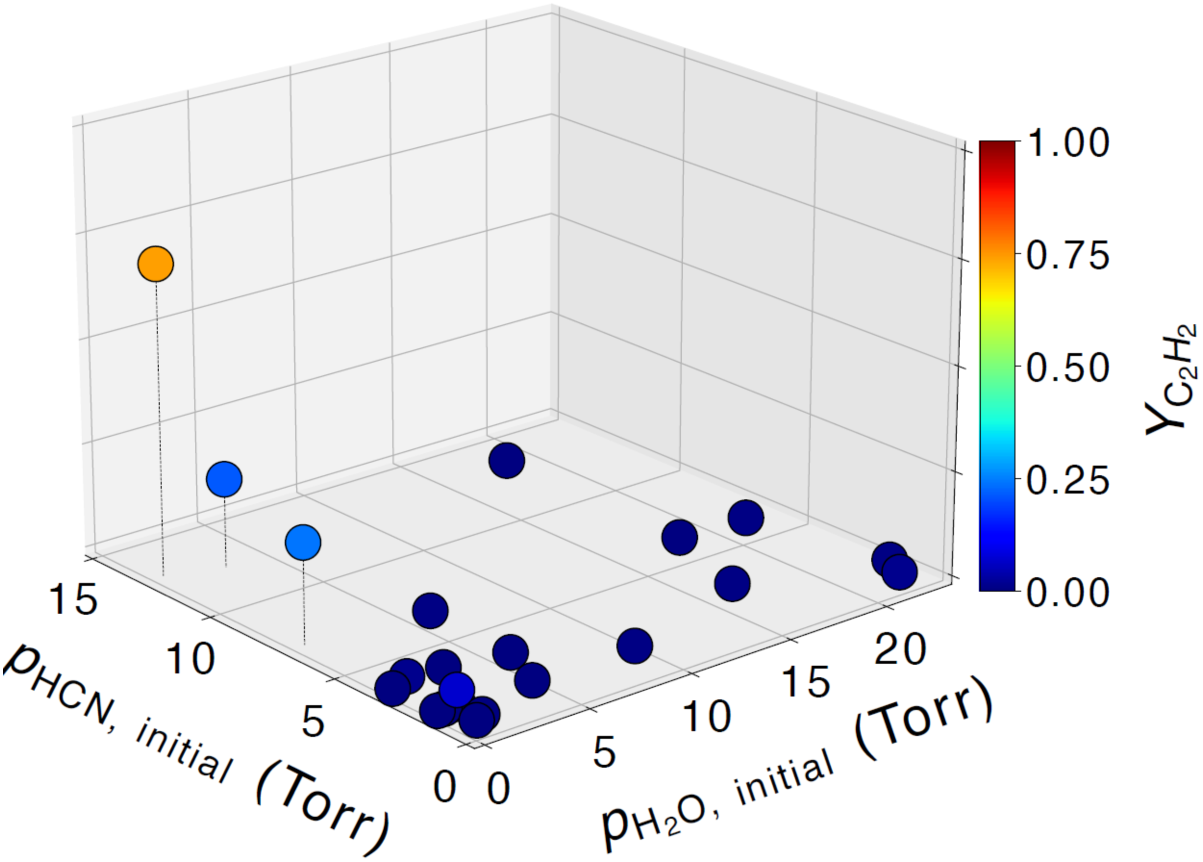

Figure S7: Y_{C2H2} with respect to the initial partial pressures of HCN and H_2O.

This figure shows the relative amount of carbon in the gas phase locked in acetylene. The formation of acetylene was observed only in three experiments. In these experiments, almost no water was initially present and the amounts of HCN were the highest of all the experiments. It can be expected the if even higher partial pressures of HCN were used, more acetylene could be formed. The actual observed amounts of acetylene were very small, but in the experiments where it was detected, little CO and CO_2 was formed as well. For this reason, in the one experiment with least water and most HCN at the beginning shows that up to 75% of the gas phase is acetylene. Most of the decomposition products of HCN in this experiment are solid products, which are not shown here but are shown in the main paper.

7 UV-Vis spectra

We observed the laser-induced dielectric breakdown in our experiments in hopes to reveal hints about the reaction mechanism. The spectra of the laser induced dielectric breakdown plasma in the main experiments were observed with a UV-ViS spectrometer (Aryelle Butterfly, LTB Lasertechnik Berlin, Germany) 10 ns after the pulse with a gate width of 1000 ns. The spectra were measured in two ranges (187--425 nm and 409--763 nm), as shown in panels A and B of Figure S8, respectively. Twenty measurements were averaged for each spectrum.

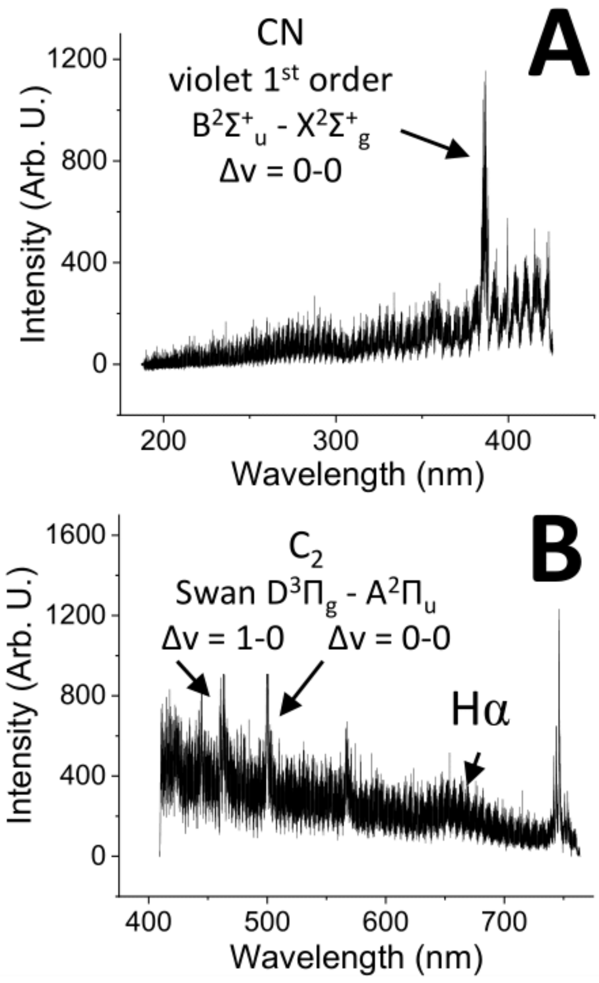

Figure S8: UV-ViS spectrum of the laser induced dielectric breakdown induced in a mixture of HCN (1.3 Torr) and H2O (0.3 Torr) by the Nd:YAG laser (as described in the experimental section of the main paper body.)

The spectra revealed signals of the .CN $B^{2\Sigma+}{}_u - X^{2\Sigma+}{}_g$ Δυ = 0-0 violet 1st, :C_2 Swan $D^{3\Pi}{}_g - A^{2\Pi}{}_u$ Δυ = 1-0 and Δυ = 0-0 transitions and the Hα line of atomic hydrogen. We also observed a signal at ~745 nm, which was not assigned. This measurement did not provide sufficient data to elucidate the reaction mechanism, but showed that the .CN radical, along with :C_2 and Hα are all present in significant amounts in the discharge. Interestingly, we did not observe any O:, .OH, N:. or N_2 emission bands at this or other gate delays.

8 Solid phase

The main focus of this study is the gas phase chemistry. Nevertheless, solid phase products form during the experiment as well. This manifests by forming a brownish layer on the walls of the irradiation cell.

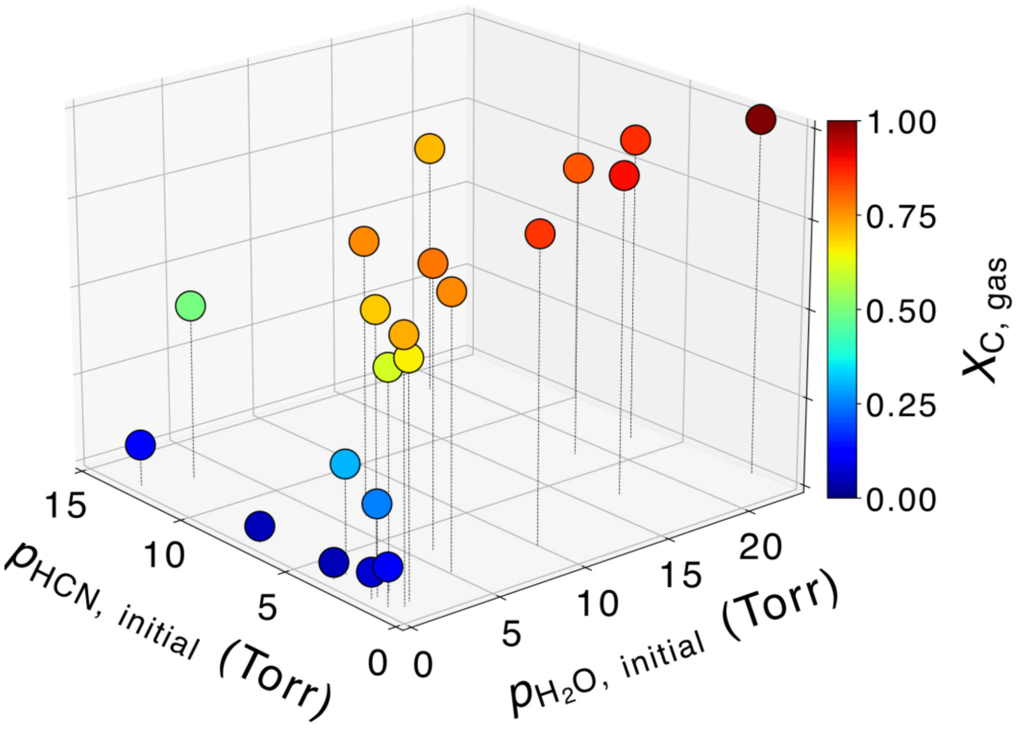

Figure S9: The relative proportion of gas phase products in the product mixture with respect to the initial amounts of H_2O and HCN.

Figure S9 visualizes the carbon distribution in the mixture in the form of its ratio in the gas phase vs. the solid phase.

This figure shows the relative proportion of carbon in the gas phase products in the product mixture, defined as:

$$X_{C,gas} = \frac{\sum_i p_i n_i}{p_{HCN}(t = 0s) - p_{HCN}(t = 420s)}$$

where p_i are the partial pressures of gas phase products CO, CO_2 and C_2H_2 and n_i is the amount of carbon atoms in the molecule.

The contribution of C_2H_2 is very small, but was included here nonetheless. This figure was created using the experimental data rather than the model output. It again shows that the contribution of direct HCN and H_2O decomposition is greater with the increasing amount of water at the start of the experiment. It also shows that higher initial amounts of HCN decrease the relative amounts of gas phase products. The reason for this in terms of mechanism can be the higher ratio of the densities of .CN and .OH in the plasma. This higher relative density increases the likelihood of collisions between two .CN radicals (likely producing solid polycyanes), while decreasing the probability of collisions between .CN and .OH (likely producing gas-phase radicals). Such behavior is expected from the model and confirms that the model and the experiment behave in the same manner.

The solid phase products were collected by washing the cell with methanol and then evaporating the solution.

The solid phase was analyzed by scanning electron microscopy. However, the majority of the evaporated sample constituted of Teflon™ bits scraped off the fan during use and of NaCl probably resulting from contamination of the sample during manipulation. Carbon signals expected from the experiment were also observed, but only in small amounts, which could be attributed both to the reaction and contamination. Any results from this solid phase analysis are therefore ambiguous. Based on the color and on existing literature, the main components of the solid phase should be tholins, soot and refractive carbon. We believe, however, that the solid phase would merit a thorough investigation. For this, the experiments would have to be optimized, such as carried out for longer times, with higher initial pressures.

Figure S10 below shows two photographs of the irradiation cell – before (A) and after irradiation (B), showing the solid phase.

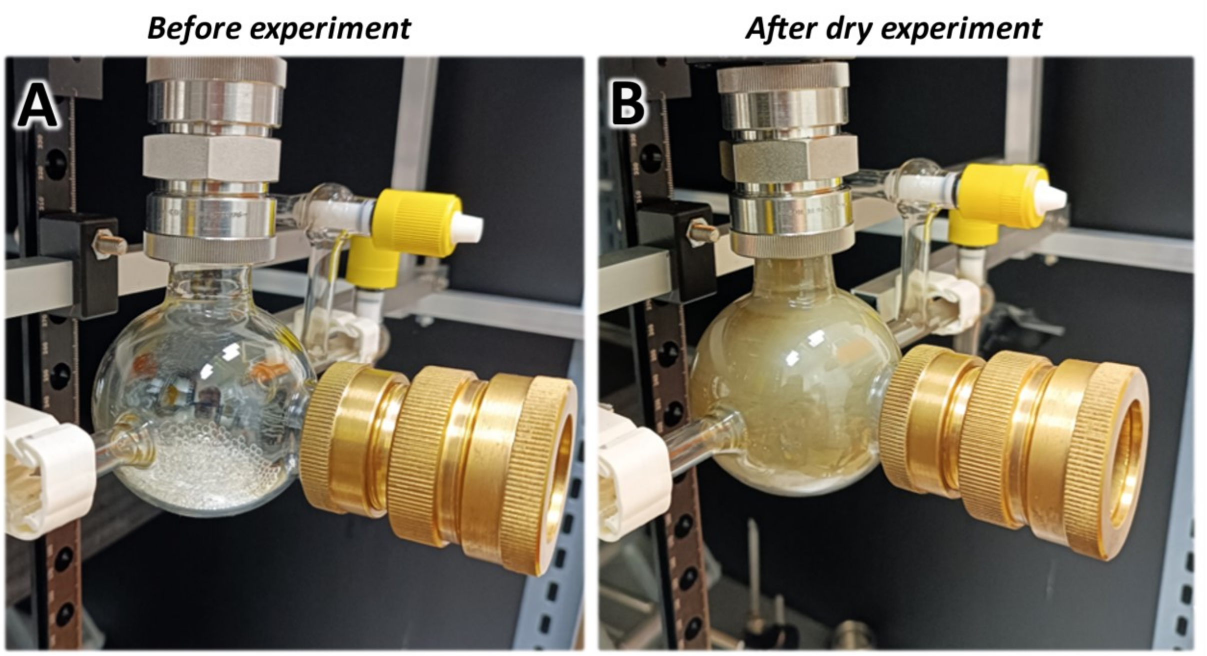

Figure S10: Photograph of the measurement cell before (A) and after irradiation (B). Solid phase formed during the experiment is clearly visible.

9 NO_xes

The mechanism proposed in this study is the dominant pathway that governs the formation of the CO-CO_2 product mixture, which is supported by the kinetics of the process described above. However, there exist side processes which lead to different products. The most efficient of those side processes are the formation of NO and N_2O. Both NO and N_2O were observed in two experiments (experiments 8 and 9 as described in the main text). Figure S11 shows FTIR spectrum of the gas phase of experiment 8 after 2520 s of irradiation. This experiment initially contained ~1.25 Torr of HCN, ~13.25 Torr of H_2O and ~706 Torr of N_2. These two experiments, 8 and 9, are the experiments performed with the highest initial amount of water. It is likely, therefore, that the formation of the NO_xes includes H_2O in this mechanism. These molecules were, however, observed only in these two experiments and their partial pressure did not exceed 0.1 Torr. For this reason, we did not consider those products in the main mechanism.

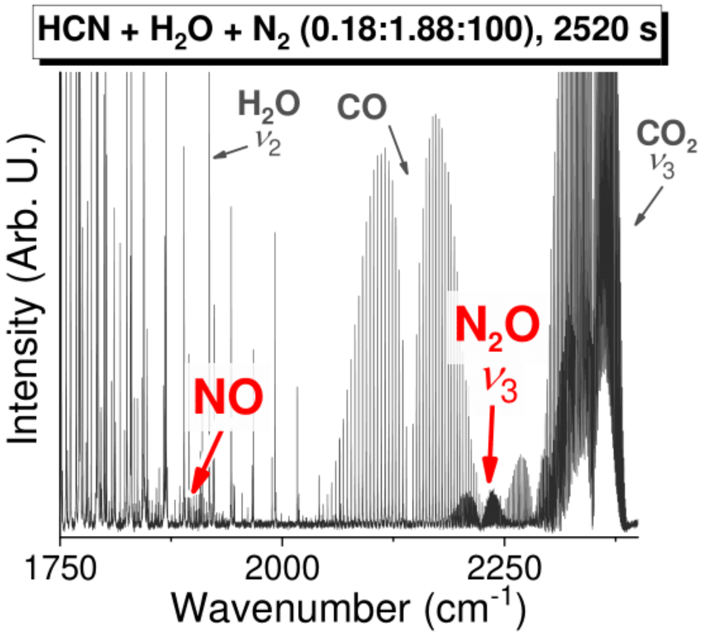

Figure S11: A FTIR spectrum of experiment 9 in the main table after 2520 s of laser irradiation. Shown in red are the observed bands of NO and the v_3 band of N_2O.

10 HCN laboratory synthesis

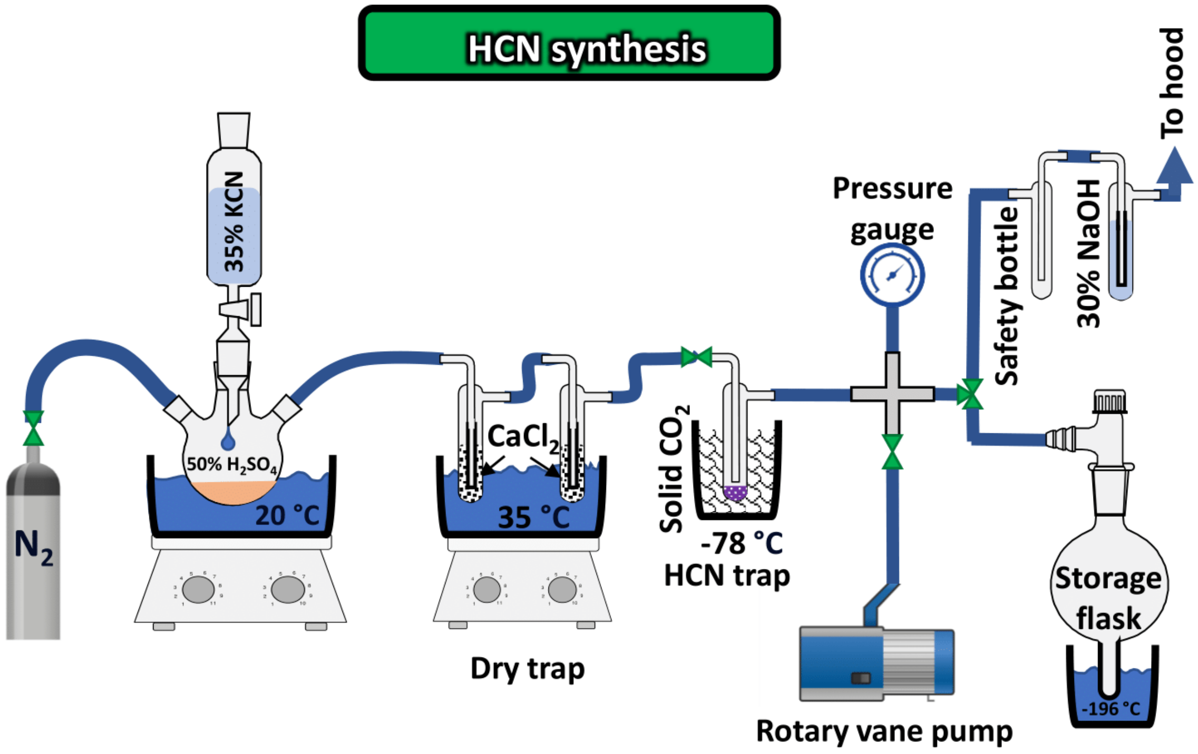

Figure S12: The apparatus and synthetic process of HCN used for this paper.

HCN was necessary as a reactant for the experiments shown in this paper. We developed a new protocol for its synthesis (shown in Figure S12):

- Preheat $CaCl_2$ traps to avoid condensation of HCN.
- Purge the apparatus with N_2 to remove air.
- Precool HCN condensation trap with CO_2(s).
- Keep a small flow of N_2.
- Add 35 wt.% KCN dropwise to a constantly stirred 50 wt.% H_2SO_4. Add ~1 drop per second - the goal is to add KCN slow enough to keep the solution cool enough so that the HCN does not polymerize (indicated by a yellow brown color). Carefully collect all HCN vapors produced.
- After all KCN is added, heat the solution to 70-80 ℃ to evaporate the leftover HCN from the solution.
- Let cool to room temperature.
- Stop the flow of N_2 and evacuate the central part of the apparatus.
- Close off the central part of the apparatus, let the HCN evaporate and then freeze out in liquid nitrogen in the storage flask.
- Flush the whole apparatus thoroughly with N_2.
- Add 30 wt.% NaOH to the H_2SO_4 solution.
- Add NaOCl to the solution to neutralize any remaining NaCN.

11 Decomposition fluxes for planets

Table S2 below shows decomposition fluxes calculated based on our model for the minimum and maximum HCN decomposition rates at given time and composition. The maximal values correspond to the initial amount of 13 Torr HCN and 20 Torr H2O, while the minimal ones correspond to 0.5 Torr HCN without any H2O. The given time intervals (number of pulses/energy delivered to the system) were chosen as 0.1 s (1 pulse), 106 s as same delivered energy as (McKay and Borucki, 1997, 10.1126/science.276.5311.390), 420 s (4200 pulses) as approximate HCN half-life, 2500 s (25 000 pulses) as the time when derivations of maximal and minimal curves begin to be similar and 7000 s (70 000 pulses) s the end of experiments. This purpose of this table is to illustratively show outcomes of the model in its boundary conditions.

Table S2: Minimum and maximum HCN decomposition values based on our model calculated from our model at given times for Venus, Earth and Mars.

					Venus		Earth		Mars	
t (s)	Number of pulses	E (J)	HCN decomp. rate (molec J^{-1})		HCN decomp. Flux (cm-2 s-1)					
			max	min	max	min	max	min	max	min
0.1	1	0.45	5.37E+17	2.43E+15	4.52E+12	2.05E+10	1.63E+13	7.41E+10	3.40E+12	1.54E+10
106	1060	477	3.25E+17	2.33E+15	2.75E+12	1.97E+10	9.92E+12	7.11E+10	2.07E+12	1.48E+10
420	4200	1890	1.45E+17	2.07E+15	1.22E+12	1.75E+10	4.42E+12	6.30E+10	9.20E+11	1.31E+10
900	9000	4050	7.71E+16	1.73E+15	6.52E+11	1.46E+10	2.35E+12	5.29E+10	4.90E+11	1.10E+10

2500	25000	11250	2.91E+16	1.05E+15	2.46E+11	8.89E+09	8.89E+11	3.21E+10	1.85E+11	6.68E+09
4000	40000	18000	1.84E+16	7.29E+14	1.56E+11	6.16E+09	5.61E+11	2.22E+10	1.17E+11	4.63E+09
7000	70000	31500	1.07E+16	4.33E+14	9.01E+10	3.66E+09	3.25E+11	1.32E+10	6.77E+10	2.75E+09

Similar to the main text, based on the results of our experiments at the given HCN and H2O ranges, we present in Table S3 a maximum flux of HCN decomposition during LHB for Earth, Venus, and Mars with values of 1.63e+13, 4.52e+12 and 3.40e+12 cm^{-2} s^{-1} respectively. These were obtained from a model atmosphere containing 13.0 Torr HCN (~1.7 vol. %) and 20.0 Torr H2O (2.8 vol. %) and correspond to one laser pulse's energy (0.1 s, at 10 Hz laser repetition) delivered to the model gas mixture. At these conditions, the above values are the highest ones to be reached.

The minimum HCN decomposition fluxes were determined from experiment containing 0.5 Torr HCN (~0.07 vol. %) and almost no water (~0.01 Torr) with values of 7.41e+10, 2.05e+10 and 1.54e+10 cm^{-2} s^{-1} for Earth, Venus and Mars respectively. This experiment, chosen as 0.5 Torr HCN and no H2O, was the one with the lowest initial amount of HCN. Even if our model can extrapolate any inlet values to 0, we cannot guarantee reaching viable results below the experimental limit of 0.5 Torr HCN. Any other values that lay in our HCN vs H2O range can be calculated using our system rate coefficients. This extended table includes calculations for points x1 – x9.

Table S3: Maximum and minimum HCN decomposition fluxes (cm-2 s-1) for Venus, Earth and Mars calculated from our model. The calculations were performed at 0.1 s of irradiation (1 pulse) for different compositions (indicated).

	Venus	Earth	Mars
R [km]	6058	6378	3389.5
<Ei vi> [J * yr-1]	9.17E+12	1.56E+14	3.98E+13

	HCN (initial) [Torr]	H2O (initial) [Torr]	C/O	Maximum HCN decomposition flux [cm-2 s -1]		
x1	1	1	1.00	5.65e+10	2.04e+11	4.25e+10
x2	1	9	0.11	1.80e+11	6.48e+11	1.35e+11
x3	1	18	0.06	3.18e+11	1.15e+12	2.39e+11
x4	6.5	1	6.50	3.67e+11	1.32e+12	2.76e+11
x5	6.5	9	0.72	1.17e+12	4.21e+12	8.77e+11
x6	6.5	18	0.36	2.06e+12	7.45e+12	1.55e+12
x7	12	1	12.00	6.78e+11	2.45e+12	5.09e+11
x8	12	9	1.33	2.15e+12	7.77e+12	1.62e+12
x9	12	18	0.67	3.81e+12	1.37e+13	2.86e+12
max	13	20	0.65	4.52e+12	1.63e+13	3.40e+12

min	0.5	0	-	2.05e+10	7.41e+10	1.54e+10

Similarly, Table S4 below shows HCN decompositions rates calculated from our model for maximum and minimum partial pressures compositions (12.5 Torr HCN + 20 Torr H2O and 0.5 Torr HCN and 0 Torr H2O) at different times.

Table S4: HCN decomposition rates calculated for maximum and minimum partial pressure compositions and various irradiation times.

	HCN (initial)	H2O (initial)	C/O	HCN decomposition rate [molec J^{-1}]			
	Torr	Torr		0.1 s	106 s (McKay)	420 s	4000 s
x1	1	1	1.00	6.69E+15	6.22e+15	5.11e+15	1.43e+15
x2	1	9	0.11	2.13E+16	1.71e+16	1.04e+16	1.39e+15
x3	1	18	0.06	3.77E+16	2.67e+16	1.29e+16	1.44e+15
x4	6.5	1	6.50	4.35E+16	3.96e+16	3.17e+16	9.35e+15
x5	6.5	9	0.72	1.38E+17	1.05e+17	5.96e+16	9.07e+15
x6	6.5	18	0.36	2.45E+17	1.63e+17	7.74e+16	9.22e+15
x7	12	1	12.00	8.02E+16	7.18e+16	5.65e+16	1.73e+16
x8	12	9	1.33	2.55E+17	1.83e+17	9.89e+16	1.69e+16
x9	12	18	0.67	4.52E+17	2.83e+17	1.31e+17	1.69e+16
max	12.5	20	0.63	5.17E+17	3.24e+17	1.45e+17	1.84e+16
min	0.5	0	50.00	2.43E+15	2.33e+15	2.07e+15	7.29e+14

12 Applicability range

As outlined above and detailed in Table 1, we conducted 21 additional single-point experiments with a broader range of initial HCN and H_2O concentrations. This expansion of the experimental parameter space enhances the applicability of the global rate coefficients for investigating plasma chemistry in planetary atmospheres. The initial composition at the start of each experiment was used to predict the gas phase composition after 420 seconds of irradiation. The computed predictions were subsequently compared with the corresponding experimental results. Given that each molecule's partial pressure was represented by a single data point from the 420-second irradiation interval, we introduced a modified version of the R^2 descriptor to evaluate the fit's accuracy.

We define this modified metric as R^2-like', which is described as follows:

$$R^2\text{like} = 1 - \frac{(p_{i,\text{obs}} - p_{i,\text{calc}})^2}{{p_{i,\text{obs}}}^2} \quad (12)$$

where $p_{i,obs}$ (Torr) is the experimentally observed partial pressure of compound *i* and $p_{i,calc}$ (Torr) is the calculated counterpart. The R^2-like indicator was calculated for each species (HCN, H_2O, CO and CO_2)

and then averaged. The result is shown in Figure S13 and the individual R^2-like value are shown in the Supplementary Information.

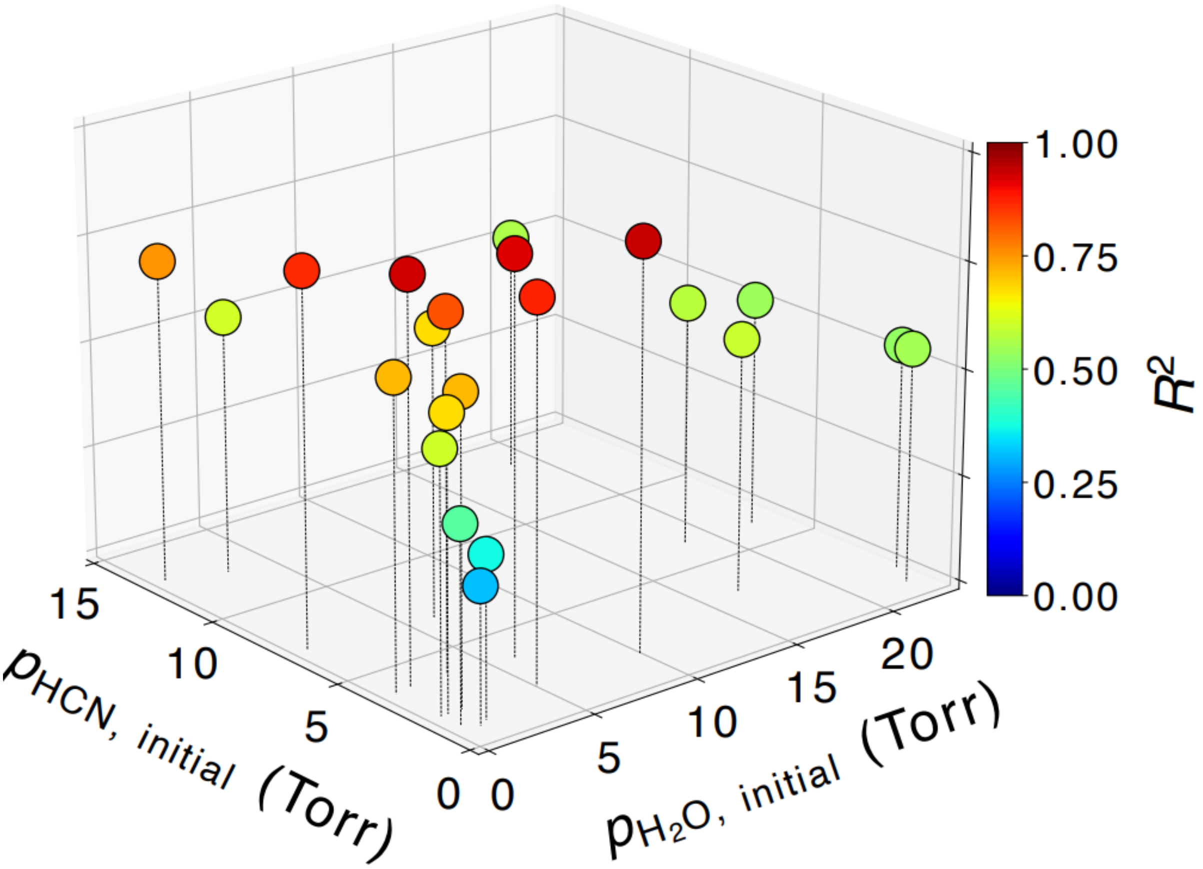

Figure S13: R^2-like indicator of the goodness of fit for each of the additional 420 s (1890 J) experiments plotted versus the initial contents of HCN and H_2O.

Our model correctly predicts HCN and H_2O partial pressures at 420 s in the whole range of initial conditions (i.e., HCN initial partial pressures <13 Torr and H_2O initial pressures <20 Torr). The accuracy of the fit slightly, but reasonably, decreases when either of these components' initial pressure exceeds ~10 Torr. This decrease is likely attributable to adsorption or co-adsorption, as previously described. For CO, the prediction is sufficient for all tested partial pressures with a slightly worse result for initial partial pressures of HCN and H_2O <1 Torr. The partial pressure of CO_2 is well predicted for initial partial pressures of HCN and H_2O ~1-5 Torr. For other pressures, the R^2-like indicator is negative, which would imply a wrong prediction. However, upon reviewing the data, it is evident that the absolute differences between the predicted and observed partial pressures of CO_2 do not exceed ~0.1 Torr. Due to the nominal values of the partial pressures of CO_2 being very small and the measurement error remaining relatively constant, the relative errors are high. Therefore, for CO_2, this R^2-like indicator is not a good descriptor of the fit.

In summary, our experiments covered a parameter space of initial partial pressures ranging from approximately 0 to 13 Torr (0-1.8%) for HCN and from 0 to 20 Torr (0--2.7%) for H2O. The data are therefore suitable for atmospheric plasma chemistry models with partial pressures of HCN and H_2O within this range. Furthermore, since the range covers water vapor pressures up to the saturated vapor

pressure and HCN content up to 1.8%, this range should be sufficient to cover the vast majority of known planetary systems.

The model enables calculation of the composition of the experimental mixture at any point in time and any initial composition within the established experimental parameter space ~0-13 Torr (0-1.8%) of HCN and ~0--20 Torr (0-2.7%) of H_2O. Calculated results of the impact HCN decomposition are shown in Figure S14.

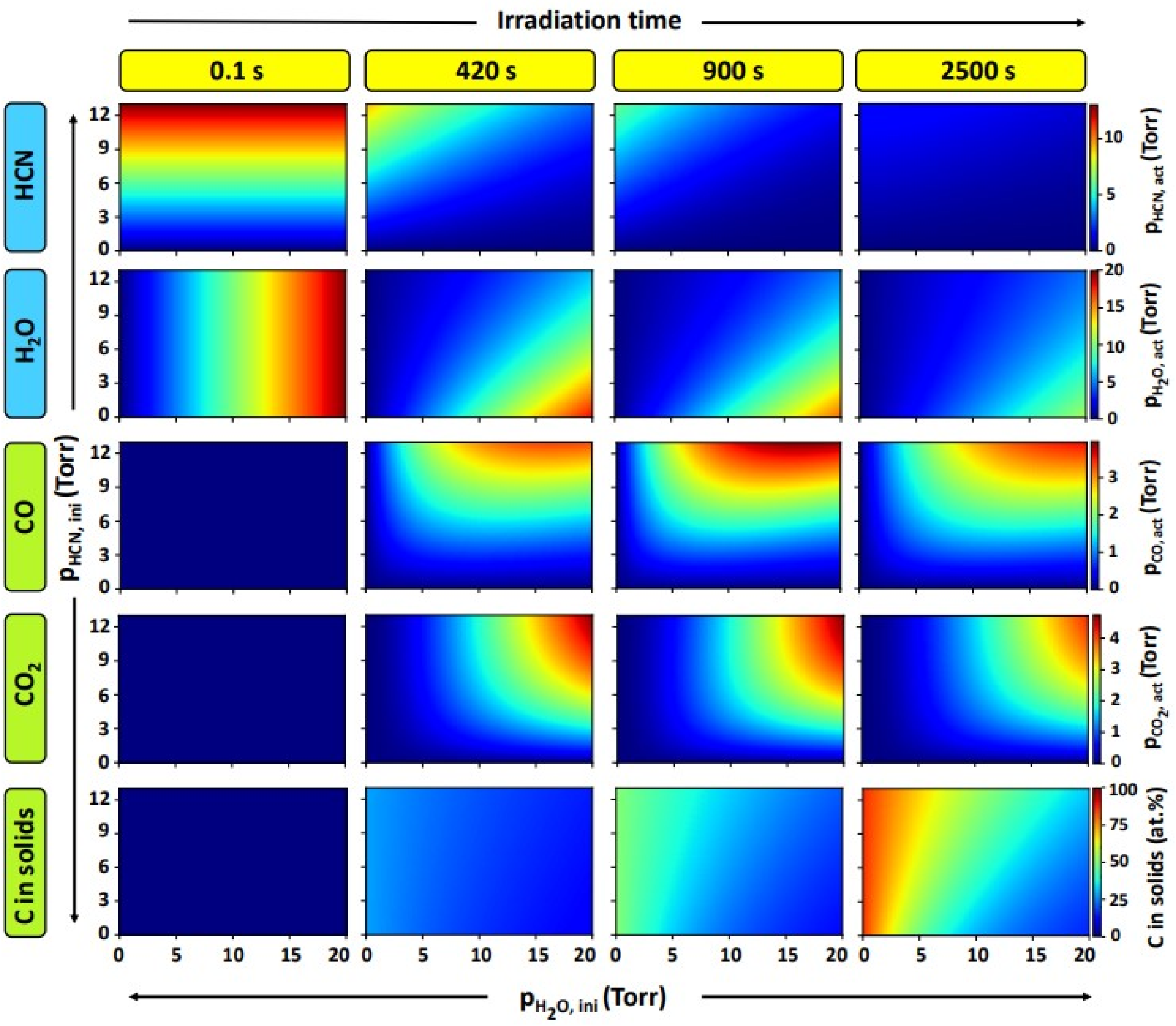

Figure S14: Model results of the HCN decomposition. Each column represents a fixed irradiation time indicated at the top. Each row then shows results for a given reactant or products. Colorbars for the presented results are depicted on the right.

There, partial pressures and the amount of carbon deposited to solid phase are depicted for all initial partial pressures at four selected times – 0.1 s, 420 s, 900 s, and 2500 s. The last row (C in solid phase) illustrates that the drier the atmospheric mixture, the more carbon ends up in the solid phase. The fraction of this solid phase was calculated as follows:

$$C_{\text{in solids}}\ (\text{at.}\,\%) = \left(1 - \frac{p_{\text{HCN,immediate}} + p_{\text{CO,immediate}} + p_{\text{CO}_2\text{,immediate}}}{p_{\text{HCN,initial}}}\right) \times 100$$

Interestingly, at 2500 s, even CO_2 partial pressures are lower than at 900 s. Even though CO_2 is the final product of one of the reaction channels in the model, all reactions in the channel are reversible. On the other hand, decomposition of HCN into solid products is irreversible, not least because the solid products are no longer in contact with the gas phase LIDB. Therefore, at very high irradiation times, all carbon in the model will be transformed to solid refractory material.

13 Model snapshots

Similar to Figure 7 in the main text, the section below shows snapshots of the model for various species and combinations of species. These snapshots provide information on the behavior of the model and the composition of the mixture in time across various composition.

HCN decomposition rate

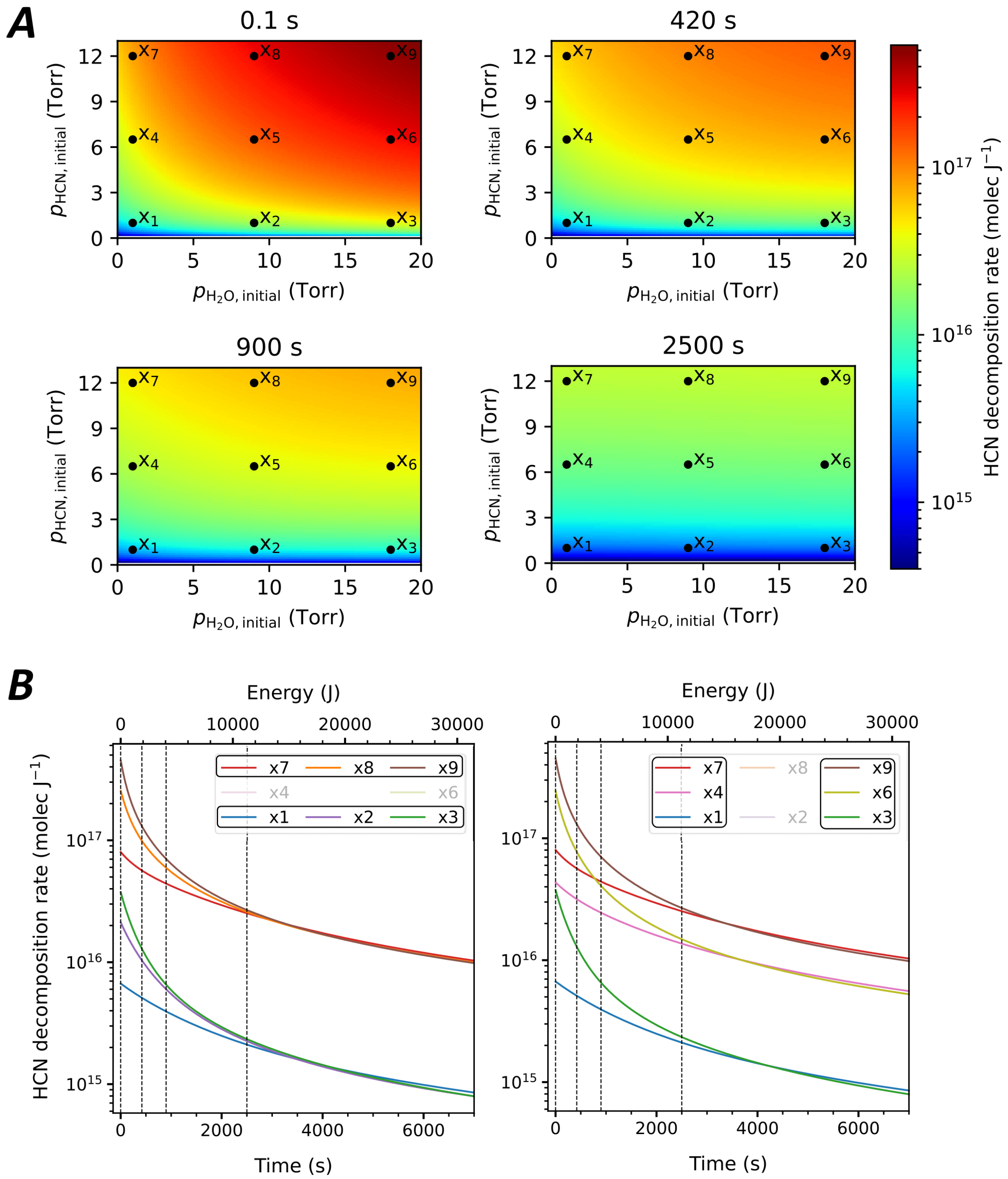

Figure S15: HCN decomposition rate calculated from our model. Panel A shows results for four selected times, panel B shows results for selected compositions.

HCN evolution

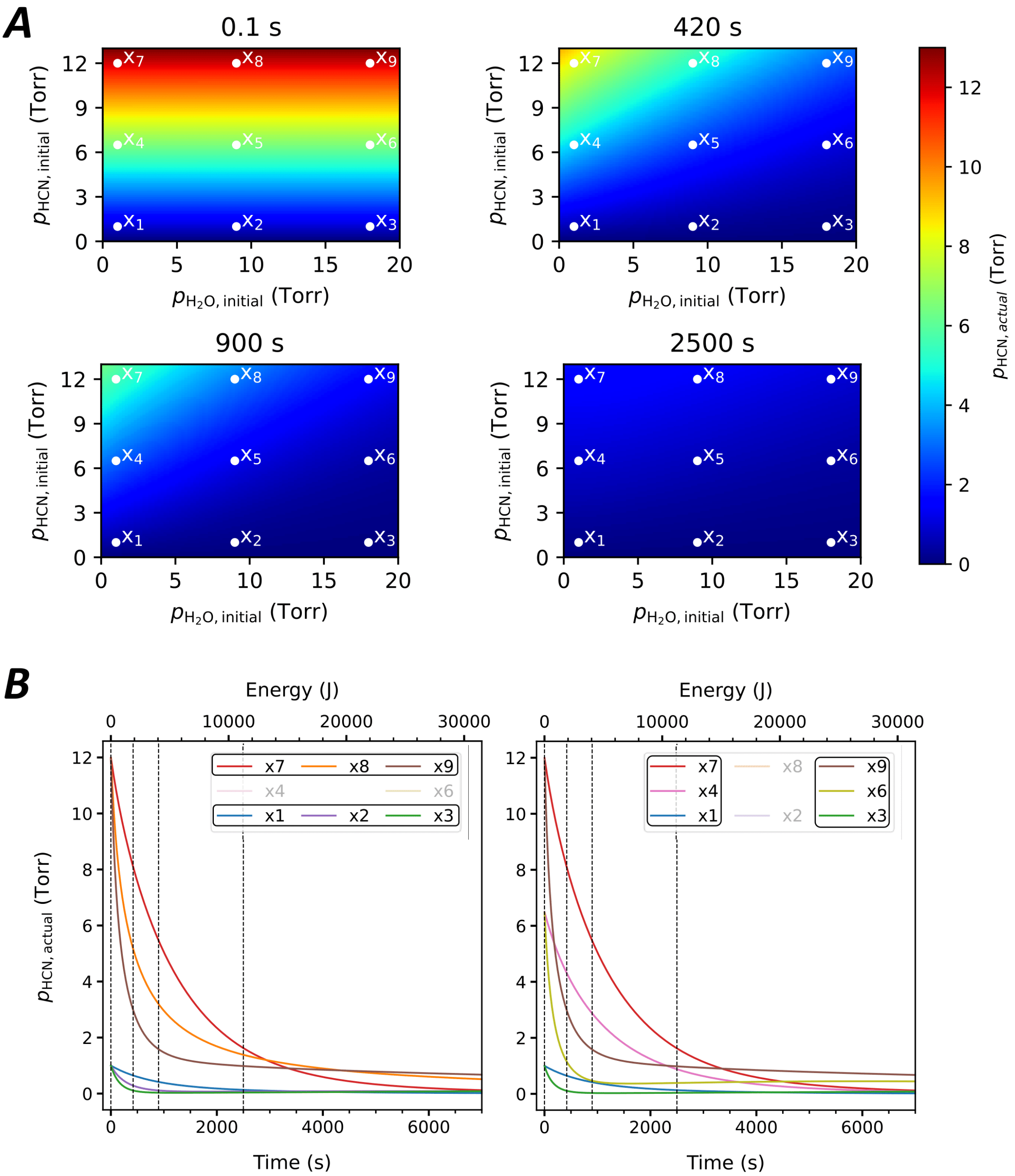

Figure S16: HCN partial pressure calculated from our model. Panel A shows results for four selected times, panel B shows results for selected compositions.

H_2O evolution

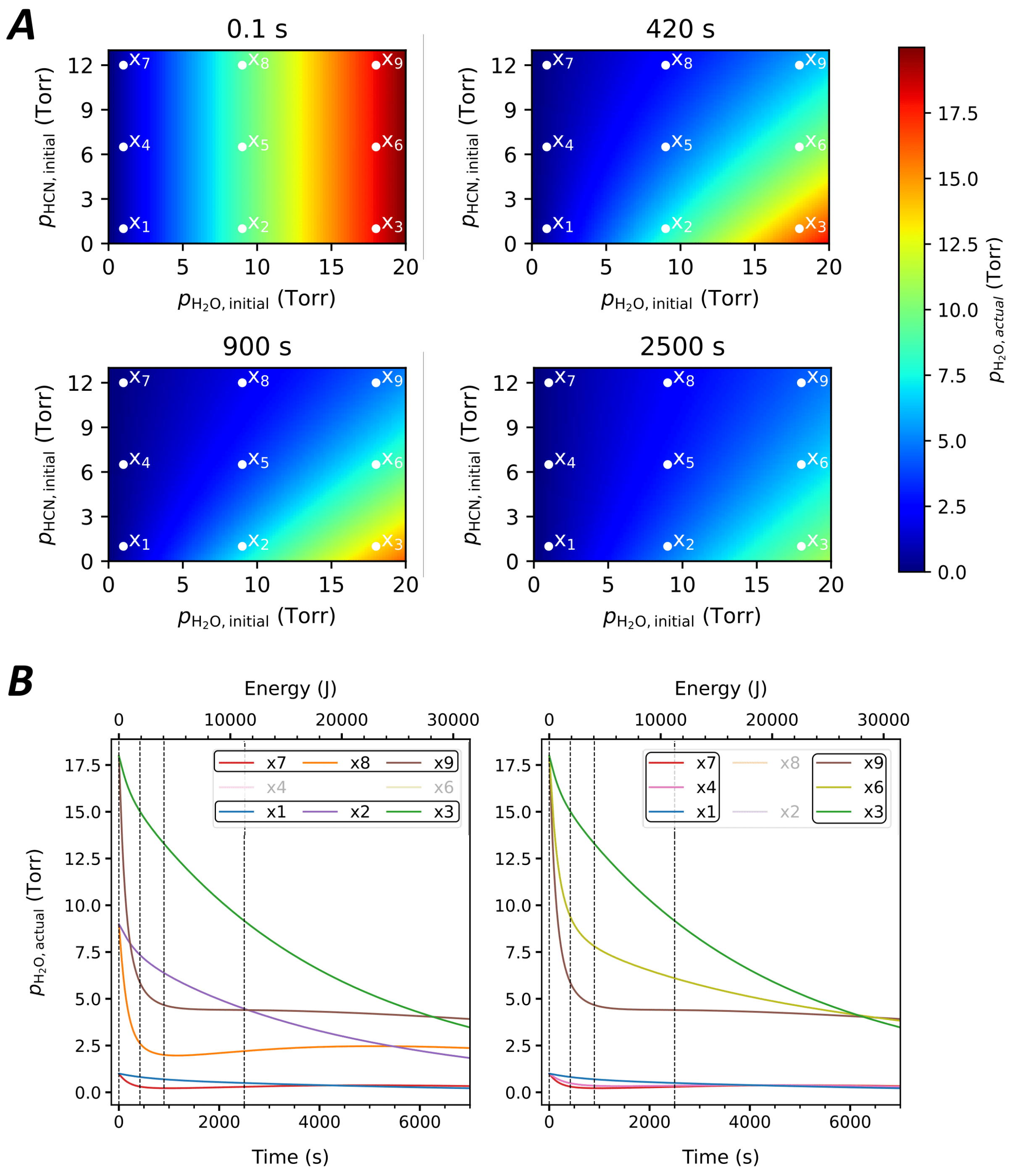

Figure S17: H_2O partial pressure calculated from our model. Panel A shows results for four selected times, panel B shows results for selected compositions.

CO evolution

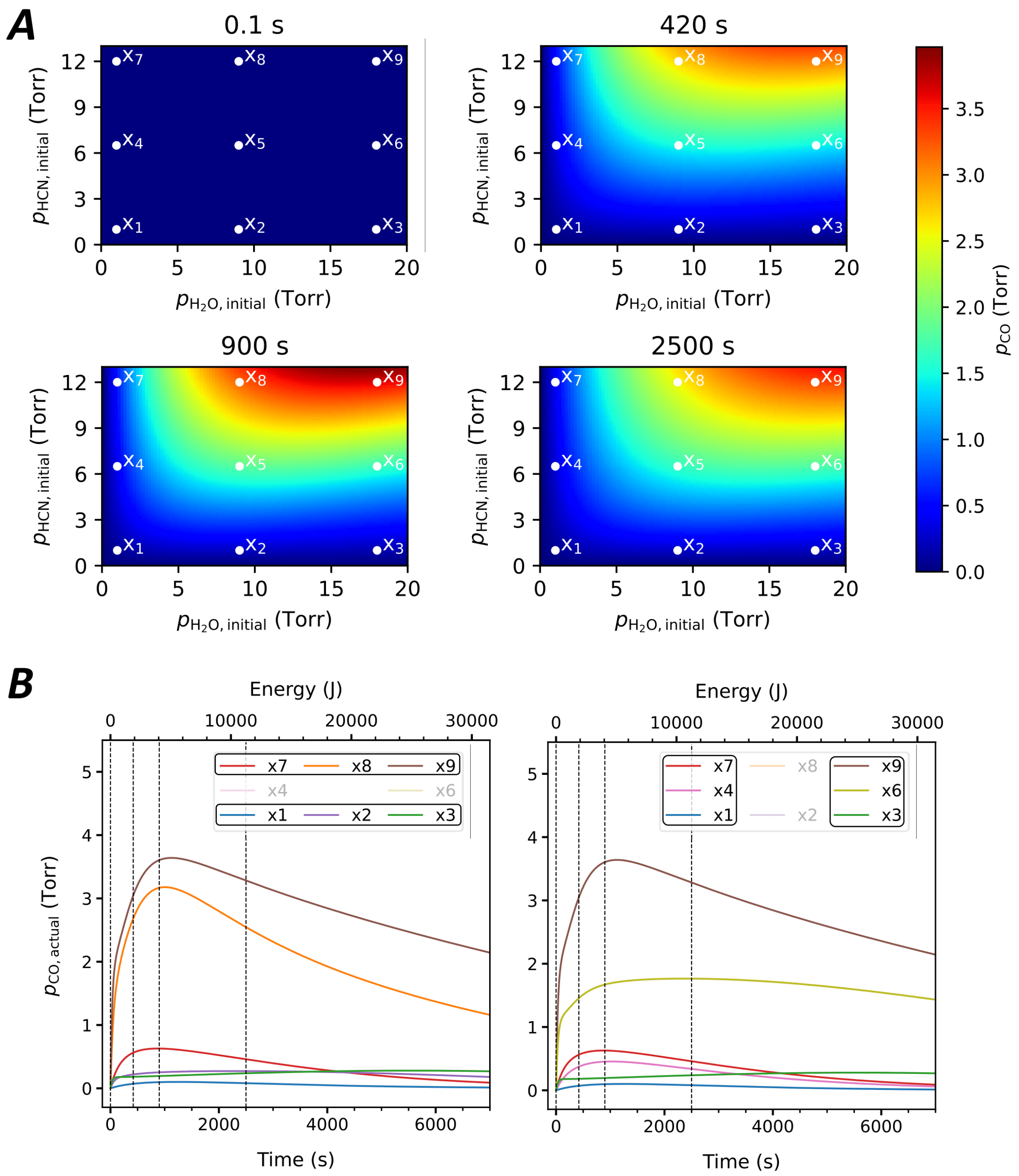

Figure S18: CO partial pressure calculated from our model. Panel A shows results for four selected times, panel B shows results for selected compositions.

CO_2 evolution

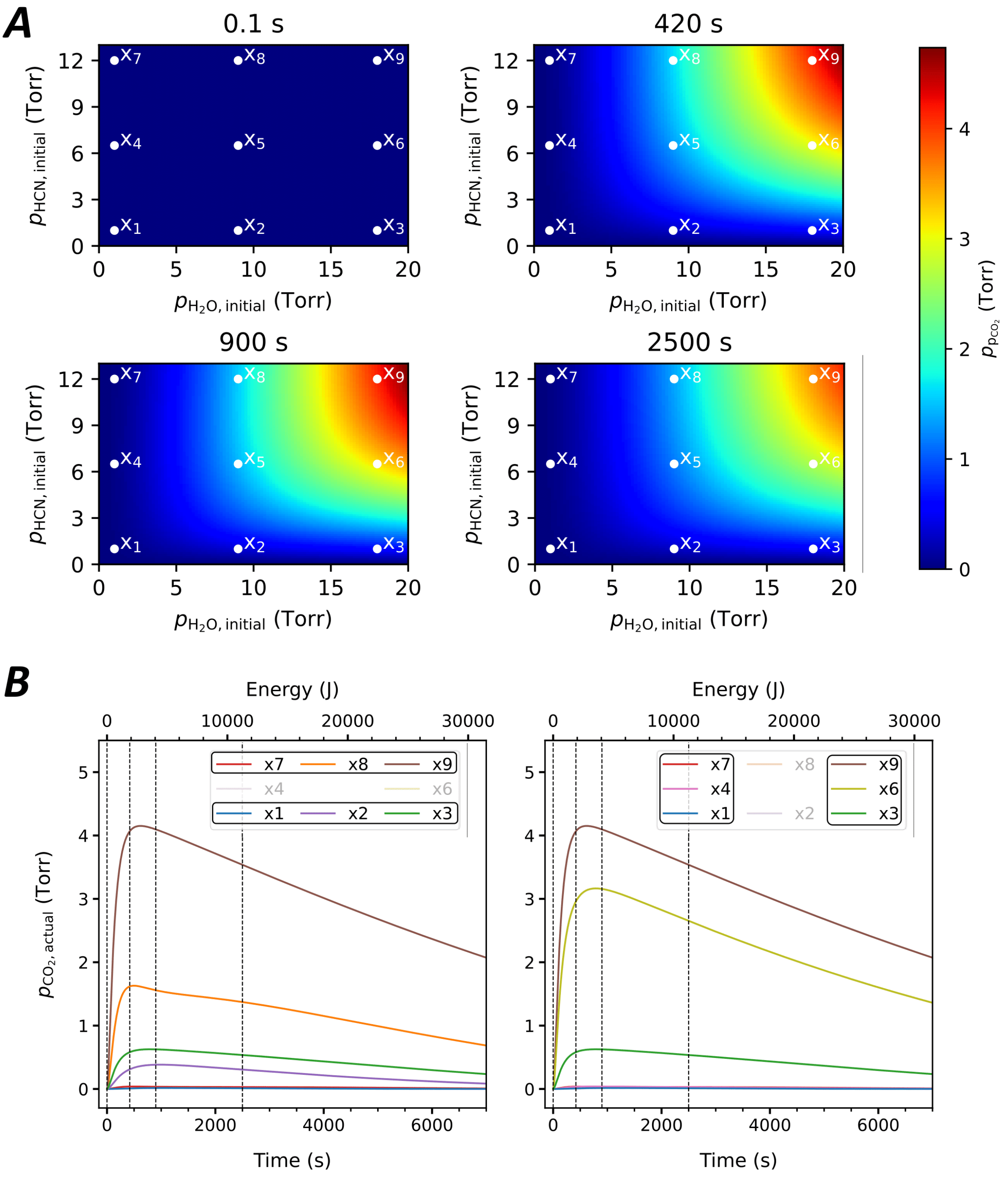

Figure S19: CO_2 partial pressure calculated from our model. Panel A shows results for four selected times, panel B shows results for selected compositions.

CO + CO_2 evolution

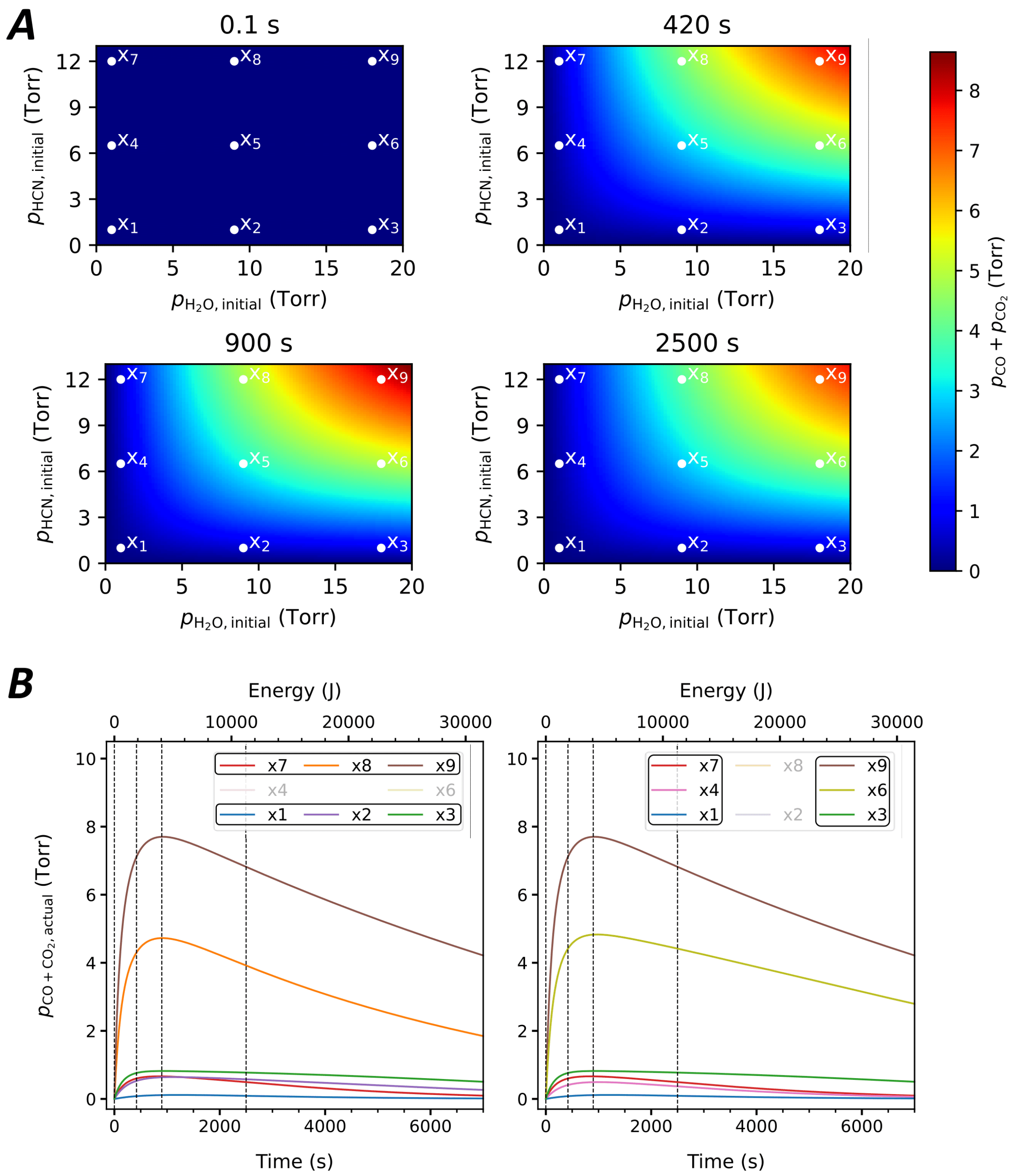

Figure S20: CO + CO_2 partial pressure calculated from our model. Panel A shows results for four selected times, panel B shows results for selected compositions.

C in solid phase evolution (1)

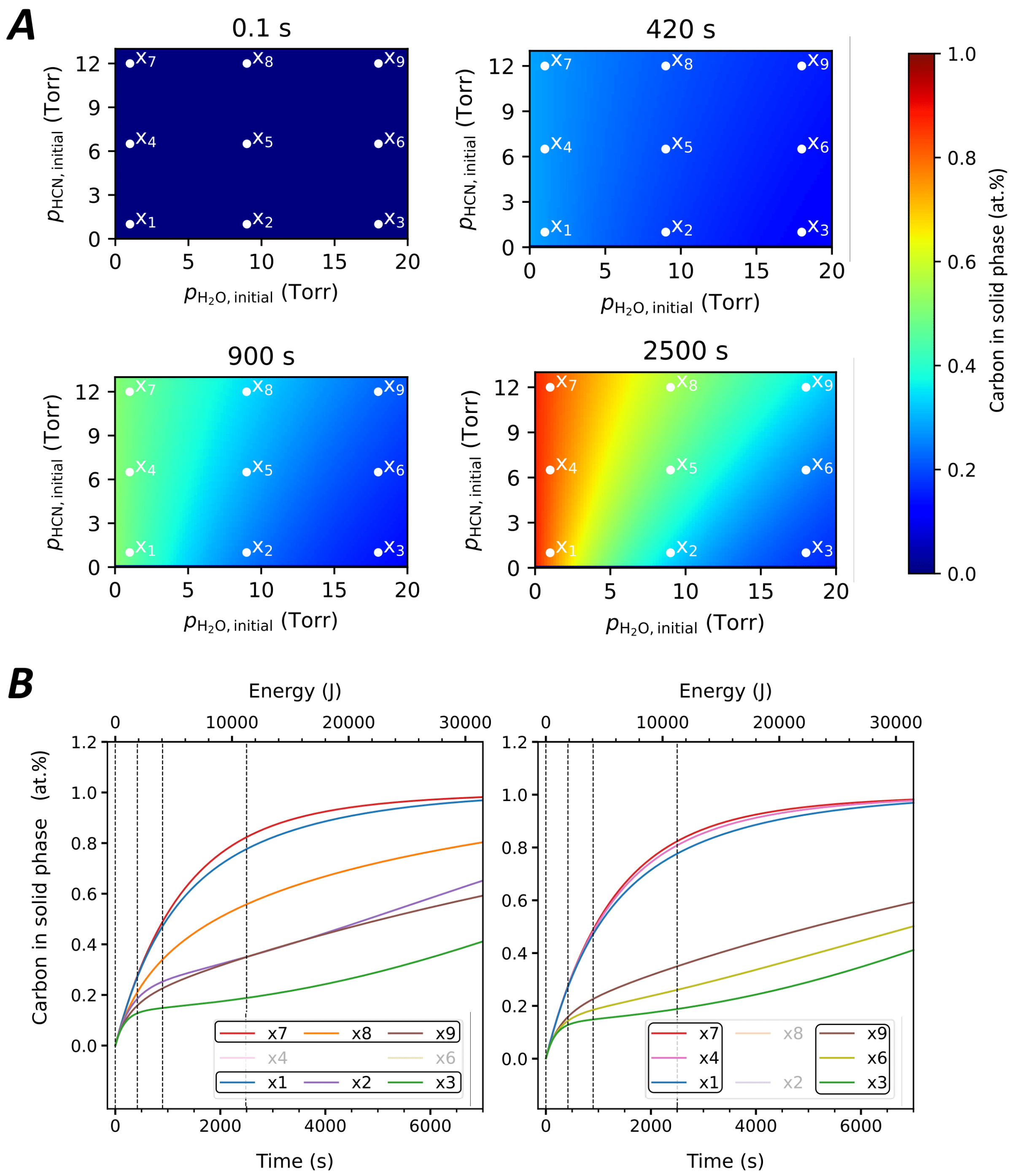

Figure S21: Amount of solid phase carbon partial pressure calculated from our model. Panel A shows results for four selected times, panel B shows results for selected compositions.

C in solid phase evolution (2)

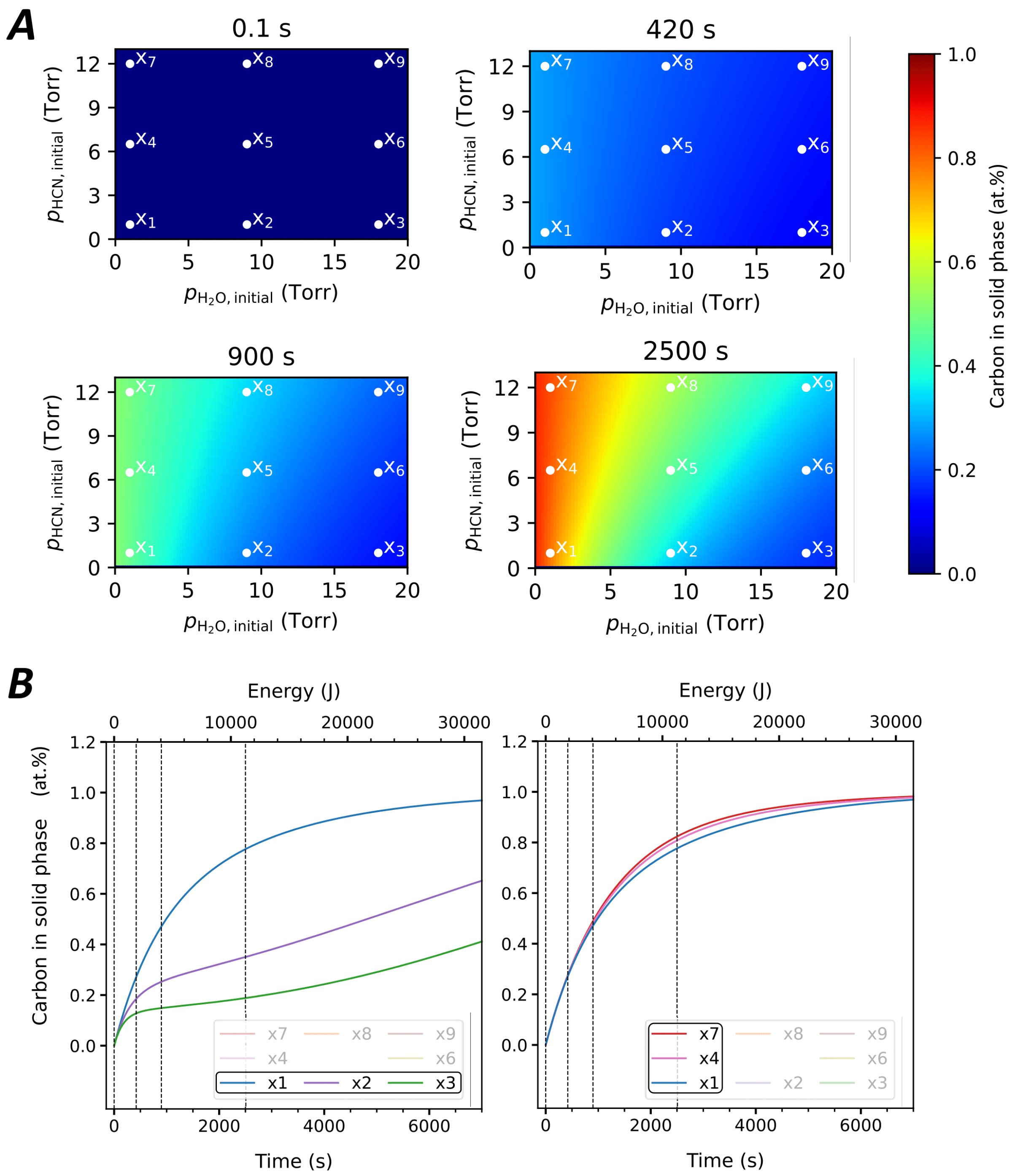

Figure S22: Amount of solid phase carbon partial pressure calculated from our model. Panel A shows results for four selected times, panel B shows results for different selected compositions.

C in solid phase evolution (3)

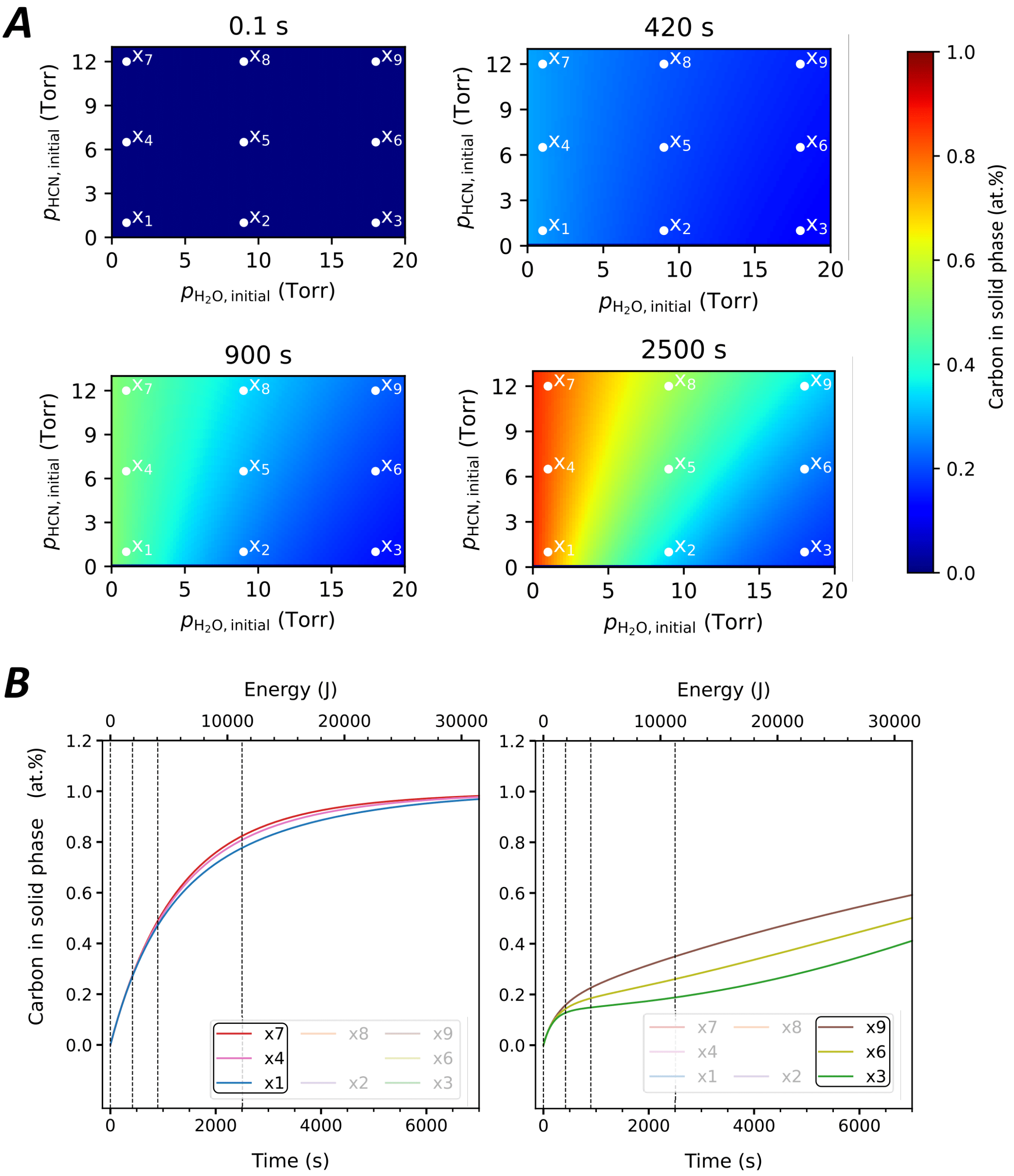

Figure S23: Amount of solid phase carbon partial pressure calculated from our model. Panel A shows results for four selected times, panel B shows results for different selected compositions.

C in solid phase evolution (4)

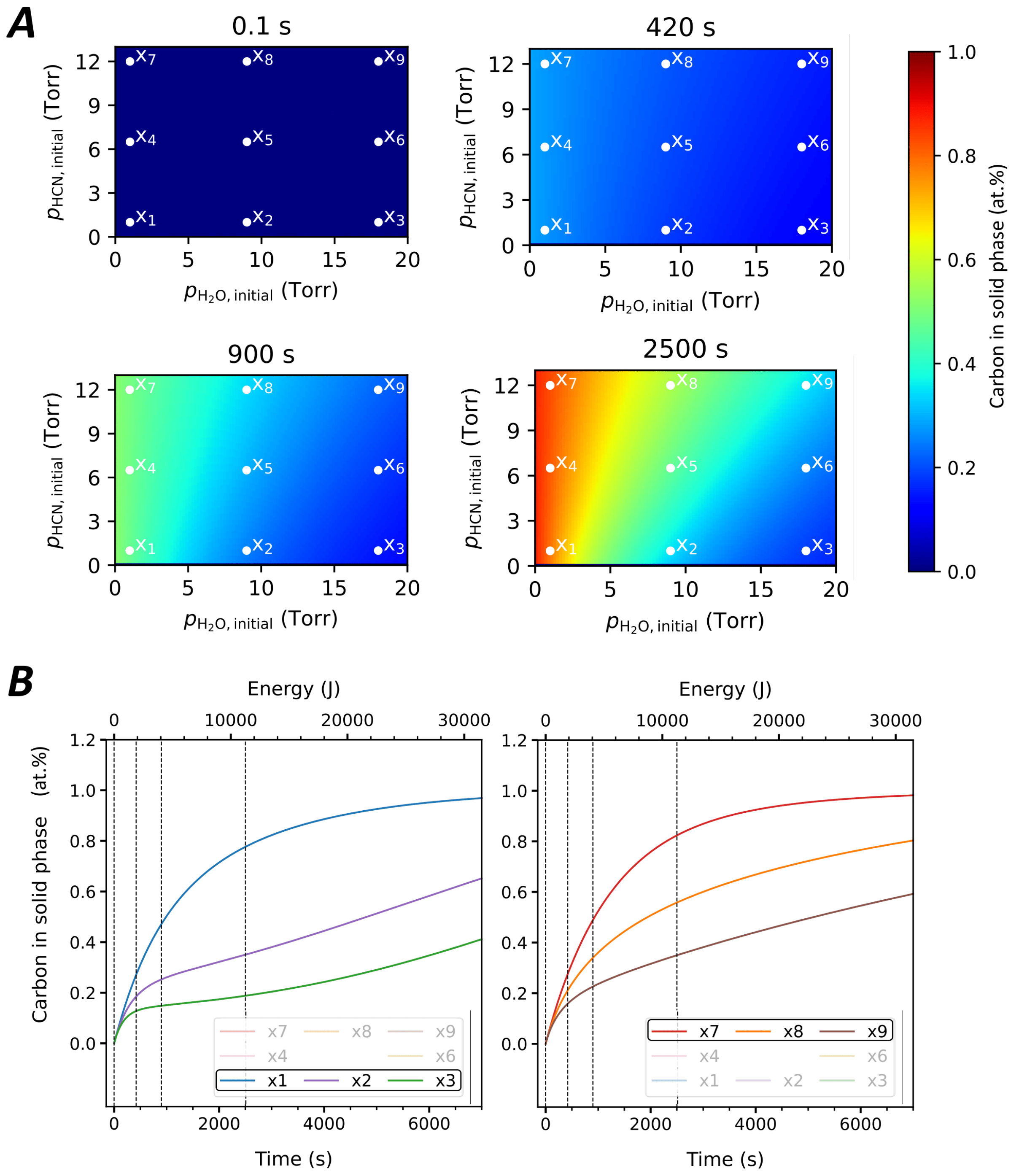

Figure S24: Amount of solid phase carbon partial pressure calculated from our model. Panel A shows results for four selected times, panel B shows results for different selected compositions.

C in solid phase evolution (5)

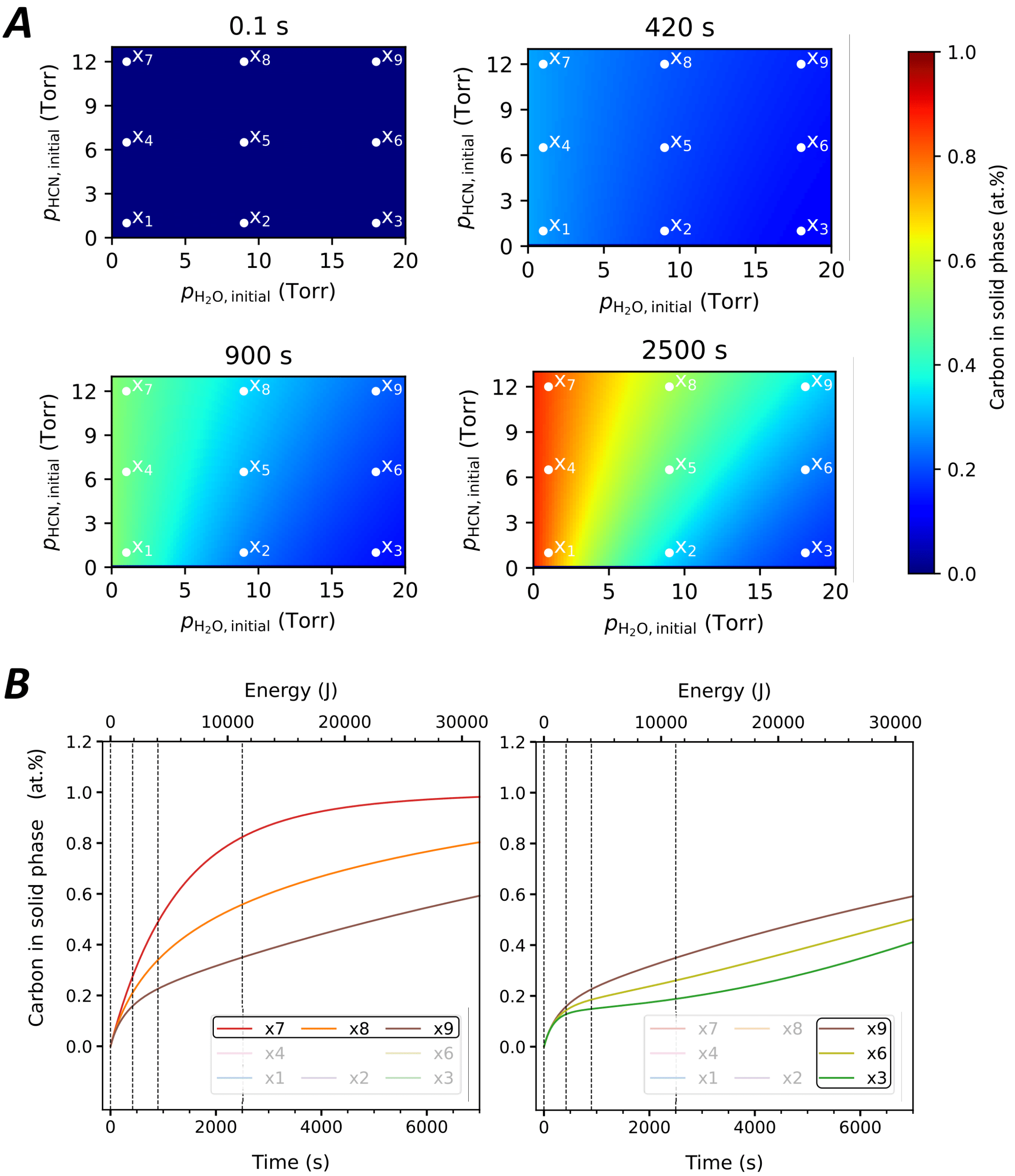

Figure S25: Amount of solid phase carbon partial pressure calculated from our model. Panel A shows results for four selected times, panel B shows results for different selected compositions.

C in gas phase evolution (1)

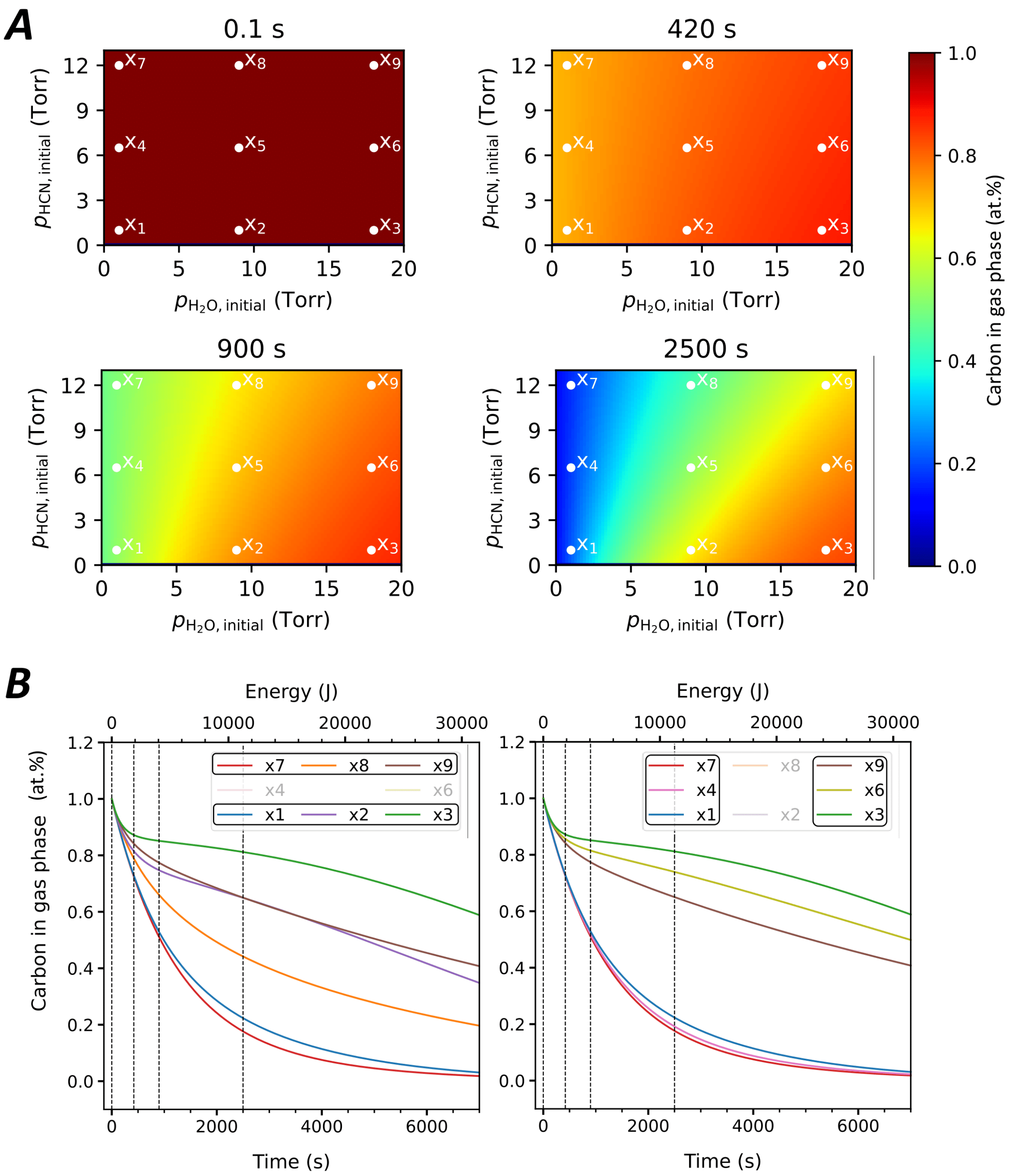

Figure S26: Amount of gas phase carbon partial pressure calculated from our model. Panel A shows results for four selected times, panel B shows results for selected compositions.

C in gas phase evolution (2)

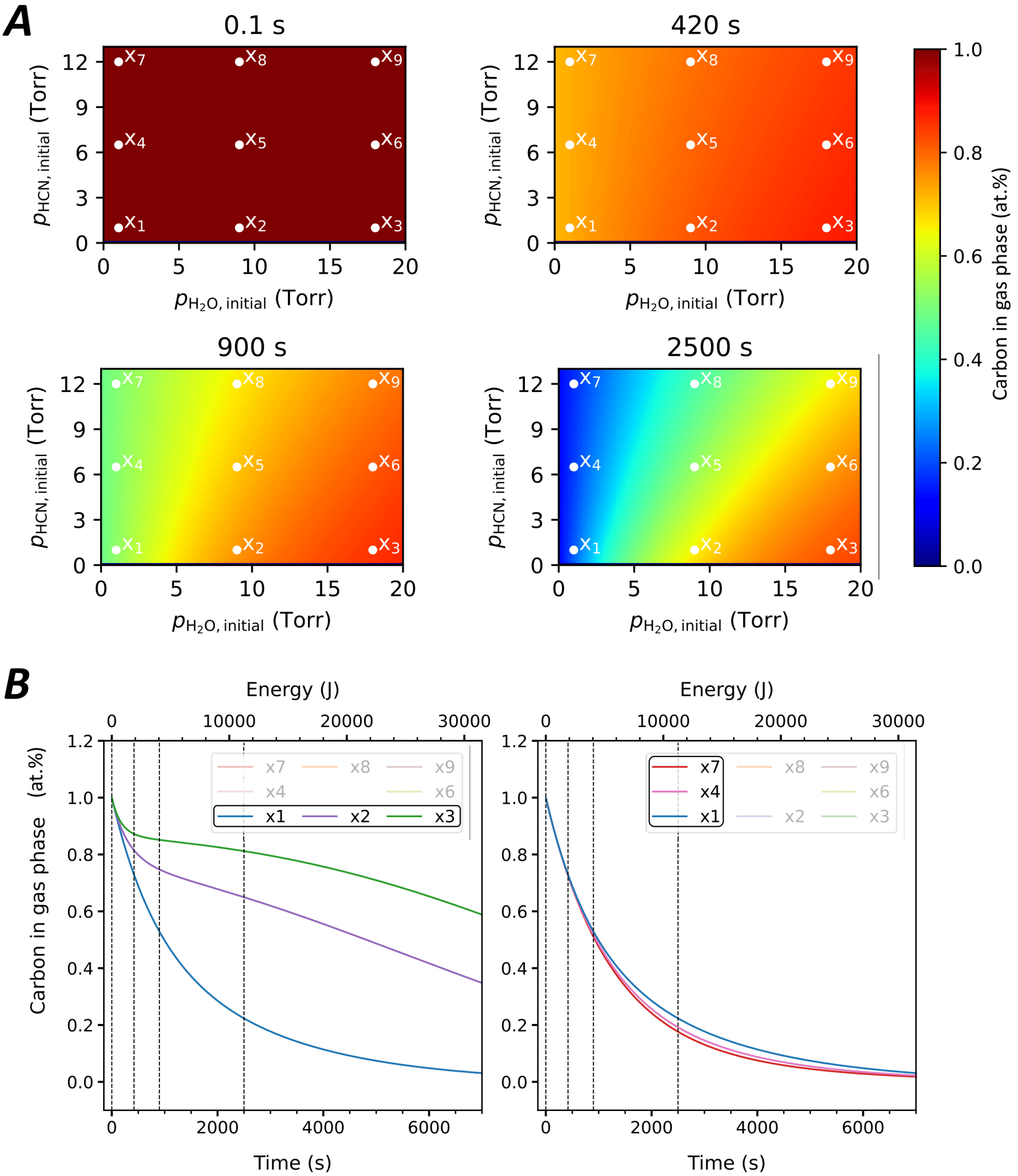

Figure S27: Amount of gas phase carbon partial pressure calculated from our model. Panel A shows results for four selected times, panel B shows results for different selected compositions.

C in gas phase evolution (3)

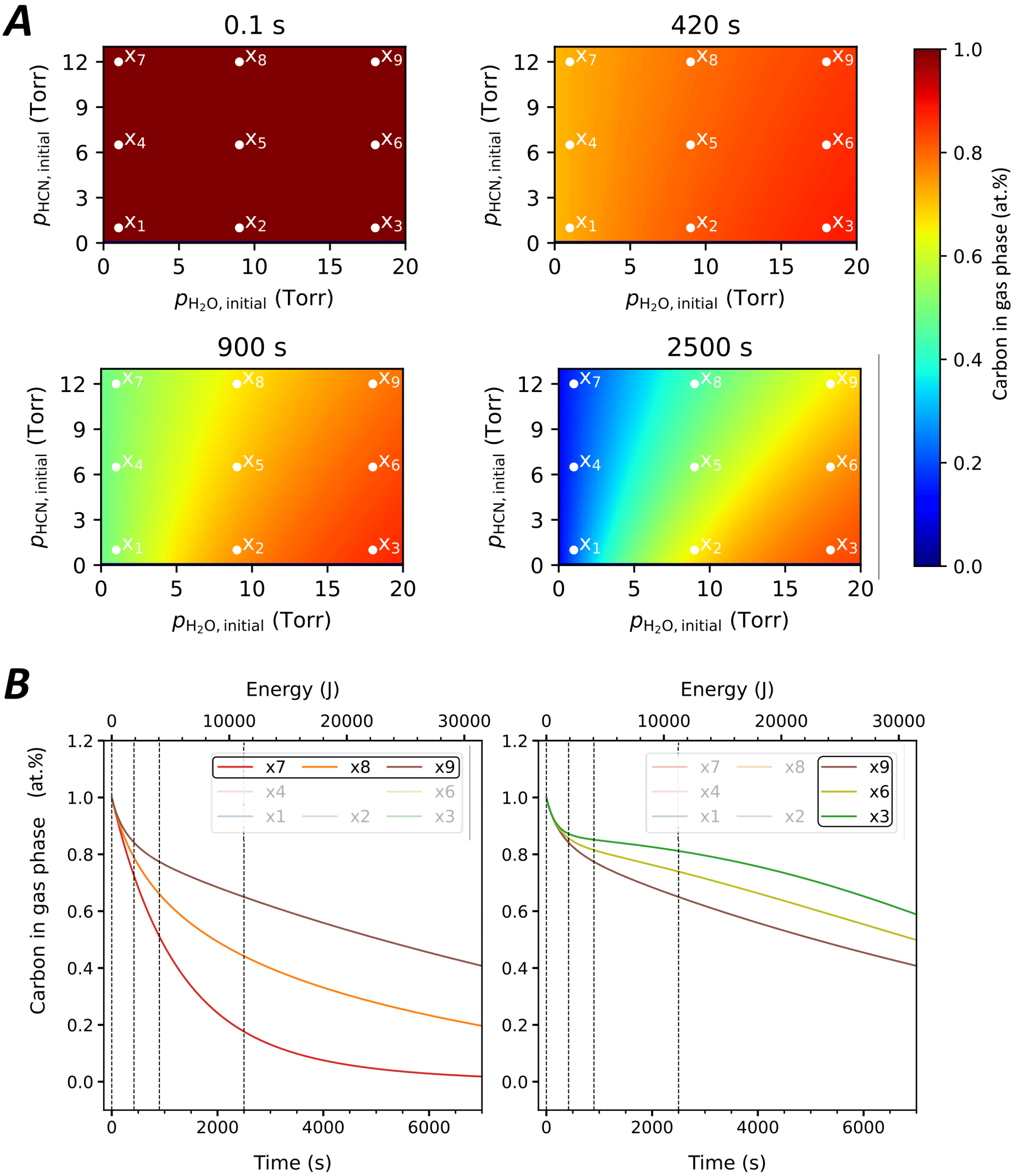

Figure S28: Amount of gas phase carbon partial pressure calculated from our model. Panel A shows results for four selected times, panel B shows results for different selected compositions.

C in gas phase evolution (4)

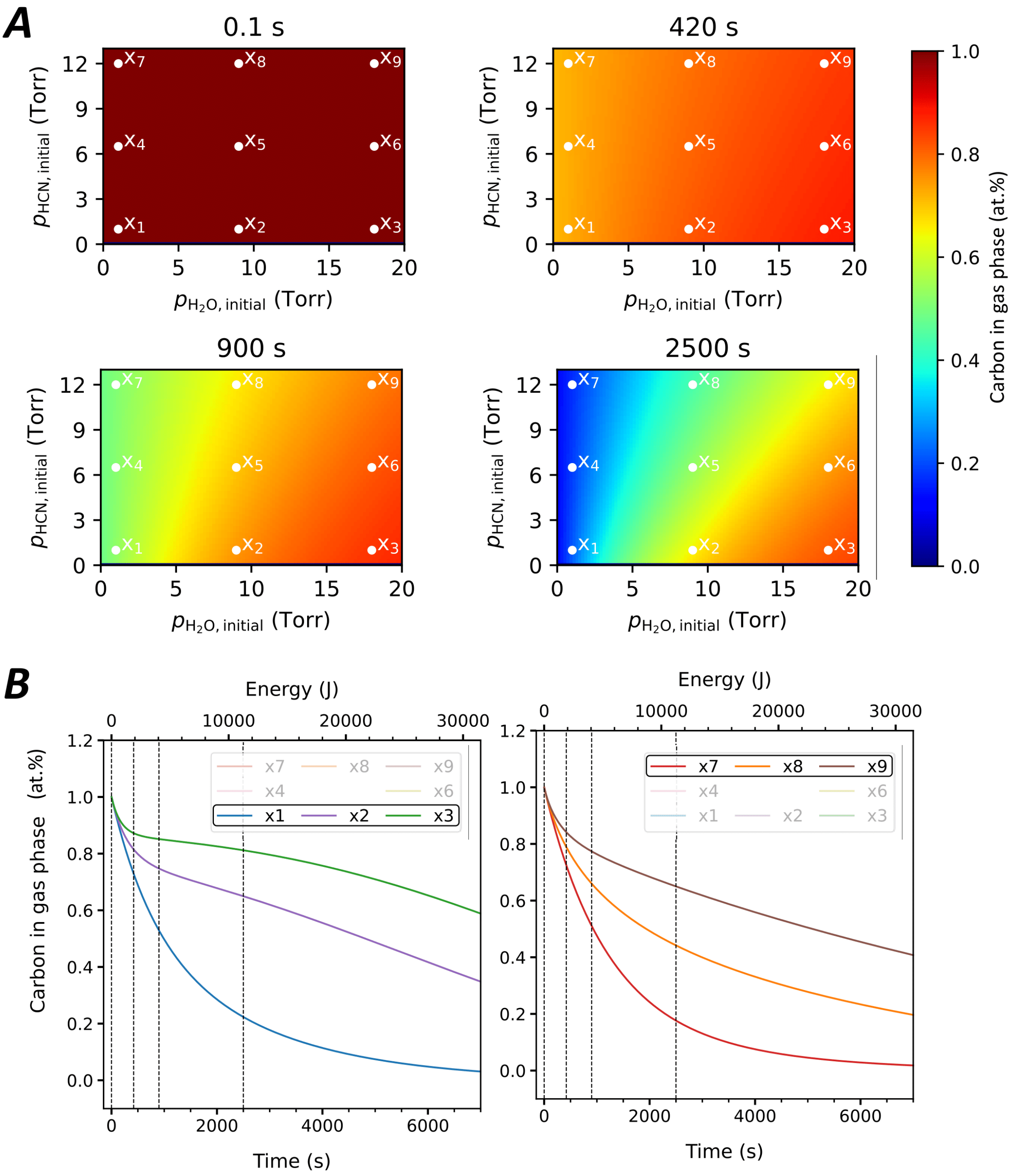

Figure S29: Amount of gas phase carbon partial pressure calculated from our model. Panel A shows results for four selected times, panel B shows results for different selected compositions.

C in gas phase evolution (5)

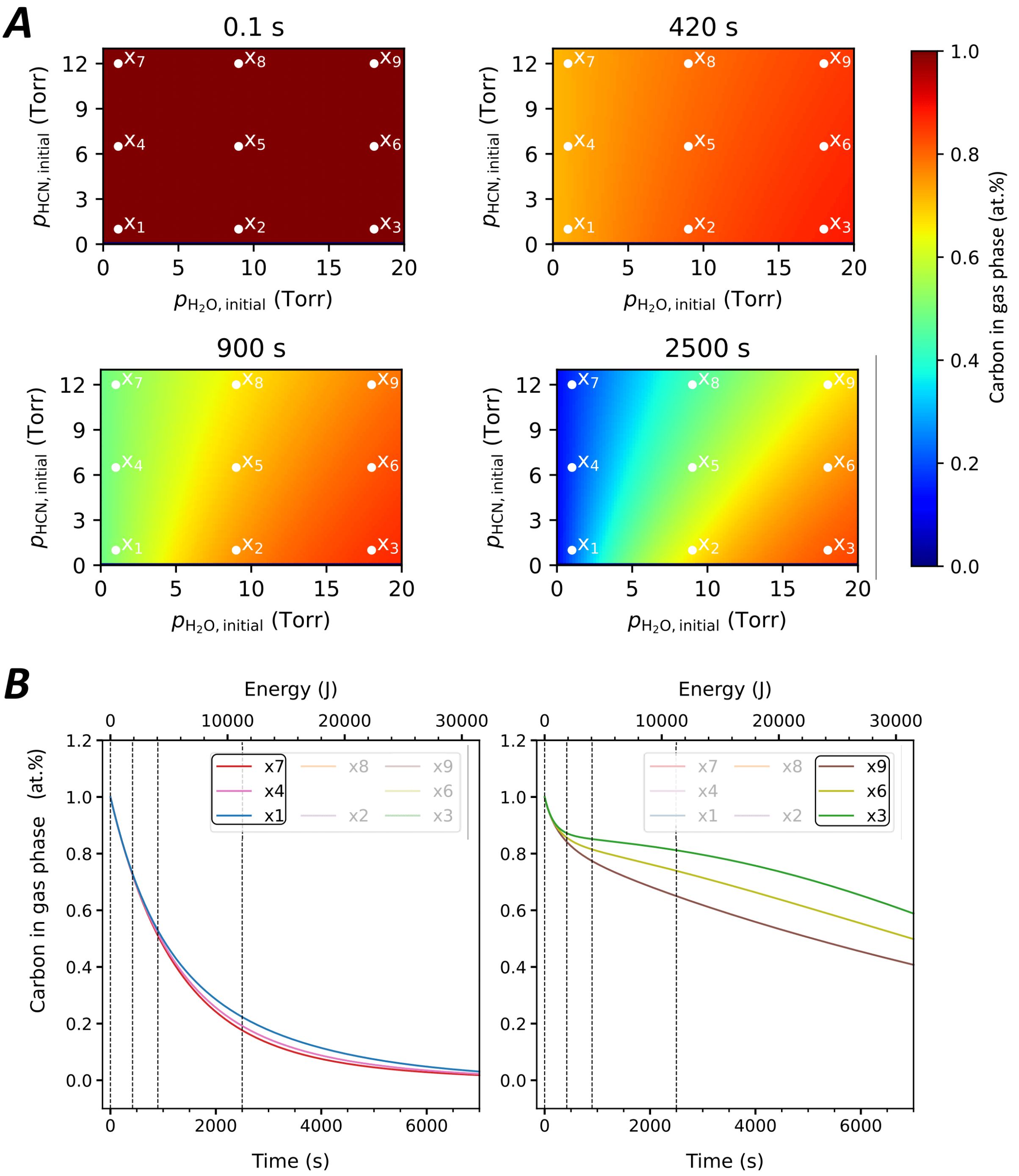

Figure S30: Amount of gas phase carbon partial pressure calculated from our model. Panel A shows results for four selected times, panel B shows results for different selected compositions.
